# Characterization of indole-3-pyruvic acid pathway-mediated biosynthesis of auxin in *Neurospora crassa*

**DOI:** 10.1371/journal.pone.0192293

**Published:** 2018-02-08

**Authors:** Puspendu Sardar, Frank Kempken

**Affiliations:** Abteilung Botanische Genetik und Molekularbiologie, Botanisches Institut und Botanischer Garten, Christian-Albrechts-Universität, Kiel, Germany; University of California Riverside, UNITED STATES

## Abstract

Plants, bacteria and some fungi are known to produce indole-3-acetic acid (IAA) by employing various pathways. Among these pathways, the indole-3-pyruvic acid (IPA) pathway is the best studied in green plants and plant-associated beneficial microbes. While IAA production circuitry in plants has been studied for decades, little is known regarding the IAA biosynthesis pathway in fungal species. Here, we present the first data for IAA-producing genes and the associated biosynthesis pathway in a non-pathogenic fungus, *Neurospora crassa*. For this purpose, we used a computational approach to determine the genes and outlined the IAA production circuitry in *N*. *crassa*. We then validated these data with experimental evidence. Here, we describe the homologous genes that are present in the IPA pathway of IAA production in *N*. *crassa*. High-performance liquid chromatography and thin-layer chromatography unambiguously identified IAA, indole-3-lactic acid (ILA) and tryptophol (TOL) from cultures supplemented with tryptophan. Deletion of the gene (*cfp*) that encodes the enzyme indole-3-pyruvate decarboxylase, which converts IPA to indole-3-acetaldehyde (IAAld), results in an accumulation of higher levels of ILA in the *N*. *crassa* culture medium. A double knock-out strain (*Δcbs-3*;*Δahd-2*) for the enzyme IAAld dehydrogenase, which converts IAAld to IAA, shows a many fold decrease in IAA production compared with the wild type strain. The *Δcbs-3*;*Δahd-2* strain also displays slower conidiation and produces many fewer conidiospores than the wild type strain.

## Introduction

Indole-3-acetic acid (IAA) is considered the main auxin in plants and a major regulator of plant growth and development. Although the physiological role of auxins in plants is well understood and several IAA biosynthesis pathways have been proposed, only a limited number of biosynthesis genes have been characterized to date despite decades of research [1, 2]. Tryptophan is considered the major source of IAA synthesis in plants, but in some cases, other indole derivatives such as indole-3-acetamide and indole-3-ethanol (TOL) are also employed [3–5]. IAA can either be produced from the precursor molecule tryptophan through several pathways involving different intermediates, or via a tryptophan-independent pathway [6]. Tryptophan-dependent pathways include the (i) indole-3-pyruvic acid (IPA), (ii) YUC flavin monooxygenase, (iii) indole-3-acetaldoxime (IAOx), (iv) indole-3-acetamide (IAM) and (v) tryptamine (TAM) pathways [1, 7, 8]. The most studied is the IPA pathway, which involves three steps. It is initiated by the oxidative transamination of tryptophan to IPA, proceeds via decarboxylation of IPA to indole-3-acetaldehyde (IAAld) by indole-3-pyruvate decarboxylase, and ends by converting IAAld to IAA as the final product via the enzymatic reaction of IAAld dehydrogenase. In the YUC flavin monooxygenase pathway, IPA is directly converted to IAA by the enzymatic action of the flavin monooxygenase-like proteins (YUC family), which has been proposed to be ‘the main auxin biosynthesis pathway in *Arabidopsis*’ [7]. IAAld serves as a key intermediate for two additional IAA biosynthesis pathways and can be generated directly from tryptophan or via the intermediate compound tryptamine [9].

Microorganisms, including bacteria and fungi, also produce IAA [10, 11]. Unlike the situation in plants, the physiological role of microbial auxins is quite unclear. A number of biosynthetic genes involved in microbial IAA production have been identified or discussed previously [12–14]. In pathogenic bacteria, the IAM pathway is considered the major route for IAA production, yet IAA synthesis via the IPA pathway has been described in a broad range of plant growth-promoting bacterial species [13, 15, 16]. These two pathways use different routes for IAA production via different intermediates. IAA production in different fungi has been documented and shown to be dependent on tryptophan [10, 11, 17, 18]. The IPA pathway-mediated IAA biosynthesis route has been conclusively demonstrated only in the smut fungus *Ustilago maydis* [15, 17], and genes have been identified for the same pathway in the ectomycorrhizal fungus *Tricholoma vaccinum* [19]. *U*. *maydis* and *T*. *vaccinum* are the only fungal species known to date from which IAA metabolic genes from the IPA pathway have been isolated and functionally characterized. In addition, the root endophyte *Piriformospora indica* has been shown to produce IAA and indole-3-lactic acid (ILA) through IPA as an intermediate compound. Elimination of the tryptophan aminotransferase gene from *P*. *indica* results in a significant reduction in IAA and ILA production [20]. A tryptophan-independent IAA biosynthetic pathway has not been described in most fungi and is known only from yeast species [21, 22]. In *Fusarium*, however, instead of IPA, the IAM pathway is utilized or IAA biosynthesis [14].

In recent literature, the IAA-producing genes and biosynthetic circuitry have been demonstrated exclusively in phytopathogenic or other plant-associated fungi. However, IAA, a basic signaling molecule, has been shown to play a role in interspecies communication and signal transduction [23–25]. This knowledge led us to explore the presence of IAA biosynthetic genes and a functional IAA biosynthesis pathway in the non-pathogenic fungus *Neurospora crassa*. IAA production in *N*. *crassa* has been shown and requires an external supply of tryptophan [18, 26]. We studied IAA biosynthetic genes present in the IPA, YUC, IAM and TAM pathways. Here, we identify, for the first time, *N*. *crassa* genes that contribute functionally to tryptophan-dependent IAA biosynthesis. These data provide initial insight in auxin production in a non-pathogenic filamentous fungus.

## Results

A few reports have described auxin production in the filamentous fungus *N*. *crassa* [18, 26–29]. However, none of these studies has investigated the genes required for IAA production. Our study aimed to comprehensively analyze the potential of *N*. *crassa* to produce auxin and to identify the pathway(s) and gene functionality using both bioinformatics and experimental analyses.

The amino acid sequences of four key enzymes from different fungal species, i.e., tryptophan aminotransferase, pyruvate decarboxylase, aldehyde dehydrogenase, and flavin-monooxygenase, were selected for sequence and structural analyses. Alignments of these enzyme sequences demonstrated that all the key amino acid residues that are important for binding to the respective ligands are conserved in the fungal enzyme sequences. In many cases, specific groups of proteins were identified and categorized by their ligand binding motifs, which are known as signature motifs for that particular group of proteins. We implemented a motif searching approach to identify specific ligand binding motifs and categorize the studied enzymes accordingly. Three copies of IAAld dehydrogenase (*cbs-3*, *ahd-3* and *ahd-2*) from *N*. *crassa* were identified and found in six other species. A single copy of each tryptophan aminotransferase (*aro-8*), pyruvate decarboxylase (*cfp*) and flavin-monooxygenase (*mox-2*) from *N*. *crassa* was identified and also identified in eight other species. Sequence-based functional conservation analysis suggested that these genes have been conserved throughout fungal evolution (**S1–S4 Figs**).

The *N*. *crassa* genes that were identified and used for bioinformatics as well as for experimental studies were *aro-8* (NCU09116), *cfp* (NCU02193), *cbs-3* (NCU03415), *ahd-3* (NCU09648), *ahd-2* (NCU00378) and *mox-2* (NCU03755). Site-specific binding of enzymes to their respective cofactors is essential to determine their active states. The ligand binding sites for the group of homologous enzymes analyzed in this study have been published previously [30–33]. Genes were named according to the *N*. *crassa* gene nomenclature and used throughout this study. For clarity, a conversion table is presented that includes the *N*. *crassa* genes and their corresponding general auxin gene nomenclature for the respective enzymes (details in **S1 Table** and the Materials and Methods section).

## Key amino acid residues that are important for enzymatic function are conserved

**Fig 1:**
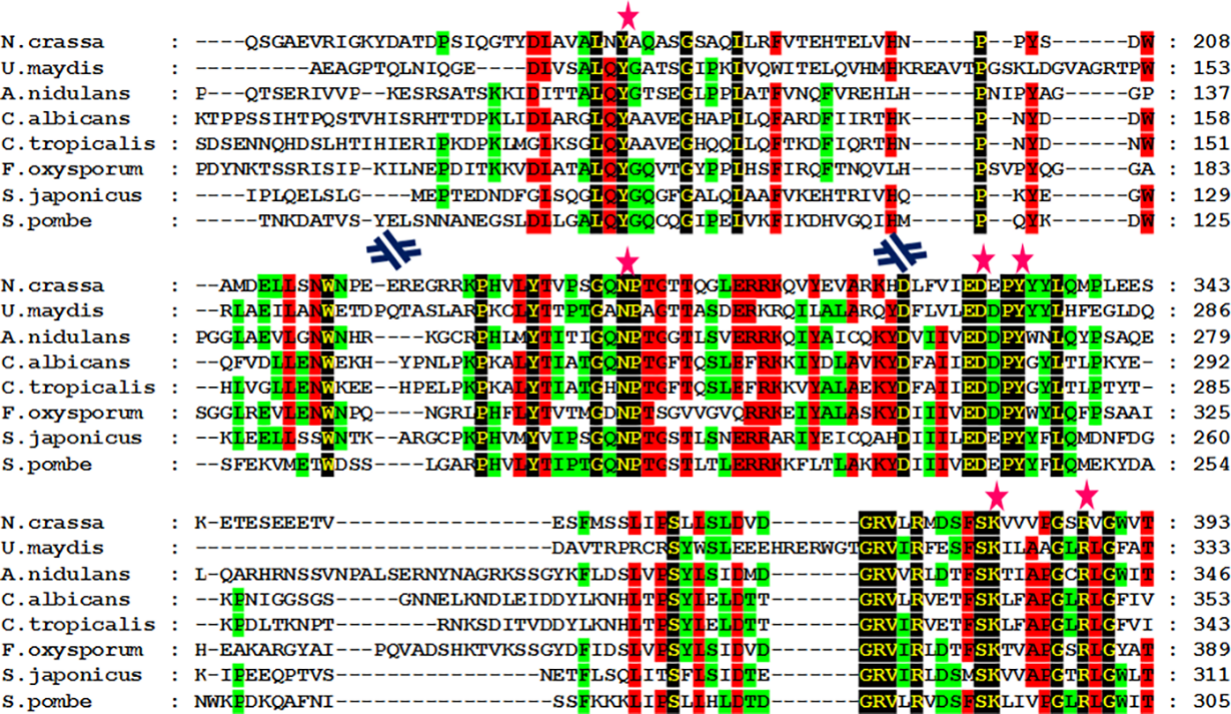
Multiple sequence alignment of tryptophan aminotransferase homologs from different fungal species. Residues with 100% identity are shown in yellow font with a black background, residues with 75% identity are shown in black font with a red background, and residues with 50% identity are shown in black font with a green background. PLP binding residues are marked with a pink star at the top. Sequences are presented in a discontinuous fashion. Blue discontinuous symbols are shown when there is a discontinuity of the sequence.

Although the alignments were carried out using multiple species, the focal species of our investigations was *N*. *crassa* because of the availability of thousands of knock-out mutants [34]. Aminotransferase enzymes are homologous to pyridoxal-5'-phosphate (PLP)-dependent enzymes, which share a common ancient evolutionary origin. They catalyze the deamination and racemization reactions of amino acids in different species lineages [35]. The amino acid residues that are known to bind to PLP are conserved in the tryptophan aminotransferase homolog in *N*. *crassa*. The data indicate that residues Y180, N302, D330, Y333, K381, and R388, present in the *N*. *crassa* homolog of tryptophan aminotransferase are involved in ligand binding (**Fig 1**). Pyruvate carboxylase catalyzes the carboxylation reaction of pyruvate to oxaloacetate. Thiamine pyrophosphate (TPP) acts as a ligand in the carboxylation reaction [36]. Residues G36, E59, T81, V84, G396, W419, G420, I422, V449, G450, D451, and G452 in the pyruvate decarboxylase homolog from *N*. *crassa* appeared to be significantly conserved when aligned with known ligand binding residues, and these residues were also highly conserved in other species (**S5 Fig**). NAD serves as a cofactor for aldehyde dehydrogenase enzymes. Compared with the known template, IAAld dehydrogenase from *N*. *crassa* was observed to contain the conserved amino acid residues that are important for NAD binding within the enzymatic catalytic site. The data suggest that the conserved residues from *N*. *crassa* aldehyde dehydrogenase involved in ligand binding were N167, T242, G243, E266, L267, G268, C300, E397, F399, and F463. These residues are also conserved in other species (**S6 Fig**). Flavin-monooxygenase or YUC family proteins are known to have an FAD binding signature motif known as a -GXGXXG- motif [30] (“x” denotes any amino acid). The data suggest that the MOX-2, the YUC homolog from *N*. *crassa* has a nucleotide binding motif consisting of G17, A18, G19, P20, A21, and G22 (**S7 Fig**).

**Fig 2:**
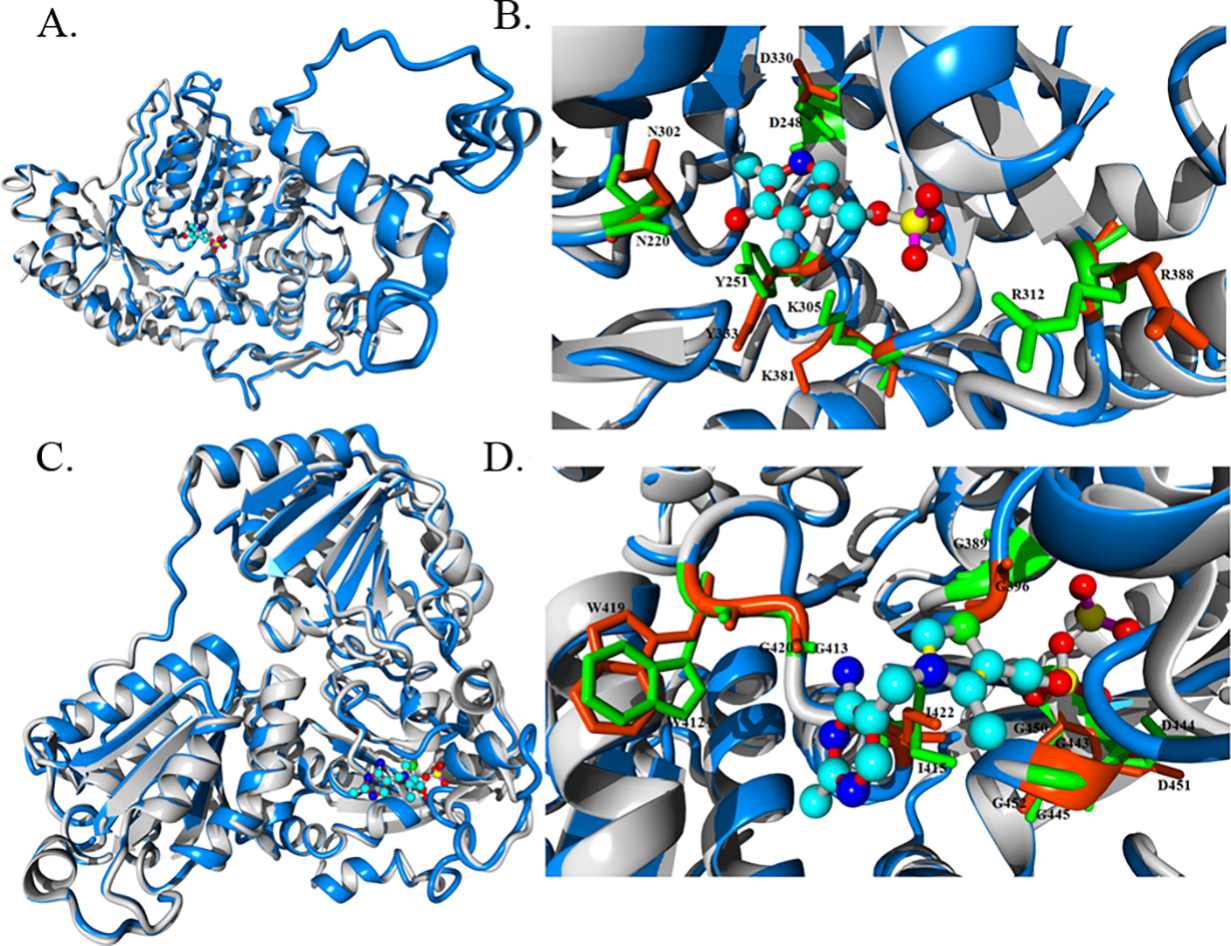
Structural alignment and characterization of ligand binding sites of predicted *N*. *crassa* enzymes. Predicted structures and known templates are shown in blue and gray, respectively. Residues present in predicted enzymes and templates are indicated in orange and green, respectively. All structures are rendered as a ribbon. Key amino acid residues involved in ligand binding are rendered in a stick model. Residues in close vicinity are only highlighted. Ligands are presented in a ball and stick model. (**A**) The overall structural alignment of predicted ARO-8 structure with a known enzyme structure (4JE5). PLP is bound inside the enzymatic catalytic site. (**B**) Structural insight into the ligand binding site of both the predicted and 4JE5 known structure. (**C**) Structural alignment of the predicted structure of the pyruvate decarboxylase homolog from *N*. *crassa* with a known pyruvate decarboxylase structure (2VJY). TPP is bound inside the enzymatic catalytic site. (**D**) Characterization of the ligand binding site of the predicted enzyme using the 2VJY structure as a template.

Together with the sequence analysis, we performed a structural analysis of candidate enzymes to provide evidence for their involvement in IAA biosynthesis. In this context, sequence and structural analysis data were supportive and complemented each other [37]. All key amino acid residues from each enzyme present in the auxin biosynthesis pathway were sequentially conserved in the focal species *N*. *crassa* as well as in other fungal species. Evidence for structural conservation of the enzymatic catalytic sites of the analyzed proteins is also provided. The results of the structural analysis are presented in two ways. First, the overall structure of the predicted protein model is aligned with a known enzyme with a known function (**Fig 2A** and **2B**). Then, an in-depth analysis of each amino acid residue present in the enzymatic catalytic site and responsible for ligand binding is provided (**Fig 2B** and **2D**). The same analysis was performed for ARO-8 and CFP (**Fig 2**), IAAld dehydrogenase and MOX-2 (**S8 Fig**). We checked for the putative ligand binding site in the predicted structures using COFACTOR [38]. COFACTOR *in silico* analysis makes predictions about the ligand binding sites and plausible ligands that can bind to the given protein structure.

COFACTOR analysis of the putative *N*. *crassa* aromatic aminotransferase (ARO-8) suggests that it has a strong binding match (BS-score 1.20) for PLP (a BS-score >1 signifies strong binding). PLP serves as a coenzyme in transamination, decarboxylation, and deamination reactions of amino acids. The aromatic aminotransferase Aro8 enzyme in a complex with PLP from *Saccharomyces cerevisiae* [39] was used as a template (PDB ID: 4JE5) for the structural alignment. A structural alignment between the predicted ARO-8 from *N*. *crassa* and the template structure showed that despite the presence of sequence dissimilarities, protein folding was very similar in both protein structures (**Fig 2A**). Analysis of the PLP binding site revealed key amino acid residues that were positionally conserved in the predicted structure and mimicked a conformation similar to the template structure, which facilitated proper ligand binding (**Fig 2B**). Analysis of the ligand binding of the predicted structure of pyruvate decarboxylase from *N*. *crassa* indicated that thiamine pyrophosphate (TPP) had a greater potential to serve as a ligand (BS-score 1.46). Along with many other enzymatic reactions, TPP is known as a coenzyme for pyruvate decarboxylase. To explore the ligand binding site and compare the overall topology, pyruvate decarboxylase from *Enterobacter cloacae* [40] was used as a structural template (PDB ID: 1OVM). Structural alignment of pyruvate decarboxylase from *N*. *crassa* with the above-mentioned template (**Fig 2C**) indicated that the TPP binding site was similar and that all the key amino acid residues were positionally conserved in the enzymatic active site (**Fig 2D**). Aldehyde dehydrogenase (IAD) is grouped within a wide class of enzymes, namely, the dehydrogenases. We observed a good amount of sequence conservation among three of the IAD enzymes from *N*. *crassa* (CBS-3, AHD-3 and AHD-2) itself, and therefore only the CBS-3 structure is shown herein. The NAD binding pocket was also found in the predicted CBS-3 homolog of *N*. *crassa* and was compared with the known NAD binding enzyme [41] (PDB ID: 1NZX) (**S8 Fig**). MOX-2, which is also known as flavin-containing monooxygenase (FMO), is known to be functional when bound to the NADPH-FAD complex. The nucleotide binding sequence GAGPSG from *S*. *pombe* stabilizes the binding of FAD [30]. The present results showed that the putative FMO from *N*. *crassa* folded in such a fashion that the GXGXXG motifs (‘X’ denoting any amino acid) of both the template (PDB ID: 1VQW) and predicted structure were properly aligned (**S8 Fig**).

### IAA production in N. crassa is tryptophan dependent

**Fig 3:**
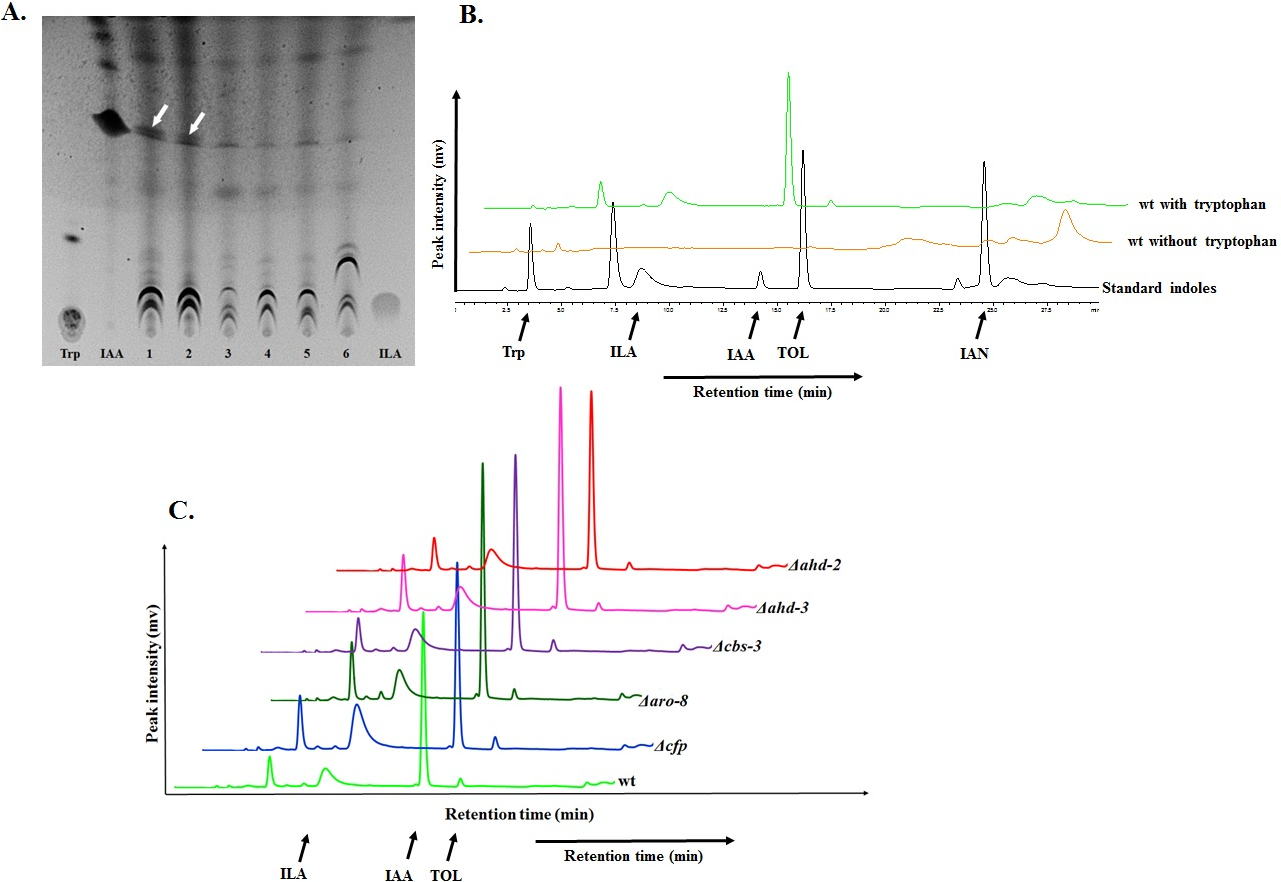
IAA and related indolic compound production by *N*. *crassa* upon tryptophan supplementation. N. crassa produces IAA and other related indolic compounds when supplemented with tryptophan. (**A**) TLC analysis to find the lowest concentration of tryptophan that can be used by N. crassa to produce IAA. White arrows in lane 1 and 2 indicate traces of IAA. Different tryptophan concentrations were used in lane 1 (5 μM), 2 (1 μM), 3 (100 nM), 4 (50 nM), 5 (25 nM), and 6 (without tryptophan). (**B**) HPLC analysis of the metabolites produced by wild type N. crassa with and without tryptophan supplementation for 72 hours. (**C**) Metabolite profiling of wild type as well as single knock-out N. crassa strains using HPLC. Single knock-outs for the genes specifically present in the IPA pathway of IAA biosynthesis were used. The following standards were used: tryptophan (Trp), indole-3-lactic acid (ILA), indole-3-acetic acid (IAA), tryptophol (TOL) and indole-3-acetonitrile (IAN).

It is known that external supplementation with tryptophan leads to IAA biosynthesis in *N*. *crassa* [26, 42]. In this study, for the first time we have analyzed the production of a wide range of indolic compounds including IAA, ILA and TOL by *N*. *crassa* employing different chromatographic techniques. *N*. *crassa* strains grown without supplementation of external tryptophan show no traces of IAA in the sample-extracts (**S9 Fig**). While similar data have been reported earlier [18], those data were based on the *Avena* curvature test. Tryptophan feeding tests were performed with various concentration of tryptophan to identify the threshold concentration of tryptophan in IAA production by *N*. *crassa*. Cultures without any supplementation were used as control. Compounds were extracted from the culture and loaded onto a silica plate along with standard indolic compounds for TLC assay. We found that *N*. *crassa* can produce IAA with externally supplemented tryptophan in as low concentration as 1 μM (**Fig 3A).** Lower concentrations of 100, 50 and 25 nM tryptophan produced no detectable IAA. Total indole including IAA production by the wild type *N*. *crassa* strain increases with increasing concentration of tryptophan supplementation (**S10 Fig)**.

For HPLC analysis, a fixed tryptophan concentration level of 400 mg/L (1.96 mM) was used. The 72-hour-old wild type fungal cultures supplemented with and without tryptophan were analyzed by HPLC. We found that cultures without tryptophan supplementation did not produce any of the indoles, while HPLC peaked for different indolic compounds over time from the tryptophan supplemented culture, which were identified based on the corresponding standards that were run along with the samples (**Fig 3B**). We found that among all the indolic compounds that were analyzed, IAA was the most abundant compound. Apart from IAA, indole-3-lactic acid and tryptophol were also identified under the same cultural and experimental conditions.

### Towards pathway elucidation in N. crassa

**Fig 4:**
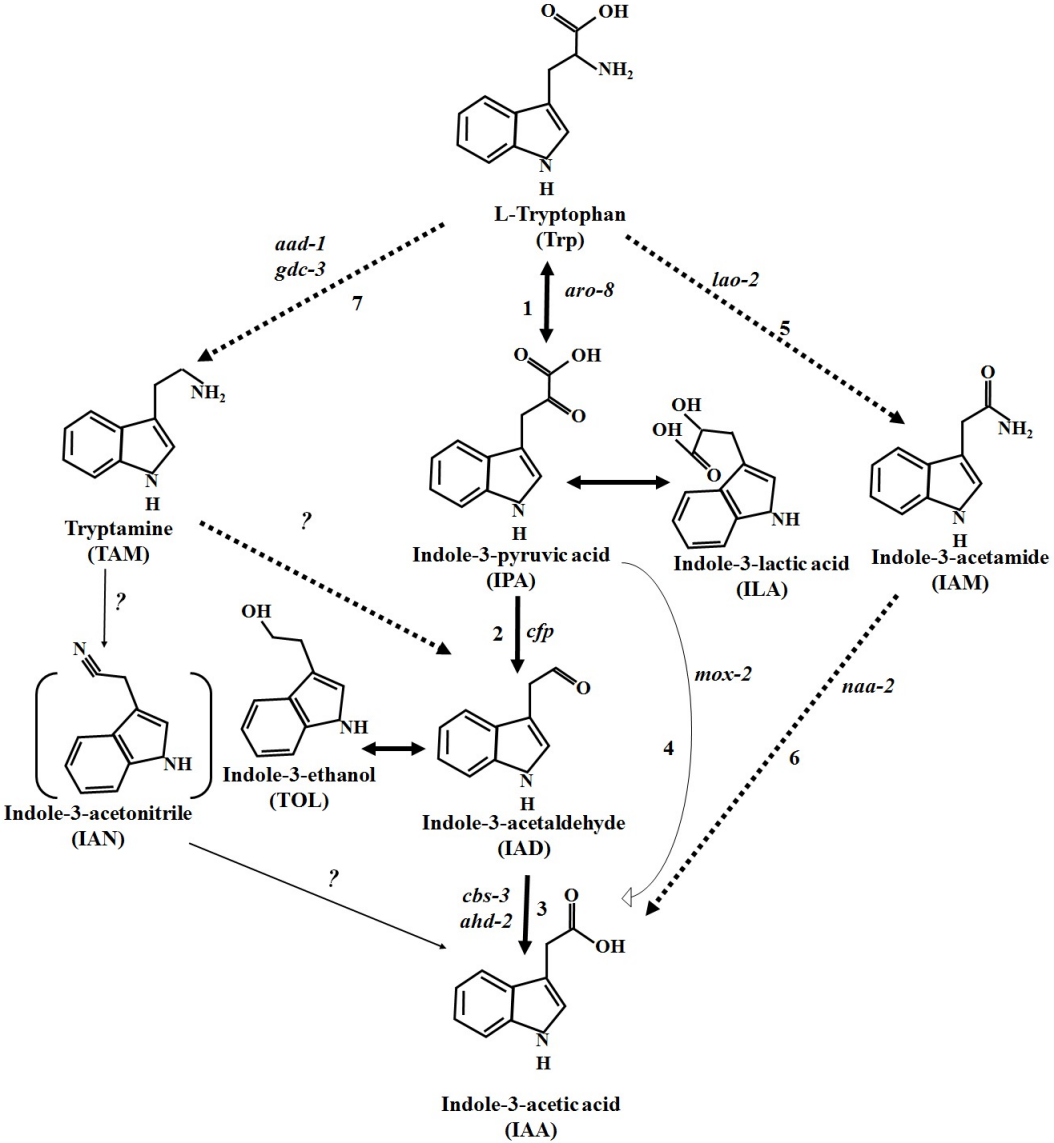
Proposed tryptophan-dependent IAA biosynthetic pathway in *N*. *crassa*. The pathway with solid bold arrows was functionally characterized by the identification of novel genes, and intermediate compounds were also identified and estimated. Pathways with dashed bold arrows were proposed accordingly based on the feeding test with IAM and TAM. Solid thin arrows denote pathways proposed in green plants but not known in fungi including *N*. *crassa*. Unidentified homologues of the genes in *N*. *crassa* for the respective biosynthetic steps are indicated by question marks. IAN, with bracketed lines, has not been identified in the *N*. *crassa* culture. All other indoles without any brackets (except IAM and TAM) were identified from the culture upon tryptophan feeding. Indole-3-pyruvic acid (IPA) and indole-3-acetaldehyde (IAAld) are very unstable compounds and spontaneously convert into indole-3-lactic acid (ILA) and indole-3-ethanol (TOL), respectively. ILA and TOL can be readily identified in culture as a proxy for IPA and IAAld, respectively. Numbers in the figure reflect different enzymes: 1—tryptophan aminotransferase, 2—pyruvate decarboxylase, 3—aldehyde dehydrogenase, 4—flavin monooxygenase, 5—L-amino acid oxidase, 6—N-acylethanolamine amidohydrolase, 7 –aromatic-L-amino acid decarboxylase and glutamate decarboxylase.

In bacteria and plants, many IAA biosynthetic genes have been identified, and as a result, many pathways have also been proposed. In this study, we performed, for the first time, a comprehensive analysis of *N*. *crassa* encompassing different IAA biosynthesis pathways that were previously proposed in microorganisms and plants. We identified IAA biosynthetic genes from different pathways in *N*. *crassa* and placed them in the IAA producing network for a better understanding of the IAA-producing circuitry (**Fig 4**). We took advantage of this proposed IAA-producing pathway for the systemic analysis of IAA producing gene(s).

### The *cfp* gene is one of the key regulators of the IPA pathway in *N*. *crassa*

**Fig 5:**
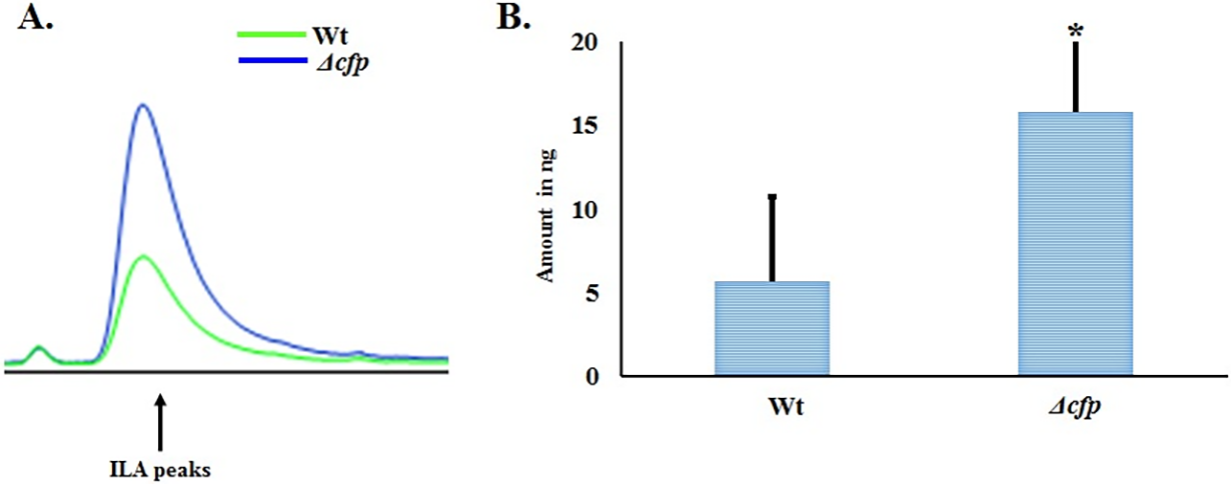
Effect of the *Δcfp* strain on ILA production in *N*. *crassa*. (**A**) HPLC peaks (peak intensity is given in mv as in **Fig 3**) of indole-3-lactic acid (ILA) from the wild type and Δcfp strain are shown in different colors. (**B**) Amount of ILA produced in 72-hour cultures of wild type and Δcfp knock-out strains supplemented with tryptophan. * p < 0.05.

**Fig 6:**
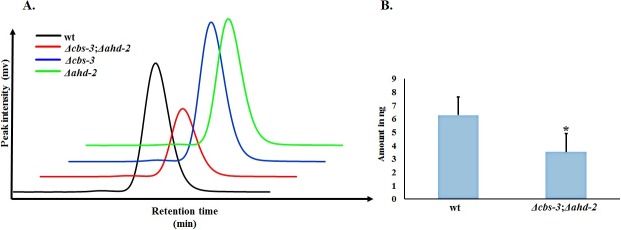
Reduction in IAA production by the synergistic effect of the *Δcbs-3*;*Δahd-2* double knock-out in *N*. *crassa*. Individual knock-out strains for the aldehyde dehydrogenase genes cbs-3 and ahd-2 did not have any significant effect on IAA production, but the synergistic effect of the Δcbs-3;Δahd-2 double knock-out resulted in a strong reduction of IAA production. (**A**) HPLC peaks of IAA for wild type along with other knock-out strains are shown in different colors as mentioned in the figure. IAA production was decreased in the Δcbs-3;Δahd-2 double knock-out strain compared with the wild-type strain. (**B**) Comparison of the amount of IAA produced by wild type and Δcbs-3;Δahd-2 strains in 72 hours. * p < 0.05.

We found that, irrespective of their genetic background, all the strains produced IAA and related indolic compounds, but few knock-out strains produced indoles in higher or lower abundance (**Fig 3C**). HPLC analysis of 72-hour fungal cultures supplemented with 400 mg/L (1.96 mM) tryptophan revealed that the *Δcfp* knock-out strain produced a maximum amount of indole-3-lactic acid (**Fig 5A)**. We estimated that the amount of ILA was 64.4% higher in *Δcfp* (15.78 ng) compared with wild type (5.62 ng) (**Fig 5B**). In the pathway analysis of knock-out strains, the most pronounced effect on IAA production was found in the *Δcbs-3*;*Δahd-2* double knock-out strain (**Fig 6**). HPLC peaks for wild type, *Δcbs-3*, *Δahd-2* and *Δcbs-3*;*Δahd-2* are shown in different colors, as mentioned in the figure (**Fig 6A**). Based on the HPLC peak areas and those of the standards, we estimated that the amount of IAA in the *Δcbs-3*;*Δahd-2* double knock-out strain (3.52 ng) was 44% lower than in the wild type strain (6.28 ng) (**Fig 6B)** under the same cultural and extraction conditions. The other genes that were studied in the IAA biosynthesis pathways were *mox-2*, *lao-2* (tryptophan-2-monooxygenase), *naa-2* (indole-3-acetamide hydrolase) *aad-1* (aromatic–L-amino acid decarboxylase) and *gdc-3* (glutamate decarboxylase). Neither the single knock-outs nor any of the double or triple knock-out strains had any effect on the level of IAA production (**S12 Fig**).

### Fewer conidiospores in the *Δcbs-3*;*Δahd-2* double knock-out strain compared to wild type

**Fig 7:**
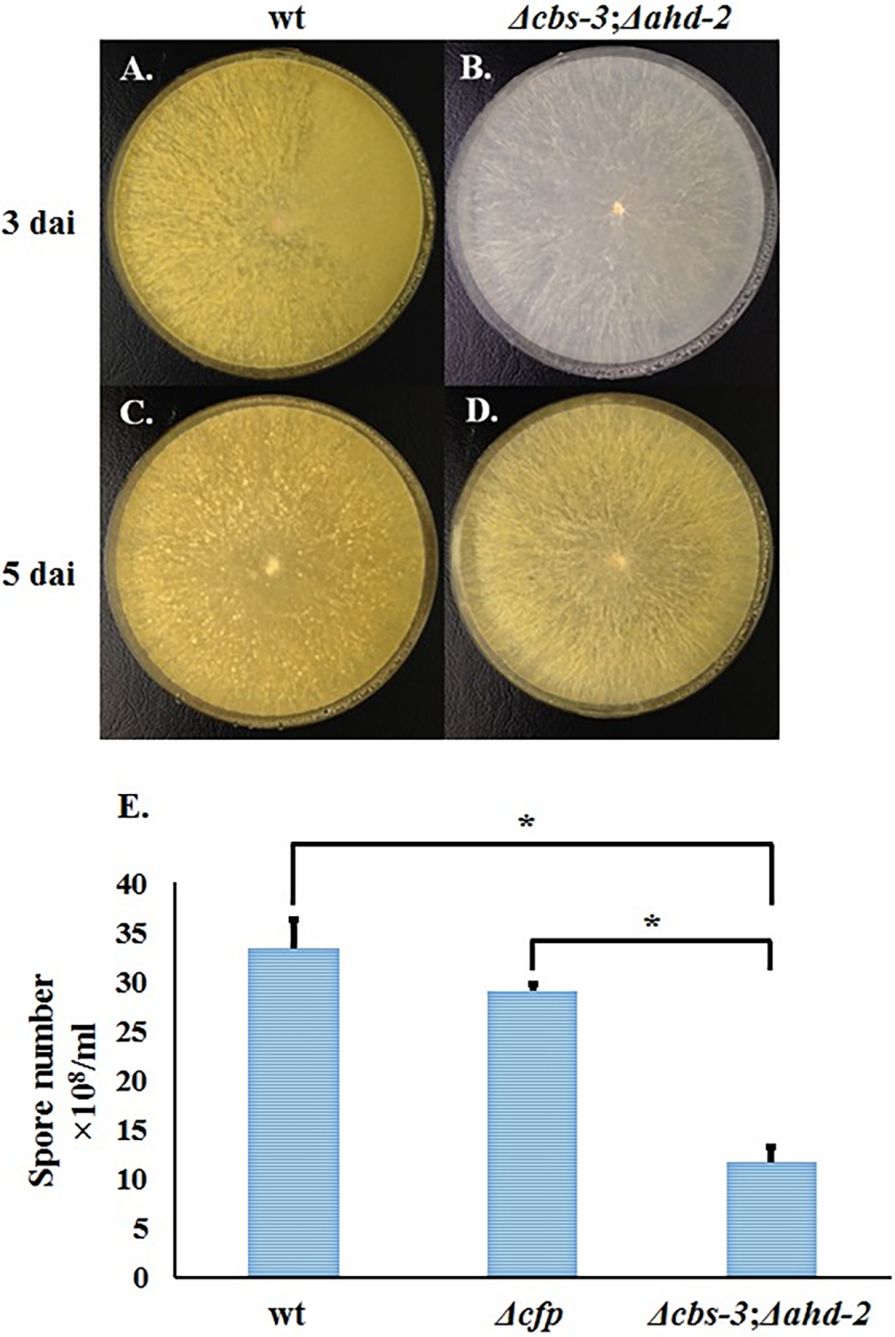
Conidiospore production by the *Δcbs-3*;*Δahd-2* double mutant. (**A**) and (**C**) are wild type strains, and (**B**) and (**D**) are Δcbs-3;Δahd-2 double knock-out strains after growth for three and five days, respectively. (**E**) A numerical comparison of conidia production by the Δcbs-3;Δahd-2 double knock-out strain with wild-type and the Δcfp single knock-out strain. dai: days after inoculation. * p < 0.05.

We observed the lowest amount of IAA production in the *Δcbs-3*;*Δahd-2* double-knock-out strain. We also checked its ability to produce conidia by culturing wild type and knock-out strains on an agar plate containing Vogel’s Minimal Medium (VMM). The double knock-out strain (**Fig 7B** and **7D**) for the genes *Δcbs-3* and *Δahd-2* showed much less conidiation than their wild type counterpart (**Fig 7A** and **7C**), even after five days of inoculation. After culturing on solid medium for 10–15 days, the total number of conidiospores produced by the *Δcbs-3*;*Δahd-2* strain was less than that of the wild type strain (**Fig 7E**).

### Indole production in *N*. *crassa* is time-dependent and affected by knock-out of the IAA-producing genes

IAA production in fungi is time-dependent, and various indolic compounds start to accumulate in the culture medium over time [20, 42]. In this study, we demonstrated how IAA and related indole production changes over time in different *N*. *crassa* strains with different mutations of the IAA-producing genes (**S13 Fig**). Our results suggested that the production of IAA and other related indolic compounds by the wild type strain in culture medium supplemented with tryptophan began after ~12 hours of inoculation and declined over time (**S13A Fig**). The *Δcfp* strain showed a similar indole production pattern as the wild type strain, with notably higher ILA production throughout the experimental time frame (96 hours) (**S13B Fig**). The *Δcbs-3*;*Δahd-2* double knock-out strain started to produce IAA later than the wild type strain (**S13C Fig**). None of the 72-hour-old fungal cultures showed any trace of tryptophan in HPLC and TLC analysis (**Fig 3B** and **S13A Fig**), but the *Δcbs-3*;*Δahd-2* double knock-out strain showed an accumulation of tryptophan up to 48 hours after inoculation compared with the wildtype strain (**S13C Fig** and **S13D Fig**). These results suggest that the *Δcbs-3*;*Δahd-2* strain had not only a reduced level of IAA production but also a lower efficiency in tryptophan utilization.

### Feeding test performed with other indolic compounds and the amino acid tyrosine

**Fig 8:**
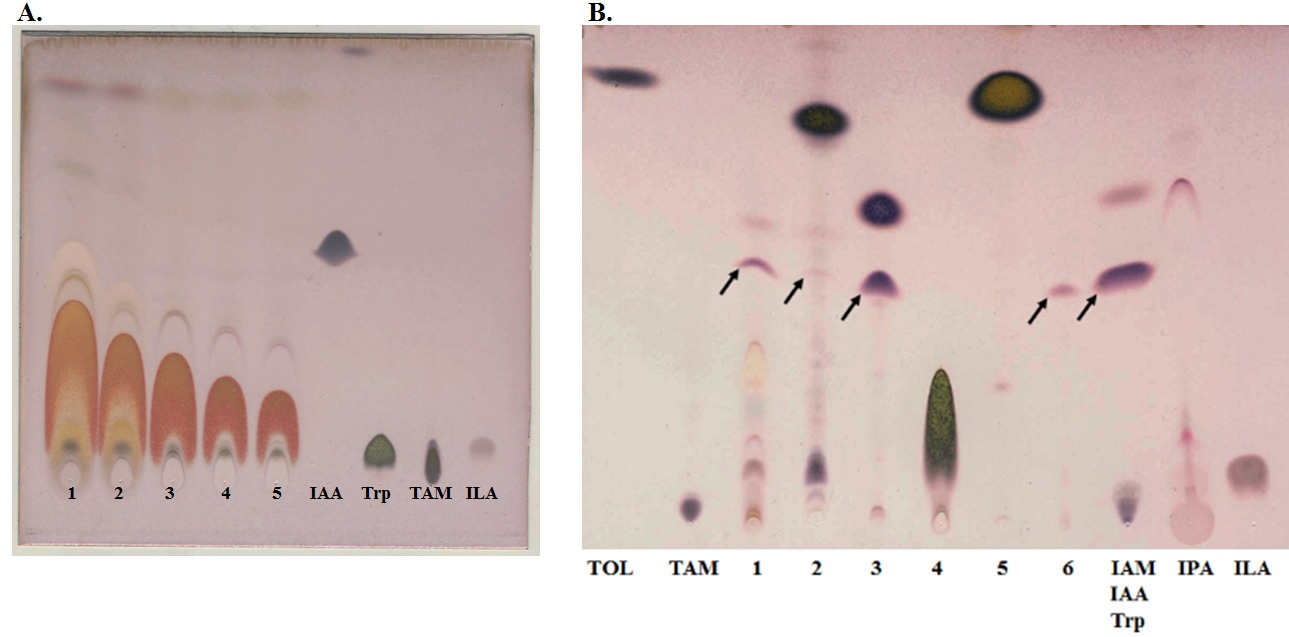
Indole production by the wt *N*. *crassa* strain supplemented with tyrosine and other indoles. Indole production by wt *N*. *crassa* was checked by supplementation with different concentrations of tyrosine. (**A**) lanes 1–5 tyrosine in 5 mM, 2.5 mM, 1.25 mM, 625 μM and 312.5 μM. (**B**) TLC analysis to determine the ability to produce indoles, including IAA, by various indolic compounds. Black arrows indicate traces of IAA. Indolic compounds that were used as supplements are as follows: lane 1—mM tryptophan, lane 2–2.5 mM tryptamine, 3–1 mM indole-3-acetamide, 4–1 mM indole-3-lactic acid, 5–1 mM tryptophol, 6–10 μM IAA. Following standards were used: tryptophol (TOL), tryptamine (TAM), indole-3-acetamide (IAM), indole-3-acetic acid (IAA), tryptophan (Trp), indole-3-pyruvic acid (IPA) and indole-3-lactic acid (ILA).

In an earlier study, it was mentioned that tyrosine may act as a precursor for IAA production in fungi [18], while some other studies utilized tryptamine. However, none of the tested fungi showed IAA production when supplemented with tryptamine [18, 20]. We performed feeding experiments with tyrosine together with indolic compounds such as tryptamine, indole-3-acetamide (IAM), indole-3-lactic acid, tryptophol (TOL) and indole-3-acetic acid (IAA) to assess their ability to produce IAA. Our results showed that the wild type strain could not produce IAA even when supplemented with tyrosine at a relatively high concentration of 5 mM. (**Fig 8A**). The effect of tyrosine was also checked by feeding the wild type *N*. *crassa* strain with different tyrosine concentrations and performing subsequent metabolite analysis on TLC plates. However, tyrosine-mediated IAA biosynthesis was not found in the same experimental setup with any of the concentrations used. In contrast, both tryptamine and IAM supplementation led to IAA production, although at different concentrations: 1 mM IAM was sufficient to induce IAA production (**S14 Fig**), but tryptamine had to be supplemented at 2.5 mM (**Fig 8B**).

The tryptophan supplemented culture produced a maximum number of indolic compounds compared to other supplements tested in this study (**Fig 8B** and **S14 Fig**). We also observed that indolic compounds, including IAA from the tryptophan-supplemented culture, gradually declined over time (**S13A Fig** and **S14 Fig**), but the opposite was observed for the IAM-supplemented culture (**S14 Fig**), in which the IAA level tended to increase. These results clearly suggest that not only tryptophan but also tryptamine and indole-3-acetamide (IAM), the main precursor molecule for bacterial IAA biosynthesis [13, 16], can be utilized as a precursor molecule for IAA biosynthesis in *N*. *crassa*.

## Discussion

Microorganisms, including fungi and bacteria, are capable of producing indole-3-acetic acid [1, 5, 10, 14, 17, 18, 20, 26, 43]. While many fungi have been shown to produce IAA using various IAA biosynthetic routes [5, 14, 15, 20], a limited number of IAA biosynthetic genes have been characterized in detail despite many efforts [1, 44]. IAA synthesis via the IPA pathway has been described in a broad range of plant growth-promoting bacterial species [13, 16] and in a few fungal species [15, 19, 20]. However, the presence of IAA in *N*. *crassa* has been documented in only a few studies [26, 42], and neither the biosynthetic genes nor the biosynthesis pathway have been explored. Here, we showed for the first time, that genes in the IPA pathway of IAA production are present and functionally active in *N*. *crassa*. Although a homologue of the gene encoding indole-3-pyruvate decarboxylase (*cfp*) has not been found in green plants [1], our results clearly demonstrated that *cfp* is present in a wide range of fungi. Furthermore, it appears to be fully functional in *N*. *crassa*. TLC and HPLC analyses of *N*. *crassa* wild type and knock-out strains for the IPA pathway used in this study revealed that *N*. *crassa* produces IAA via the IPA route. Additionally, we report that deletion of the IAA-producing genes in the IPA pathway of *N*. *crassa* results in altered production of IAA as well as other related indolic compounds. Tryptophan is needed as an external supplement for IAA production in *N*. *crassa* [26, 42]. We also assessed the potential for tyrosine to produce IAA because tyrosine has been mentioned as a potential precursor candidate leading to IAA in fungi [18]. However, our result confirmed that *N*. *crassa* cannot use tyrosine as a precursor molecule to produce IAA (**Fig 8A**).

### Identification and functional characterization of indole-3-pyruvate decarboxylase in *N*. *crassa*

By employing homology searching, we identified a pyruvate decarboxylase gene in *N*. *crassa* (NCU02193), which is named as “cellular filamentous protein” (*cfp*) in FGSC as well as in “FungiDB”. Herein we report that *cfp* from *N*. *crassa* contains binding sites for thiamine pyrophosphate (TPP or ThPP), which acts as a ligand for decarboxylase enzymes (**S5 Fig**, **Fig 2C** and **2D**). In the proposed IPA pathway from various studies [1, 32], it has been shown that indole-3-pyruvic (IPA) acid is an unstable compound and that a dynamic conversion occurs between IPA and indole-3-lactic acid (ILA). Our HPLC analysis showed that a *Δcfp N*. *crassa* strain produced more ILA as compared with the wild type strain (**Fig 5**). Hence, our results are consistent with the observed inability of fungi to convert IPA into indole-3-acetaldehyde (IAAld) in the absence of the *cfp* gene, leading to increased IPA levels and hence further accumulation of ILA. These results unambiguously confirm that *cfp* is present and functionally contributes its enzymatic role in the IPA pathway mediated IAA biosynthesis in *N*. *crassa*.

### Indole-3-acetaldehyde dehydrogenase genes are direct contributors to IAA production in *N*. *crassa*

The functional identification and synergistic effects of two IAAld dehydrogenase genes in the pathogenic fungus *U*. *maydis* have been previously studied [17]. In our study, we reported the presence of more than one copy of the genes encoding IAAld dehydrogenase in *N*. *crassa* (*cbs-3*, *ahd-2 and ahd-3*) and their functional contribution towards IAA biosynthesis. Individual knock-outs of any of these genes did not have any significant effect on IAA production (**Fig 6**). A significant decrease in IAA formation was only observed in the *Δcbs-3*;*Δahd-2* double knock-out *N*. *crassa* strain. These findings align perfectly with the same scenario in a previous study conducted in *U*. *maydis* [15]. A single copy IAAld dehydrogenase (*iad*) mutant did not cause any reduction in IAA formation in *U*. *maydis*, while an *iad1iad2* double mutant strain exhibited a many fold reduction in IAA formation [15, 17]. Taken together, these results support the presence of multiple highly interconnected and well regulated aldehyde dehydrogenase genes acting in the IAA biosynthesis pathway of *N*. *crassa*. The synergistic effect reflected in the *Δcbs-3*;*Δahd-2* double knock-out implies that IAA production in *N*. *crassa* via the IPA pathway proceeds through the production of indole-3-acetaldehyde (IAAld) and that the last step of this pathway is under tight regulation by multiple genes. Here, we report, for the first time, a novel aldehyde dehydrogenase gene (*ahd-2*) and its contribution towards IAA biosynthesis in *N*. *crassa*.

### IAA biosynthesis in *N*. *crassa* is activated and regulated spatiotemporally

In the present study, we examined IAA production by *N*. *crassa* with different supplements at different time points. Our results demonstrated that tryptophan is the only amino acid among tryptophan, tyrosine and histidine that can be used as a precursor for IAA biosynthesis in *N*. *crassa* (histidine supplementation in **S15 Fig**). The presence of multiple IAA biosynthesis pathways in microorganisms has been previously reported. For example, *Erwinia herbicola pv*. *gypsophilae* has both IPA and IAM pathways [45], wherein the IPA pathway is involved in epiphytic fitness while the IAM pathway is responsible for pathogenicity. Our study showed that tryptophan-supplemented cultures produce many indolic compounds other than IAM over time. In contrast, IAM supplementation resulted only in IAA production as the major indolic compound, along with very few other unidentified compounds. The IAM pathway has been shown to be present in the filamentous fungi *Fusarium* [14] and in *Colletotrichum gloeosporioides f*. *sp*. *Aeschynomene* [5]. In our study, we did not detect any trace of IAM produced by *N*. *crassa* strain(s) by TLC and HPLC analyses. However, we found that IAM supplementation in the *N*. *crassa* culture medium also led to IAA production. These results clearly suggest that tryptophan is used as a primary source for IAA production in *N*. *crassa* and that IAA synthesis proceeds through the IPA pathway to produce intermediate indolic compounds such as ILA and TOL, which are side products of the IPA pathway. The IAM pathway in *N*. *crassa* is activated only if IAM is supplemented in the medium; in its absence, no trace of IAM was detected in the metabolite analysis, which further suggests that the IAM pathway in *N*. *crassa* is a reserved and very specific secondary pathway for IAA production.

Tryptamine (TAM) supplementation produced a minute amount of IAA production when supplemented at a relatively higher concentration (**Fig 8B**), suggesting that although tryptamine supplementation leads to IAA production, the TAM pathway itself is inefficient and requires an additional secondary IAA production pathway in *N*. *crassa*. We also used single, double and triple knock-out strains encompassing other IAA biosynthetic routes. We studied IAA production using knock-out strains for IAA-biosynthetic genes from the IPA, YUC, IAM and TAM pathways. Our findings revealed that only those genes present in the IPA pathway were active and appeared to be produce IAA most efficiently in *N*. *crassa*.

The *Δcbs-3*;*Δahd-2* double knock-out strain produces fewer conidiospores, which is a significant finding because it suggests a biological function of auxin in the fungus. We conclude that aberrations in IAA-producing genes have a direct effect on conidiation as well as overall fungal growth.

## Materials and methods

### Identification of IAA-producing genes in *N*. *crassa*

Homologous protein sequences of tryptophan aminotransferase (ARO-8), indole-3-pyruvate decarboxylase (CFP), indole-3-acetaldehyde (IAAld) dehydrogenases and flavin monooxygenase (MOX-2) from different fungal species were obtained by BLAST [46] analysis and used in both bioinformatics as well as in experimental studies. First, *Neurospora crassa* protein sequences for the enzymes mentioned above were determined using the Broad Institute database (http://www.broadinstitute.org) and FungiDB (http://fungidb.org/fungidb/). Then, homologous sequences in other species were checked and retrieved by bidirectional BLAST using the Broad Institute database, FungiDB and the National Centre for Biotechnology Information (NCBI) [47, 48]. Only full-length sequences of the above-mentioned enzymes from different fungal species were considered for further analysis. Sequences were aligned using MUltiple Sequence Comparison by Log-Expectation (MUSCLE) [49, 50], and the alignments were visualized, edited, and presented using GeneDoc [51]. In a similar way, homologous sequences of tryptophan-2-monooxygenase (LAO-2), indole-3-acetamide hydrolase (NAA-2), aromatic-L-amino acid decarboxylase (*aad-1*) and glutamate decarboxylase (*gdc-3*) were obtained only for *N*. *crassa* and were used only for experimentation along with other genes. Throughout this study, we used the names of the genes and their corresponding enzymes per *N*. *crassa* conventions. However, we also found that some genes were annotated differently in different databases. For this reason, a description of the gene identity with its annotations is presented in **S1 Table,** showing annotations for both *N*. *crassa* as well as the general nomenclature for enzymes.

For the structural analysis, we implemented two independent methods to establish the consistency and reliability of each method, as well as the structure. We used the *Ab initio* method from the I-TASSER (http://zhanglab.ccmb.med.umich.edu/I-TASSER/) online-server [52] and the "SWISS-MODEL Repository portal" (http://www.expasy.org/) for homology modeling [53]. For the *Ab initio* method, corresponding enzyme sequences from *N*. *crassa* were submitted to the I-TASSER server, and the best model was chosen based on the C-scores. To choose a proper homologous structural template, individual enzymes were analyzed using BLAST against the PDB database (www.rcsb.org) prior to homology modeling. Suitable models were chosen for homology modeling based on the BLAST scores. We then predicted and cross-checked the cofactor binding properties in our predicted models by assessing the "structure-based function prediction" using the COFACTOR online server (http://zhanglab.ccmb.med.umich.edu/COFACTOR/) [38] for ARO-8, CFP, CBS-3 and MOX-2. COFACTOR makes predictions about the ligands and their binding sites on the structures. The reliability of ligand binding is defined by the BS-score. A score >1 reflects significant ligand binding in the structure. Ligand-binding sites present on both *N*. *crassa* enzymes and template structures (those used for homology modeling) were identified by alignments with known proteins. Finally, I-TASSER-predicted enzyme structures were aligned with template structures from the PDB. This structural alignment helps to obtain information about key residues inside enzymatic catalytic sites.

### Structure optimization

All the predicted structures generated by I-TASSER or SWISS-MODEL were evaluated in the PDBsum database using PROCHECK analysis [52]. As two independent approaches (*Ab initio* and homology modeling) were used for structure prediction, we further checked the consistency and reliability of the models by structural alignment of the same enzyme predicted by different methods.

### Molecular visualization

All the molecular visualization and editing was performed using PyMol (The PyMOL Molecular Graphics System, Version 1.5.0.4 Schrödinger, LLC.) and YASARA (http://www.yasara.org/). Images were processed using POV-Ray (http://www.povray.org/).

### Fungal strains and culture media

All the fungal strains, including wild type and single knock-out strains (**S1 Table**), were purchased from the fungal genetics stock center (FGSC) (http://www.fgsc.net/). All the strains are viable without any special supplementation. Single knock-out strains carry a *hph* (hygromycin B phosphotransferase) cassette in place of the ORF of the targeted genes [34]. Unless otherwise mentioned, the strains are always maintained and/or cultured on Vogel’s minimal medium (VMM) containing 2 ml of 50× Vogel’s salts (150 g Na_3_ citrate, 250 g of KH_2_PO_4_, 100 g of NH_4_NO_3_, 10 g of MgSO_4_, 5 g of CaCl_2_, 5 ml of trace element solution, 25 mg of biotin for a total volume of 1000 ml), and 2 g of sucrose, (2 g agar for solid media) for a total volume of 100 ml. Per the requirements, strains with two different mating types were crossed on Westergaard crossing medium containing 25 ml 4× Westergaard solution (4 g of KNO_3_, 4 g of KH_2_PO_4_, 4.1 g of MgSO_4_, 0.8 g of CaCl_2_, 0.4 g of NaCl, 400 μl of trace element solution, 10 mg of biotin for a total volume of 1000 ml), 2 g of sucrose, adjusted to pH 6.5, and 2 g of agar for a total volume of 100 ml. After successful crossing, ascospores were collected and spread on sorbose-glucose-fructose medium (20× SGF) (20 g of sorbose, 0.5 g of glucose, 0.5 g of fructose for a total volume of 100 ml) plates (for each 100 ml of total medium, 2 ml of 50× Vogel’s salts and 2 g of agarose were made up to 95 ml and autoclaved at 121°C for 20 minutes followed by the addition of 5 ml of 20× SGF). For DNA isolation, fungi were grown in liquid VMM medium, and for indole quantification and testing, liquid VMM medium was supplemented either with L-tryptophan (Sigma-Aldrich) or other supplementation as needed. All liquid cultures for indolic compound testing were performed in 50-ml flasks and incubated at 28°C on a rotary shaker.

### Setting for crosses, isolation of ascospores and propagation

Crosses were set up on plates containing Westergaard crossing medium [54]. The strains were allowed to grow at 25°C. After ~14 days of setting up the cross, the ascospores were collected. The ascospores were activated at 60°C for 45 minutes [55] and spread on SGF-agar plates. Next, one to two isolated colonies were transferred to VMM slant tubes and maintained at 25°C for a few days until they grew well and produced conidiospores.

### Estimation of the conidial number from different strains

Strains were inoculated in a 150–200 ml Erlenmeyer flask with 50 ml of VMM-agar medium and maintained for 10–21 days at 25°C in a fungal growth chamber. Next, 40 ml of 1 M sorbitol + 0.1 ml of Tween® 20 solution was poured carefully into the flask, which was then sealed with Parafilm and shaken well to remove all the spores from the solution. The mixture of spores, sorbitol and Tween® was then poured onto gauze and passed through a funnel for filtering, collected in 50-ml tubes and centrifuged at 4,000 rpm for 5 minutes. The spores were re-suspended and centrifuged with 50 ml of 1 M sorbitol only. This step was performed twice. Finally, the conidia were collected in a 1.5-ml tube with 500 μl of 1 M sorbitol, diluted at a ratio of 1:100 and counted using a hemocytometer. The formula applied for counting conidia is as follows: n (number of spores in large squares) / 4 = K ×10^8^ spores/ml. All counting was performed in triplicate and repeated several times.

### DNA isolation, primer designing and confirmation of the knockout strains by PCR

DNA isolation for screening purposes in search of positive knock-out strains was performed using the Quick-DNA™ Fungal/Bacterial Miniprep Kit (Zymo Research, USA), and the DNA concentration was measured in NanoDrop 2000 Spectrophotometer (Thermo Scientific, PEQLAB). As all single knock-out strains had a *hph-*cassette in the position of ORF of the targeted gene, all the subsequent knock-out strains (double, triple, etc.) also had the *hph-*cassette in the targeted positions. A successful *hph-*cassette exchange with the targeted gene was confirmed in two steps: first, by checking for the middle portion of the ORF by designing primers specific for the ORF of the targeted gene, and second, by designing primers such that the forward primer is always in the 5’ UTR of the targeted gene and the reverse primer was in the *hph-*cassette. Therefore, the reverse primer (5’ ATCCACTTAACGTTACTGAAATCTCCAAC 3’) was always same for all knock-out strains. A detailed list of primers is provided in **S2 Table** in the supplementary information. Each double or triple knock-out strain was first checked on an agarose gel after PCR, and then the DNA band from the 5’ UTR along with *hph-*cassette of the respective gene was sequenced (Sanger sequencing).

### Extraction of indolic compounds and analysis

#### Estimation of IAA and total indoles

IAA and other indole derivatives were estimated by the Salkowski method [56, 57]. Samples were filtered using cheesecloth, and the supernatant was collected. One milliliter of supernatant was mixed with 1 ml of Salkowski reagent (1.015 g of FeCl_3_. 6H_2_O, 150 ml of H_2_SO_4_, 250 ml of ddH_2_O) in a light-protected tube. The pH was adjusted to 2.8 with 25% HCl. The tubes were incubated for 30 minutes at 30°C in the dark for color development, and then the absorbance at OD_540_ was determined. The regression line and correlation coefficient (r2 >0.998) were determined using commercially available IAA (Sigma-Aldrich) standards prepared from serial dilutions of 100 mM stock solutions. All experiments were conducted with three replicates and were repeated at least three times.

#### Feeding test for IAA production with indole derivatives and other supplements

For the feeding tests, conidiospores were germinated in 50 ml of VMM medium with various supplements. Fungal indole production was checked by tryptophan supplementation at a final concentration of 1.96 mM unless otherwise mentioned. The IAA final concentration was set to 10 μM in the culture medium because this concentration has been reported to be the optimized one for maximum conidial germination [27]. Tryptamine (TAM), indole-3-acetamide (IAM) and tyrosine were used at different concentrations according to the needs of the experiments. A fixed concentration of indole-3-lactic acid (ILA) and tryptophol (TOL) was used in a final volume 2.5 mM each. The culture was induced in the dark at 28°C by shaking at 150 rpm. Negative controls were mock-inoculated. To assess the ability to produce IAA by the mentioned indoles, a feeding experiment was performed coupled to a time course assay. In the time course assay, wild type and different knock-out *N*. *crassa* strains were supplemented with different indolic compounds, and IAA production was checked every 12 or 24 hours for a total length of 96 or 72 hours, respectively, according to the experimental requirements.

#### TLC analysis

Fungal cultures were filtered through cheesecloth and collected in a tube. The pH was adjusted to 2.8 and mixed with equal volume of ethyl acetate (1:1 v/v). The aliquots were extracted by shaking at 200 rpm for 3 hours in the dark. After phase separation, 1.5 ml of the ethyl acetate fraction was removed to a light protected tube, and ethyl acetate was evaporated in a UNIVAPO 100H (UNIEQUIP, Planegg, Germany) rotating vacuum. The solid residue was dissolved in 60 μl of ethyl acetate and loaded onto silica gel 60 F254 plates (Merck, Darmstadt, Germany). The TLC plates were run for 1–1.5 hours with a buffer of chloroform: methanol: water (84: 14: 1), and color was developed by spraying a mixture of van Urk and Salkowski reagents at a ratio of 1: 3 [57] and subsequent incubation at 90°C for 10 min. The indole derivatives were identified by the retention factor (*R*_*f*_) and colors of the bands by loading 2 μl of 20 mM standard compounds on the plate.

#### HPLC analysis

HPLC analysis was performed in a 10 A Model HPLC (Shimadzu, Japan) equipped with a NUCLEODUR^®^ 100–5 C18 reverse column (250 mm length × 4 mm internal diameter), RF-10A XL fluorescence detector at a 280-nm wavelength. Following extraction with ethyl acetate, the dry pallet was dissolved in 60 μl of buffer containing 25% methanol and 1% acetic acid, pH 4.5. The same buffer was used to dilute the sample further at a ratio of 99:1 (buffer: sample). Indoles were separated on a 20–100% (v/v) MeOH- water gradient as solvent B and 2.5% acetic acid pH 3.8 set with 4 M KOH as solvent A over 45 minutes with a flow rate of 1 ml/min. Under these conditions, all the standard indolic compounds were detected within the first 30 minutes. IAA was detected and confirmed by the retention time and co-migration (spiking with an authentic standard). Including IAA, all other indolic compounds were detected, and integration of the different peaks was conducted according to the standards. Excluding tryptophan, other indolic compound standard stocks were prepared in methanol. The standard tryptophan stock was prepared in HPLC grade water. In each HPLC run, a standard mixture of different indoles was used at a 2 mM concentration each, diluted in sample-dissolving buffer. Standard curves for different indolic compounds were generated using three different concentration (5 μM, 2 μM and 1 μM) with four different injection volumes (2 μl, 6 μl, 10 μl and 15 μl) of each concentration. Thus, for each standard 12 (twelve), data points were obtained for the calibration curves. Calibration curves were plotted as the injected analyte mass vs peak area. The curves were then fitted, and a linear regression equation as well as coefficient (r^2^) were calculated. All the experiments were performed at least with three replicates and repeated several times.

## Supporting information

S1 Fig Multiple sequence alignment of tryptophan aminotransferase homologs from 26 different fungal species. Residues with 100% identity are shown in yellow font on a black background, residues with 75% identity are shown in black font on a red background, and residues with 50% identity are shown in black font on a green background. PLP binding residues are denoted by a pink star on top.
(PDF)

S2 Fig Sequence alignment of pyruvate decarboxylase homologs from 31 fungal species. The black background with yellow font, red background with black font and green background with black fonts represent 100%, 75% and 50% identity of the residues, respectively. TPP binding residues are denoted by pink stars on top of the alignment.
(PDF)

S3 Fig Multiple sequence alignment of aldehyde dehydrogenase homologs from 25 fungal species. Amino acid residues with black background yellow font represent 100% sequence identity; 75% identity is shown in black font on a red background, and 50% sequence identity is shown in black font on a green background. NAD serves as cofactor for the enzyme aldehyde dehydrogenase. NAD binding residues are denoted by a pink star the top of the residues.
(PDF)

S4 Fig Multiple sequence alignment of homologous MOX-2 from 33 different fungal species. MOX-2 or flavin monooxygenase is known to bind FAD through its nucleotide binding motif (GXGXXG). The GXGXXG motif is indicated by a bar, and conserved residues are highlighted with pink stars. “X” represents any amino acid. Residues in yellow font on a black background are 100% identical, and residues in black font on a red background are 75% identical. (the MOX-2 homolog in *U*. *mydis* was not found using the same BLAST search with the same template used for the other homologs.)
(PDF)

S5 Fig Sequence alignment of homologous pyruvate decarboxylase from different fungal species. A black background with yellow font, red background with black font and green background with black font represents 100%, 75% and 50% identity of the residues, respectively. TPP binding residues are denoted by pink stars. A blue discontinuous symbol shows a discontinuity in sequence.
(TIF)

S6 Fig Multiple sequence alignment of aldehyde dehydrogenase homologs from seven different fungal species. Amino acid residues in yellow font on a black background represent 100% sequence identity, in black font on a red background represent 75% identity and in black font on a green background represent 50% sequence identity. NAD serves as cofactor for the enzyme aldehyde dehydrogenase. NAD binding residues are indicated by a pink star on top of the residues.
(TIF)

S7 Fig Multiple sequence alignment of homologous MOX-2 from different fungal species. MOX-2 or flavin monooxygenase is known to bind to FAD through its nucleotide binding motif (GXGXXG). GXGXXG motif is shown as a bar, and conserved residues are highlighted by pink stars. Residues in yellow font on a black background are 100% identical and residues, and residues in black font on a red background are 75% identical.
(TIF)

S8 Fig Structural alignment and characterization of ligand binding sites of predicted *N*. *crassa* enzymes. Predicted structures and known templates are shown in blue and gray, respectively. Residues present on predicted enzymes and templates are marked with orange and green, respectively. All structures are rendered as a ribbon. Key amino acid residues involved in ligand binding are rendered as a stick model. Residues those are at close vicinity are only highlighted. Ligands are shown as a ball and stick model. (a) Overall structural alignment of the predicted CBS-3 structure with the known enzyme structure (1NZX). NAD is bound inside enzymatic catalytic site. (b) Structural insight of the ligand binding site of both the predicted and 1NZX known structure. (c) Structural alignment of the predicted structure of flavin monooxygenase homolog from *N*. *crassa* with the known flavin monooxygenase structure (1VQW). (d) Characterization of the ligand binding site of the predicted enzyme using the 1VQW structure as a template.
(TIF)

S9 Fig HPLC analysis of the metabolites produced by different *N*. *crassa* strains without tryptophan supplementation. Lower panel of the figure with numbers denotes the standard peaks of the various indolic compounds. Except indole-3-lactic acid (ILA) produced by *Δcfp* strain, no other indolic compounds comparable to the standard peaks were found within the time window set for the analysis. Genes that were analyzed from different pathways are as follows: *aro-8aro-8aro-8*, *cfp*, *cbs-3*, *mox-2*, *lao-2* (tryptophan-2-monooxygenase) and *naa-2* (indole-3-acetamide hydrolase). *lao-2* and *naa-2* are involved in IAM pathway of IAA biosynthesis. Standard peaks are: lane **1—**tryptophan, lane **2—**indole-3-lactic acid, lane **3—**tryptamine, lane **4**—indole-3-acetamide, lane **5—i**ndole-3-acetic acid, lane **6**—tryptophol, lane **7** -indole-3-acetonitrile.
(TIF)

S10 Fig Feeding test using tryptophan at higher concentrations. Different tryptophan concentrations ranging from 2 mM to 12.5 μM were used.
(PDF)

S11 Fig An example of an agarose gel image after electrophoresis with the PCR products confirming the two double knock-out strains. Two samples, namely, S1 and S8, were confirmed. S1 is a double knock-out strain for genes *lao-2* and *naa-2*, and S8 is a double knock-out strain for genes *lao-2* and *aro-8*. Lane 1 and 2 were loaded with the PCR products of 5’ UTR amplifications of *lao-2* and *naa-2* ORFs, respectively, along with the *hph* cassette. Lane 3 and 4 were loaded with the PCR products of mid-part of *lao-2* and *naa-2* gene ORFs respectively. Lane 5 and 6 were loaded with the PCR products of 5’ UTR amplifications of *lao-2* and *aro-8* ORFs, respectively, along with the *hph* cassette. Lane 7 and 8 were loaded with the PCR products of mid-part of *lao-2* and *aro-8* ORFs respectively. Lane 9, 10 and 11 were loaded with the PCR products of mid-part of *lao-2*, *naa-2* and *aro-8* ORFs, respectively, from wild type strain (wt). M—DNA marker.
(TIF)

S12 Fig HPLC analysis of the metabolites produced by different knock-out *N*. *crassa* strains supplemented with 1.96 mM tryptophan in 72 hours. Graphs with different colors represent different knock-out strains. A specific graph for a particular knock-out strain is mentioned in the figure with color bars. Standard peaks are ILA: indole-3-lactic acid, IAA: indole-3-acetic acid and TOL: tryptophol.
(TIF)

S13 Fig Indolic compounds including IAA production by wild type and mutant *N*. *crassa* with tryptophan supplementation in 12-hour intervals for a total of 96 hours. Changes in IAA and indolic compounds metabolism over time in wild type and mutant N. crassa supplemented with 1.96 mM tryptophan. (A) Wild type N. crassa strain was grown with tryptophan supplementation, and samples were collected in every 12 hours for a total of 96 hours. The following standards were used: tryptophol (TOL), tryptamine (TAM), indole-3-acetamide (IAM), indole-3-acetic acid (IAA), tryptophan (Trp), indole-3-pyruvic acid (IPA) and indole-3-lactic acid (ILA). (B) Δcfp knock-out strain was grown with tryptophan supplementation. At every 12-hour interval, samples were collected. Black arrow marks indicate ILA spots. The following standards were used: tryptophol (TOL), indole-3-acetamide (IAM), indole-3-acetic acid (IAA), tryptophan (Trp), indole-3-pyruvic acid (IPA) and indole-3-lactic acid (ILA). (C) The Δcbs-3Δahd-2 double knock-out strain was grown with tryptophan supplementation. At every 12-hour interval, samples were collected and analyzed. Black arrow marks indicate tryptophan spots. The following standards were used: tryptophol (TOL), indole-3-acetamide (IAM), indole-3-acetic acid (IAA), tryptophan (Trp), indole-3-pyruvic acid (IPA), and indole-3-lactic acid (ILA). (D) HPLC analysis of 48 hours old wild type and Δcbs-3;Δahd-2 double knock-out N. crassa culture supplemented with tryptophan. After 48 hours of inoculation Δcbs-3;Δahd-2 double knock-out strain shows a peak for tryptophan while corresponding peak from wild type strain has not been observed.
(TIF)

S14 Fig Feeding effect of various indolic compounds on *N*. *crassa*. Different indolic compounds were supplemented to wild type N. crassa culture media for checking their role in IAA production over time. tryptophan (Trp), tryptamine (TAM), indole-3-acetamide (IAM) and indole-3-acetic acid (IAA) were used for the assay. Samples were collected in 24-hour intervals. The numbers below the lanes represent the time in hours. Following standards were used: tryptamine (TAM), indole-3-acetamide (IAM), indole-3-acetic acid (IAA), tryptophan (Trp), and indole-3-pyruvic acid (IPA).
(TIF)

S15 Fig Effect of histidine supplementation on *N*. *crassa* in indole production. Fungal culture was supplemented with 2 mM histidine. Lane 1: Fungal culture was supplemented with 1.96 mM of tryptophan. Lane 2: Fungal culture was supplemented with 1 mM of tryptophan. Lane 3: Fungal culture was supplemented with 2 mM of histidine. IAA: indole-3-acetic acid standard.
(TIF)

S1 Table Detailed specification of *Neurospora crassa* strains. (PDF)

S2 Table Description of the primers used for PCR. (PDF)
