# Supplementary material for: Characterization of indole-3-pyruvic acid pathway-mediated biosynthesis of auxin in Neurospora crassa
Source: PLoS One. 2018 Feb 8;13(2):e0192293. doi: 10.1371/journal.pone.0192293 (PMC5805262; doi:10.1371/journal.pone.0192293)
Supplement: S1 Fig — Residues with 100% identity are shown in yellow font on a black background, residues with 75% identity are shown in black font on a red background, and residues with 50% identity are shown in black font on a green background. PLP binding residues are denoted by a pink star on top. (PDF) [file pone.0192293.s001.pdf]

```
N.crassa          : --MGEALINGSGAVSNVSALETLGPAAKLTDADPSEIPPASGPGR-ENAVTISD-ILERRA :  57
U.maydis          : -------------------------------------------------------MTPSAQ :   6
A.macrogynus      : ---------------------------------------------MFAQAVASRGRRVAVA :  16
A.nidulans        : ------------------------------------------------------------- :   -
B.cinerea         : ----------------------MAPPTAIDIEGVVDTESIVLP-----DPLTVNGVSARRA :  34
B.dendrobatidis   : --------------------------------MTIASLLLRPVKHCKAILSSAAISSSTMN :  29
C.albicans        : ------------------------------------------------------------- :   -
C.guilliermondii  : ------------------------------------------------------------- :   -
C.immitis         : ----------------------MSPHAAQDAAVQGAVDITKET---PEPLTVAN-VPARRA :  35
C.neoformans      : ------------------------------------------------------------- :   -
C.tropicalis      : ------------------------------------------------------------- :   -
F.graminearum     : MLSAVRTARLRSLIKHSRTIHSSSKHLSSTEAAPAARPAHYDT---SQKLSAIDAVKERRL :  58
F.oxysporum       : ------------------------------------------------------------- :   -
M.oryzae          : ------------------------------------------------------------- :   -
P.brasiliensis    : ----------------------MSPPGAVDPQIINNAD----P---PEPLTVAD-VPARRA :  31
P.nodorum         : ----------------------MAPPSAIEVSAETDTSGITIPNPLTAPITSND-IFGRRK :  38
P.triticina       : ------------------------------------------------------------- :   -
R.oryzae          : ------------------------------------------------------------- :   -
S.cryophilus      : ------------------------------------------------------------- :   -
S.japonicus       : ------------------------------------------------------------- :   -
S.pombe           : ------------------------------------------------------------- :   -
S.punctatus       : ------------------------------------------------------------- :   -
T.equinum         : --------------------------------------------------------MHLEF :   5
T.rubrum          : ----------------------MAPHAAAGAGTAGDAAQKAVV---EGTLTVAN-VAANRT :  35
U.reesii          : ----------------------------------------------------------MAP :   3
V.dahliae         : -MSAIACQTAPVGLISRRSFHAKPKLNSAGEAAPVQQDAVLEN---KQPISVFDSIRERRA :  57


N.crassa          : KAGRLIAPTASYADSDMFKGPQ-VGKPKSKSMN-HHLSAESLSRHPCALKQA------ARH : 110
U.maydis          : VANVDPNPYFGDKVGDLASTSETYPKASRVDPA-RFLSQVSHDRPASAIRSL------FPA :  60
A.macrogynus      : VPAPAARTLLGIRTMTTNNKTTTPIAKPIQDYT-KYLTAVSLARQPSAIRAL------QPI :  70
A.nidulans        : ----------------------MASLSPPLDLS-HHFSLTTKRRAPSAVKDF------YKY :  32
B.cinerea         : KAGKFVAGVAAF-----------TTSDHFKNPA-DRISLESRSRKPSNLKGA------AKY :  77
B.dendrobatidis   : SFSSVASSEKSDSSSLSVSMQLATAKKPITDYT-EYFSPRAAARRPSAIRDL------ATL :  83
C.albicans        : ----------------------------MSDPT-HLISKRAAGRTSVHFTNAPSDKPPANF :  32
C.guilliermondii  : ---------------------------MSEFVHGHLLSKRALARKSSHFAAI-DKSPPKGF :  33
C.immitis         : KT-TMPTGVAASCHSDMFKSLACATKPKAKRWD-KYLSVESKSRKASTLKQA------AEY :  88
C.neoformans      : -------------------MPSVAAVPVKLDYQ-SFLSQEAKGRIRSQLRDL------RYF :  35
C.tropicalis      : ---------------------------MPTDPS-HLISKRAASRSSSHFTNAPAGEVPIGF :  33
F.graminearum     : KAGKLVAGVAAASDSDMFKGPT-TGLPKSKRWD-NHLSHESKVREPCTLKQA------ARH : 111
F.oxysporum       : ----MAPSATDATAQASPAATALQALPPPKDLS-HLFSKVTRNRKPSNIKAF------YKF :  50
M.oryzae          : ------MQLPTRPLSRRQDNGEDDSRPLPRDLS-HYYSQTTKNRKPSQVKEF------YKF :  48
P.brasiliensis    : KT-KVPTGVAANSESDMFKSAACFTKPKAKRFE-HLLSAESKSRKGSSLKQA------AKY :  84
P.nodorum         : KALQSQWGIAAASDTANFRQKSYENKPKAKRWD-HILSQEALIRKGNSLKEA------AKF :  92
P.triticina       : -------------------------MVPAVDYDGKFISRAGKRWQPSGIRGL------FPL :  30
R.oryzae          : -----------------------------MDYS-KHISEASKARNPSAIRAL------MPY :  25
S.cryophilus      : ------------------------MSIQPKDLS-HHLSVESASRRPSPLKAI------AGS :  30
S.japonicus       : -----------------------MVAYQPKDLS-HHLSLESSARRPSPLKAA------ALS :  31
S.pombe           : ------------------------MDNLKKKYQ-HHLSLESASREYGPFQML------GRI :  30
S.punctatus       : ---------------------MTSEASNPDDYS-RFFTVRSAARKPSAIRAL------QPL :  33
T.equinum         : EKAHDVLHEDVRDAVNIRQAHFSAPKRLANRWD-HRLSDESLSQGASPLKNS------VKT :  59
T.rubrum          : RC-ALPKGVAAFSDSSMFKSAASASQPKAKRWD-RYLTEESKLRKPSTLKQV------ALY :  88
U.reesii          : MPTEPPLPGSHDSDDLNRRRTAEPMLRPPHDFS-RYYSCATRRRVASSVKDF------YKY :  57
V.dahliae         : KAGKLVAGVAAVSDSDMFKAPS-HDKPEAKRWD-HHLSHEAAVRKPCTLKQA------ARF : 110


N.crassa          : LKNPNI-----ISLGGGLPCAEYFPIESITLKVP-------------------TPPDFSEA : 147
U.maydis          : ELIPGM-----LSLLAGKPNPDTFPFESLSLNL---------------------------- :  88
A.macrogynus      : MAMPGM-----ISLAGGLPNPATFPIANVELTL---------------------------- :  98
A.nidulans        : FMIPGI-----ANLAGGLPNASYFPYDTLEASAA-------------------HPQRFPTI :  69
B.cinerea         : L-GPGM-----ISLGGGLPCPDYFPIEEISIKVP-------------------TVPHFSEA : 113
B.dendrobatidis   : VNVPGM-----ISLGGGNPHPSTFPFASIQYTL---------------------------- : 111
C.albicans        : KSHEKP-----LALSYGMPNHGFFPIDSIDVNLVDYPFQKITTPSTTSSTAEEEPPSSSLN :  88
C.guilliermondii  : KPADKN-----LILSWGTPNDGFFPIESIDLQLVDYPFE----------KSLSMSMTNASL :  79
C.immitis         : LKNPGL-----ISLGGGLPSPEYFPIDEISIKVP-------------------VAPNFSEE : 125
C.neoformans      : QAIPGM-----ISFGGGLPHPSTWPVNAMVLSVPFAKKS-------------IFIPGYEST :  78
```

```
C.tropicalis        : KPHPNP-----LLLSYGMPNHGFFPIDSIDVNIVDYPFQ--------TTLDHAKPNGSSTS :  81
F.graminearum       : MKKPGL-----ISLGGGLPSSEVFPFAELGFKVP------------------VAPKFSEK : 148
F.oxysporum         : MQIPGI-----SNFAGGMPNVKYFPFDTLEAQIS------------------NADRWEPS :  87
M.oryzae            : FQIPGI-----VNFAGGLPNLRFFPFDTLEAQTA------------------KPERWTPS :  85
P.brasiliensis      : LDKPGL-----ISLGGGLPSCEYFPFESISIKIP------------------TPPHFSEE : 121
P.nodorum           : LATPGL-----ISLGGGLPSSEYFPFEELSMRVP------------------QVGHFSEA : 129
P.triticina         : ENRPGM-----ISMLAGKPNPATFPFESIAITL-------------------------- :  58
R.oryzae            : LNRKDM-----ISLGAGQPNPATFPFASLTLTL-------------------------- :  53
S.cryophilus        : VSKKGI---NVISLAGGLPNPQYFPFRKMGSEIP------------------SIGSWVRG :  69
S.japonicus         : KSKTGI---NIVSLAGGLPHSDYFPIRTLSTAV-----------------------YRPD :  65
S.pombe             : QSDSDI---KMLSFAGGEPNPSKFPIHKLSVSFP------------------EVNSWEKD :  69
S.punctatus         : LSIPGMFGLVQISLGGGNPNPTTFPFKELSFTL-------------------------- :  66
T.equinum           : ATTFVP-----IPLGVGRPASLFYPWQSMTMTG-------------------------- :  87
T.rubrum            : M-GPNV-----ISLGGGLPSQEYFPFHEISVKAP------------------SPRQFADP : 124
U.reesii            : FAIPGI-----HNLAGGLPHVSYFPFDSLEATVA------------------LPNRIPKD :  94
V.dahliae           : LKTPGL-----ISLGGGLPSSEYFPFSEITMRVP------------------TPPDFAEK : 147


N.crassa            : AT----SQSGAE-----------------------------VRIGKYDAT-DPSIQGT--- : 171
U.maydis            : ------KPEAEA------------------------------GPTQLNIQ-------G--- : 103
A.macrogynus        : -----------------------------------------KNGAKISMG-------D--- : 108
A.nidulans          : PGGKKAKGPQTS--------------------------------ERIVVPKESRSATS-KK :  97
B.cinerea           : QT----KESGTI-----------------------------LTAGKYDVS-EG--KGA--- : 135
B.dendrobatidis     : -------KSGEV-------------------------------------IDIP------T--- : 121
C.albicans          : GSENGHQTKTPP-------------------------SSIHTPQSTVHISRHTTDPKL--- : 121
C.guilliermondii    : ESLALSKTNGTV-------------------------ANDSSGPKKITINRRSEDEKL--- : 112
C.immitis           : AT----HESGQV-----------------------------VTAGKYDIR-EG--RSE--- : 147
C.neoformans        : DPTSL-HPLAPY-----------------------------TPPTKLSIATDP---FA--- : 103
C.tropicalis        : DKINDLKSSDSE-------------------------NNQHDSLHTIHIERIPKDPKL--- : 114
F.graminearum       : DT----EESGQT-----------------------------VTIGKYDVR-DR--GGT--- : 170
F.oxysporum         : PNYPDKVDPSTESLSGSVIGYSNSPDKNAPRNPDYNKTSSRISIPKILNEPDI--TKK--- : 143
M.oryzae            : ESTATNTDSRSN----------------------GDSRDRSESTHITVPLVAKETDPLRK : 123
P.brasiliensis      : ET----VKTGTV-----------------------------VTSAKYDIR-NG--KSD--- : 143
P.nodorum           : ET----KESGVL-----------------------------IKAGKHDLA-DG--KSI--- : 151
P.triticina         : ------KPSVAT----------------------------GEVPETLCLS-------G--- :  75
R.oryzae            : -----------------------------------------KTGEKIEID-------D--- :  63
S.cryophilus        : A-----ENEGKL----------------------------------SDFSVSMDSEDQDD--- :  90
S.japonicus         : GK----PENNNV---------------------------SKIPLQELSLGMEPTEDND--- :  92
S.pombe             : TN----KDATVS----------------------------------YELS-NNANEGS--- :  88
S.punctatus         : ------KDEAGG--------------------------------GEIHVE-------S--- :  79
T.equinum           : ------TKESNT-----------------------------RNPMVCNIG-----EAA--- : 105
T.rubrum            : AP----ANPECV-----------------------------FSSTKHDVV-DG--TSE--- : 146
U.reesii            : GFVPQPQPTASP-------------------------ASPPASQRVVVPKESSVDDGLRR : 129
V.dahliae           : DNLSAQSPTAQT-----------------------------ISIGKYDVR-DG--KSE--- : 173

                                *
N.crassa            : YDLAVALNYAQASGSAQLLRFVTEHTELVHN-----P--PY----SD--WRSCLTIGSTGA : 219
U.maydis            : EDLVSALQYGATSGIPKLVQWITELQVHMHKREAVTPGSKLDGVAGRTPWRVTTGNGSQDL : 164
A.macrogynus        : TMTRAALQYGPTPGHPDLVAFFEKLQARVHG-----T--PYANKQLE--RKILVGNGSQDL : 160
A.nidulans          : IDITTALQYGTSEGLPPLATFVNQFVREHLH-----PNIPYA---GG--PGTLLTTGATDG : 148
B.cinerea           : YDLSIALNYGQGSGSAQMLRWVTEHTEIVCN-----P--PY----AD--WGCSLTVGSTSA : 183
B.dendrobatidis     : EQVKQALQYSATPGLEKFVEWLKKFQVHEHGRD---P--------LE--FDVCIGNGSQDL : 169
C.albicans          : IDLARGLQYAAVEGHAPLLQFARDFIIRTHK-----P--NY----DD--WNVFITTGASDG : 169
C.guilliermondii    : IDIAHGLQYSEPKGHPQILQFTREFISKIHP-----P--KY----SD--WDTIVTTGASDG : 160
C.immitis           : YDLEIALNYGQSVGAAQLLRFVTEHTEIVHN-----P--PY----SD--WHCSLTAASTAS : 195
C.neoformans        : PDLARDLQYSSNFGLPYFVDWLTQHVKRIHD-----P--PY----PEDRWQVLATAGNTDA : 153
C.tropicalis        : MGLKSGLQYAAVEGHQQLLQFTKDFIQRTHN-----P--NY----DN--WTSFITNGASDG : 162
F.graminearum       : YDLSIACNYGQATGSPQMMRYLTEHTEIVYK-----P--PY----AD--WKVCQTIGSTGA : 218
F.oxysporum         : VDLATALQYGQVTGYPPLHSFIRQFTNQVLH-----PSVPYQ---GG--ADVILNNGGTDG : 194
M.oryzae            : IDLATALQYGMADGYPPLLSFVRQFTREHLH-----PDVPYR---SG--PEVVLTVGSTDG : 174
P.brasiliensis      : LDLEISLNYGQSNGAAPMMRFVTEHTEIVHN-----P--PY----SD--WQCSLTAGSTSS : 191
P.nodorum           : FDIATAFNYGQGAGSAQLLRWMTEHTELVHN-----P--PY----ED--WRCCMSIGSTSA : 199
P.triticina         : RELDAALQYGPTAGQEEFLNWVYQLQQRCHC-----RGTP-----EEEGWSCAIGAGSQEL : 126
R.oryzae            : QLFDRSLSYDLTSGQPLLNQWLTELQKIEHA-----P--P-----VD--FDVSIGSGSQDL : 110
S.cryophilus        : LPLSVALQYGKGSGCAQLTSFLKEHTQMIHS-----P--PY----AN--WDILLTTGNTQG : 138
S.japonicus         : FGLSQGLQYGQGFGALQLAAFVKEHTRIVHQ-----P--KY----EG--WDVMLTAGNTNA : 140
S.pombe             : LDLLGALQYGQCQGIPELVKFIKDHVGQIHM-----P--QY----KD--WDIKITNGNTIG : 136
S.punctatus         : GDLQKALQYSPTNGIPEFVAWLKELQTSEHS-----P--LY----DS--FDVCVGNGSQDV : 127
T.equinum           : FDLSSALNYADPSGSTELVACFRENTRLIHN-----P--PY----QD--WDTTLTCGSTSA : 153
T.rubrum            : FDLEVALNYGQSVGYAALVRWITEHVEIVHN-----P--PY----GD--WEITMAPGSTQG : 194
U.reesii            : IDLATALQYGAVDGYPPLRSFIRQFVRENLH-----PHVPYE---GG--PEVILTCGATDG : 180
```

```
V.dahliae        : YDLSIALNYGQSTGSAQMMRFVTEHTELVYN-----P--PY----AD--WKVCQTVGSTGA : 221

N.crassa         : LEQTLRMLCDGPGRRD--------SLLTEEYSFSTALETAGPL----DLKVVGVKM----- : 263
U.maydis         : LNKTFDALL---NPGD--------VILVESPAYTGILPSLVMI----KANIVPVTS----- : 205
A.macrogynus     : LAKAINALV---APGD--------TLLVESPCYVGTLAILKPMVKAQGVKLAEVPT----- : 205
A.nidulans       : FSKAIEAFTETWNPDR-DGIHLRQGILCEEFVYMNAIQTVKPR----GLNIVPVAI----- : 199
B.cinerea        : LEQTYRIFC---ERGD--------FVLSEEYTFASAVETAAPL----GVKFLGVKM----- : 224
B.dendrobatidis  : LTKAIDAFV---TDGG--------SVMMECPAYVGMIAYVRPT----GANIVEIPS----- : 210
C.albicans       : LNKAADVFL---DDGD--------VILVEEFTFSPFLRFSDNA----GAKAVPVKINFD-- : 213
C.guilliermondii : LNKVADALL---DPGD--------VILIEEFTFTPFLAHVANT----GGIPVPVRMNLDPT : 206
C.immitis        : WDMVLRLFC---NRGD--------YILTEEFTFSSAMETALPQ----GIRLAPVKM----- : 236
C.neoformans     : SDGVVRTLC---SPGD--------SMLLEEFAFPGSLTHYKSL----GINCVGVPM----- : 194
C.tropicalis     : LNKACDALL---DPGD--------VILVEEFTFSPFLRFSENS----GAIAISVRLNLN-- : 206
F.graminearum    : LEEALRMFCDK-DRGD--------SVLTEDFSFSTALETVGPL----GIKAFGVSI----- : 261
F.oxysporum      : FSKVLQLLVDPWYEGL-HPVVERPGMLCETFVFGNILTQARPL----GVNVVPVEI----- : 245
M.oryzae         : FAKTLELFVDPWSEGKGDDVRDRPGLLCERFAYGNVLTQARPK----GVQVVTVEA----- : 226
P.brasiliensis   : WDMALRMFC---ERGD--------YILAEEYTFATALEAASAL----GVRAAAVKM----- : 232
P.nodorum        : LDMALRMFA---RPGD--------VVLSEEYTFSAFVETARPM----GVRVCGIPL----- : 240
P.triticina      : MEKAFAAVC---DPED--------TILMETPVYSGTLGFLQPS----GRTLIEIES----- : 167
R.oryzae         : LTKALEMMI---NPGD--------AVMVEEPTYTGALAFLETM----PCTLAHIAT----- : 151
S.cryophilus     : LDMCLRMLM---NRGD--------SILVEKYTFPSALQAMRPM----GLNAVSVDM----- : 179
S.japonicus      : LDTCLRMLL---NRGE--------SLLVEEYTFPSALQTIEPM----GINCIPMPM----- : 181
S.pombe          : LEYCLRLLV---NRGD--------CILIEKYTYPAAITAMRPL----GVKFIPIDM----- : 177
S.punctatus      : LTKAFEMLL---AEGD--------NLLIESPAYVGSLAFLKPL----GCKFVEVPV----- : 168
T.equinum        : IDIVLRMLC---NRGD--------WILAEASTYTGTVMATKAH----GLNIQSIEM----- : 194
T.rubrum         : WDFVLRMLC---KPGD--------MILTEEYTFCSAMEGAAAQ----GVTPFPVKV----- : 235
U.reesii         : FSKTIEAFTNVWDENR-DWIRERQGLLCEEFAYMTAIQTARPR----GLNIVPVAV----- : 231
V.dahliae        : LEQALRMLCDR-DRGD--------SVLTEEYSFSTALETIGPL----GIKTFGVKM----- : 264

                                                                 *
N.crassa         : --DGQGLLPE---AMDELLSNWNPEEREG---RRKPHVLYTVPSGQNPTGTTQGLERRKQV : 316
U.maydis         : --DDQGMMSS---RLAEILANWETDPQTAS--LARPKCLYTTPTGANPAGTTASDERKRQI : 259
A.macrogynus     : --DAHGLIPD---ALDKILADWPAT-------SPKPRVLYTVPTGGNPTGNMAPIERRRAI : 254
A.nidulans       : --DDEGMLPYGPGGLAEVLGNWNHRK------GCRPHLMYTITIGQNPTGGTLSVERRKQI : 252
B.cinerea        : --DAEGLLPE---ALDEILSNWDEQAR-G---ARKPFVLYTVPSGQNPTGATQGIERRHAI : 276
B.dendrobatidis  : --DADGLNPD---AMEKTLANWPDV-------KTRPKILYTVPTAGNPTGVTTSLARKKRV : 259
C.albicans       : -NDSDGIDLT---QFVDLLENWEKHYP-N---LPKPKALYTIATGQNPTGFTQSLEFRKKI : 266
C.guilliermondii : PDKSNGLDID---YLTDLLENWETKKP-D---LKKPKALYSIPTGQNPTGLTQSLEFRKKV : 260
C.immitis        : --DEQGLLPS---SLDEVLTNWDASAR-G---ARKPFVLYTVPSGQNPTGATQDVERRKEV : 288
C.neoformans     : --DGEGIVPS---ALEKILAEWNEEAR-G---GPKPKAILIVPTCSNPSGITYPTPRKNEI : 246
C.tropicalis     : -KESDGLDLD---HLVGLLENWKEEHP-E---LPKPKALYTIATGHNPTGFTQSLEFRKKV : 259
F.graminearum    : --DEQGLIPE---AMDELLSNWDAKER-G---SRKPHLLYTVPSGQNPTGATQGTERRKAI : 313
F.oxysporum      : --DEEGMIVEGSGGLREVLENWNPQN------GRLPHFLYTVTMGDNPTSGVVGVQRRKEI : 298
M.oryzae         : --DSGGMLADGPGGLEDVLANWDDSK------GRRPHLLYTVTMGHNPTGIVLDVERRKAL : 279
P.brasiliensis   : --DDQGMLPE---SLDEVMTNWDAAAR-G---ARKPHLLYIVPSGQNPTGTTHGPERRKEI : 284
P.nodorum        : --DAEGLIPE---QLDNILSTWDVNVR-G---ARRPHLLYTVPTGQNPTGATQSAERRRAL : 292
P.triticina      : --DHHGISAV---ELERVLANWHSDPATSS--LKFPKVVYSIPTGSNPTGCSAPLERKRLI : 221
R.oryzae         : --DEFGIVPE---SLENMLAHWPESNPSGKKDQPRPHVLYTIPSGGNPTGISATYERKQAV : 207
S.cryophilus     : --DKNGMLPE---SLEEILNNWK-----G---GNKPRVLYTIPSGQNPTGSTLSVERKEKI : 227
S.japonicus      : --DLNGILPE---KLEELLSSWNTKAR-G---CPKPHVMYVIPSGQNPTGSTLSNERRARI : 233
S.pombe          : --DENGMLPE---SFEKVMETWDSSL------GARPHVLYTIPTGQNPTGSTLTLERRKKF : 227
S.punctatus      : --DKDGLDSD---KLEEILSGWKDV-------RTRPKVLYTVPVGGNPTGVSTSVERKRKI : 217
T.equinum        : --DEEGLLPV---DLDNKLRHWDTLR------SRKPFVLYTIPSGHNPTGVTQSTARKAAI : 244
T.rubrum         : --DAEGLLAT---SLDEILTNWDPAAHGG---ARKPIVLYTVPSGQNPTGATQSAERRREV : 288
U.reesii         : --DSQGMKVEGERGLADVLDNWDFSR------GQRPHLMYTVTIGQNPTGAVLPLERRRQI : 284
V.dahliae        : --DAEGLLPE---SMDELLTSWDEKAR-G---ARKPHVLYTVPSGQNPTGATQGPERRRAV : 316

                                *  *
N.crassa         : YEVARKHDLFVIEDEPYYYLQMPL---EESKETESEEET-------------VESFMSS- : 359
U.maydis         : LALARQYDFLVLEDDPYYYLH------FEGLDQDAVTRP--------------------- : 292
A.macrogynus     : YALAQKHDLVMLEDDPYYYLQFALPPTDAATQQLTPPAD-------QTTDVALGDRYASA- : 307
A.nidulans       : YAICQKYDVIIVEDDPYWNLQYPSAQELQARHRNSSVNPALSERNYNAGRKSSGYKFLDS- : 312
B.cinerea        : YKVAQKHDLYIIEDEPYYFLQMQP---YTGPDAPDVPPP------------ANNDEFLKA- : 321
```

```
B.dendrobatidis  : YEVARKYNVVIMEDDPYYFLQF-------------------------------GEKRD--- : 286
C.albicans       : YDLAVKYDFAIIEDDPYGYLTLPK---YEKPNIGGSGSG-----NNELKNDLEIDDYLKNH : 319
C.guilliermondii : YALAEKHDFIIIEDDPYGYLTLPP---YSKPEGVLKLDS-----------FLTVEEYLSNH : 307
C.immitis        : YKVAQKHDIIIVEDEPYYFLQMQP---YKGANAPQDPPP------------ANYNEFLKA- : 333
C.neoformans     : YAICRKWNLLIIEDDPYCYLQVRP----NGADTP--------------------------- : 276
C.tropicalis     : YALAEKYDFAIIEDDPYGYLTLPT---YTKPDLTKNPTR--------NKSDITVDDYLKNH : 309
F.graminearum    : YAVAQKHDIYIIEDEPYYFLQMQP---YTGRNQPDVPPP------------KTVDEFVSS- : 358
F.oxysporum      : YALASKYDIIIVEDDPYWYLQFPSAAIHEAKARGYAIPQ---VADSHKTVKSSGYDFIDS- : 355
M.oryzae         : YAICSKYDVLIVEDDPYWYMQFPSAAREEAKSRNLPPPQ-----VTSTYSRSTGYPFLDS- : 334
P.brasiliensis   : YKVAQKHDIFIVEDEPYYFLQMQP---YKGVDTPPDAPP------------ASYEAFIKS- : 329
P.nodorum        : YAVCQKHDIYIIEDEPYYFLQMQP---YTGPNAPVVPPP------------SSHDEFLNS- : 337
P.triticina      : LELVRKYDLLLFEDDAYYFLHFDR----------------------------------KN- : 247
R.oryzae         : YKICSKYDVIILEDDAYYYLQY-------------------------------GEKRT--- : 234
S.cryophilus     : YALAQQHDFIIIEDEPYYYLQMDE---YQGKPEAVDPKF-------------TNEEFVNN- : 271
S.japonicus      : YEICQAHDIIILEDEPYYFLQMDN---FDG-KIPEEQPT------------VSNETFLSQ- : 277
S.pombe          : LTLAKKYDIIIVEDEPYYFLQMEK---YDANWKPDKQAF-------------NISSFKKK- : 271
S.punctatus      : YEIAKRYDIIILEDDPYYYLQF-------------------------------GDKRA--- : 244
T.equinum        : YQVAERHDLLILEDDPYFFLRLNG---SFS-SLDINPPY------------SSFEKLRKS- : 288
T.rubrum         : YKVAQKHDIIVVEDEPYYFLQMEP---YTGLNTPTPPPP------------ATTDEFLKT- : 333
U.reesii         : YKLCQRYDIIIIEDDPYWHLQYPSAKQNALRQGQSDSSP---HTPFSVHDEPSGFPFLDS- : 341
V.dahliae        : YAVCQKHDVYIIEDEPYYFLQMQP---YAGKDAPVPPPP------------ASVDQFLAD- : 361

                                               *      *
N.crassa         : LIPSLLSLDVD-------GRVLRMDSFSKVVVPGSRVGWVTASENIIEQYLRHAEVCNQGA : 413
U.maydis         : RCRSYWSLEEEHRERWGTGRVIRFESFSKILAAGLRLGFATGPNEILDAVDANTAMSNLQP : 353
A.macrogynus     : LVPSYTSLDTD-------ARVIRFESLSKVISAGLRVGLCTGPAPLVDRIALDIQATSLQP : 361
A.nidulans       : LVPSYLSIDMD-------GRVVRLDTFSKTIAPGCRLGWITAQPAFIERLARIAESSTQAP : 366
B.cinerea        : LIPTYLSIDTD-------GRVMRMDSFSKVIAPGTRVGWITASEQIVERFIRSNENSNQNP : 375
B.dendrobatidis  : --PSYFSFDVD-------GRVLRFDSLSKVLSGGARIGWVSGPKMLVERIILHSMGSNLHP : 338
C.albicans       : LTPSYLELDTT-------GRVLRVETFSKLFAPGLRLGFIVGHKEVIDAVKNYSDVVNRGA : 373
C.guilliermondii : LVPSYLTLDTT-------GRVVRIETFSKLFAPGLRVGFVVGHKKVTEVIGQYAAVVTRAP : 361
C.immitis        : LIPSFLSMDVD-------GRVVRLESFSKVLSPGSRVGWLVASEQIVERFLRNAETCTQNP : 387
C.neoformans     : LIPSFLSLDVD-------GRVVRLDSFSKFIAPGSRCGWITGPKELVTAVMVKAEASSNGP : 330
C.tropicalis     : LTPSYLELDTT-------GRVIRVETFSKLFAPGLRLGFVIAHVKIIEAIKNYSEVINRGA : 363
F.graminearum    : LIPSLLSIDVD-------GRVMRMDSFSKVLVPGSRLGWITASEQIVERYIRHAEVASQGP : 412
F.oxysporum      : LVPSYLSIDVD-------GRVIRLDTFSKTVAPGSRLGYATAQPAIIERLTRIAETSTGQP : 409
M.oryzae         : LVPSFLSFDVD-------GRVVRLDTFSKTVAPGCRLGWLTAQPAIIERFVRITETSTQQP : 388
P.brasiliensis   : LIPSYLSIDVD-------GRVLRLESFSKVISPGSRMGWIVGTAEIVERYTRHSEVSTQNP : 383
P.nodorum        : LVPSYLSMDVD-------GRVMRLDSFSKVVSPGARIGWVTACAEIVEKYTKHADVSTQNP : 391
P.triticina      : RAPSYFALEKSEGGQ--TGRVVRFDSLSKIISSGIRLGFVTGPARILEIINLHTSNTNLQP : 306
R.oryzae         : --PSYLSLDVD-------GRVLRCDSMSKILSSGLRIGWVSGPQPLIERMNMHTMVTNLQP : 286
S.cryophilus     : LITSFLHIDVD-------GRVIRMDSLSKVVAPGSRVGWITAQPLFIERSLRYTETASQNA : 325
S.japonicus      : LITSFLSIDTE-------GRVIRLDSMSKVVAPGTRLGWLTGHPLFMERALRQNEVNIQSA : 331
S.pombe          : LIPSLLHLDTD-------GRVLRVDSFSKLIVPGLRLGWITGNSLFIDRITRYAEVCTESP : 325
S.punctatus      : --ATYFSMDAD-------KRVLRFDSLSKIFSAGMRIGWVTGPKPLVDRIVLHGQATMLHP : 296
T.equinum        : LPSSYLSLDKS-------GRVIRVDSTSKILAPGLRCGWLTASKQIVEIFENFAEVGPAPP : 342
T.rubrum         : LIPSYLSMDTD-------GRVIRLDSFSKVISPGSRVSFLVSSAQFTERMIRQAETSTQAP : 387
U.reesii         : LVKSYLSIDTA-------GRVVRLDTFSKIIAPGCRLGWLTAQPDIVERILRISETSTQQP : 395
V.dahliae        : LVPSLLSMDTD-------GRVLRMDSFSKVLVPGSRLGWITASAQMVERYIRHAEVANQGP : 415

N.crassa         : NGISQVILYKLL-----------------------DEQW-GHE-GYFK-WLMNLRQEYTKR : 448
U.maydis         : SGLAGVVAYTLL------------------------NYW-GIP-GFLR-HVDNVARYYAKR : 387
A.macrogynus     : TGLSQAVILALA-----------------------QHSWHGVPAGFLA-HAARVAAFYRSR : 398
A.nidulans       : SGFVQAMVAELILGQQVDDGSAD-----SAKSKDTERAW-KMD-GWVR-WLEGLRAGYEQR : 419
B.cinerea        : SGISQMVLYKLL-----------------------DESW-GHG-GYLQ-WLINLRLEYTKR : 410
B.dendrobatidis  : SGISQIMMYSLL------------------------EKW-GIE-GFLE-HNRKVAAFYKEK : 372
C.albicans       : SGLTQTIVNNVI-----------------------QENFKGVD-GWLE-WILKMRLNYSYR : 409
C.guilliermondii : SGLSQMVLNNVI-----------------------EQKYKGVY-GWLQ-WILKMRLTYTHR : 397
C.immitis        : SGMSQIILFKLL-----------------------DEAW-GHE-KYLQ-WLVHIRMAYTNR : 422
C.neoformans     : SGFAVASISAVI------------------------KAWGGHE-GLERDYLPHISDTYSKR : 366
C.tropicalis     : SGLTQTIVNNVI-----------------------QDNFKGVE-GWLE-WILKMRSNYAYR : 399
F.graminearum    : SGFSQVILYKLL-----------------------DETW-GHE-GYLR-WLMNLRLEYTKK : 447
F.oxysporum      : SGFVQALIAQAILGPHSS-TLETFSSL-PESEKPSFTGW-QLD-GWVR-WLEGLRGEYERR : 465
M.oryzae         : SGFVQSMIGELVMSSQPTETKTTFLGLRTHKDRTAFTGW-QMD-GWVR-WLEGLRGVYEDR : 446
P.brasiliensis   : SGMSQIILFKLL-----------------------DESW-GHA-GYFD-WLINLRMEYTAR : 418
P.nodorum        : SGMSQLILFKLL-----------------------EDHW-GHA-GYLD-WLIHIRMQYTKR : 426
P.triticina      : SSVTQAITGKLL------------------------CHW-GFD-RFEA-HCDQVSKFYSDK : 340
```

```
R.oryzae         : SGVSQVMAYQLL------------------------ANW-GHQ-GFFE-HVKRVADFYKEK : 320
S.cryophilus     : SGISQAMLHALF------------------------KTW-GHD-GYLA-WLKHIRYSYTER : 359
S.japonicus      : SGLSQVLLHGIL------------------------HTW-GQE-GYLE-WLKQIRFQYTRR : 365
S.pombe          : SGVSQVVLYAIL------------------------NRW-GQN-GFLE-WLQDLQNSYTMR : 359
S.punctatus      : SGLSQMLAYQLL------------------------KKW-GRD-GFLK-HTRSVAGFYEQK : 330
T.equinum        : SGPSQVMLYKLF-----------------------VESW-GQE-GFAN-WVNHLSGEYRSR : 377
T.rubrum         : SGMAQIILYKLL-----------------------DESW-GHE-GYLK-WLIHIRIEYTKR : 422
U.reesii         : SGFVQSMVAKLIMGDQADDMVKK-----PC--TEGSPGW-RVD-GWVR-WLEGLRGGYEKR : 446
V.dahliae        : SGFSQVMLWKLL-----------------------DETW-GHE-GYLK-WLIDLRMNYTRR : 450


N.crassa         : RDTMVTAAE--KFLP----------------------K-EVVSWTVPVAGMFYWLKIDHSQ : 484
U.maydis         : RDNFEAKAN--KVLG----------------------AAGVAQWVTPVAGMFLWLRLNLPP : 424
A.macrogynus     : RDALLKAMSAARLVE----------------------D-GLVEMSVPQAGMFLWLKL---- : 432
A.nidulans       : MQQMCSGLEEGKYIALHTRSDEFSLSGNEEDSWEVLEKVQMYDFAWPTGGMFVWVKVNYES : 480
B.cinerea        : RNVILAACE--KYLP----------------------K-DIVSWNPPSAGMFLWLSVDWHK : 446
B.dendrobatidis  : CDAFMSSAK--RHLD------------------------GIAEFVAPKAGMFVWMKL---- : 403
C.albicans       : KDLLLYSIFESQAYK----------------------K-GYVDVIDPKAGMFVTFKINLPK : 447
C.guilliermondii : RDVLVNAIEESDAYK----------------------K-NYVSIIDPRAGMFLSLVVNFPP : 435
C.immitis        : RNVMLEACE--KYLP----------------------T-SVASWHPPAAGMFHWIKVDWKK : 458
C.neoformans     : SNLMVSLIR--KYVP-----------------------VEAAECPDGSGGMFLWVRLRVES : 402
C.tropicalis     : KDLLLYSIFESEAYK----------------------K-GYVDVIDPKAGMFVTFLINLPP : 437
F.graminearum    : RDALLAACE--DHLP----------------------S-DLASWTPPVAGMFMWINIDYTK : 483
F.oxysporum      : MTRMCSILEENAFSL-RQSTSQNP-----DLDWDVISKIRLFDFDWPRGGMFVWVRVHFEK : 520
M.oryzae         : MNRVCRILDDGKYQL-KQGTPVVPGSHPLDADWGVITKKKLFEFDWPRGGMFVWLRVFFES : 506
P.brasiliensis   : RNTMLDACE--KYLP----------------------K-SIASWNPPAAGMFHWIKVDWKR : 454
P.nodorum        : RDVILQACT--KYLP----------------------T-EVMSWKPPMAGMFHWMQIDFKK : 462
P.triticina      : LEVVNAAAK---------------------------------------------------- : 349
R.oryzae         : RDDFVECLD--KHMT------------------------GRAVWTVPNAGMFVWLRL---- : 351
S.cryophilus     : RNALLYAMD--KHLP----------------------K-SICSYIAPEAGMFIWFEVDKSA : 395
S.japonicus      : RNALLLAMN--KYLP----------------------E-GLCSYIAPEAGMFIWFEVDRSR : 401
S.pombe          : RNALLLAAD--KHLP----------------------K-SVCKYHSPKAGLFLWVELDKNR : 395
S.punctatus      : RDLFLQSAS--KRLQ------------------------GVAEWTVPDAGMFVWLKL---- : 361
T.equinum        : RDIMIAACS--QHLP----------------------K-GLCTWTTPTHGMFLWITIDLLR : 413
T.rubrum         : RNCIMDACE--EFLP----------------------K-AVASCNPPTAGMFNWMRVDWKK : 458
U.reesii         : MQTMCTILEEGRFSIVQ---GDALSNDSNLDEWQVVDNVQMFDFDWPGAGMFVWVKFCLET : 504
V.dahliae        : RNMLLAACE--DHLP----------------------K-DIVSWTPPAAGMFLWLKVDHTK : 486


N.crassa         : HPGIQA-------------GKGILEVEEEIFNSCIARGVLVARGSWFR----AE-Q-DTEP : 526
U.maydis         : TGQ----------------GESEGDSFALISDKAKAAGVLAVPGVAFI----PD-----GS : 460
A.macrogynus     : --------------------KTVDDASALVKGYATDRKVLLLPGIEFL----PN--AQPGE : 467
A.nidulans       : HPLRQI------------YGPERLSKALWVHFTQKPQLCLLGPGTIFS----PT-DEVQAR : 524
B.cinerea        : HPAAKT--------------KPIADIEQEIFLSGIDQGVLICPGSWFT----AE-KDGFVP : 488
B.dendrobatidis  : --------------------IGIDDSTDLIKRKALEKKVLLVPGIEFL----PN-----AR : 435
C.albicans       : -------------------DVDVLQKMKLLLWKLISYGILVVPGYNMT----VD-LEFSKD : 484
C.guilliermondii : -------------------GTPIIEKIRLLNWKLLAFGVHAVPGINMA----TD-SEFSKE : 472
C.immitis        : HPLALA-------------GVSYDSIEEAIFKAAVGEGVLVSRGSWFF----AD-K-STEP : 500
C.neoformans     : HPQLST--------------LSPEEISDKVFHTLIGEKVMVAPSSYFKTPGGPVWSKDEES : 449
C.tropicalis     : -------------------DVDVLAKMKLLLWKLISYGVSVVPGYNMT----VD-QEYSKA : 474
F.graminearum    : HPDAGK--------------RSIVDIEEEIFNLCIENGVLIARGSWFL----TE-K-DKAP : 524
F.oxysporum      : HPLYQT----RGGDIAPVIDGTALANAFLIHSTHAPHLVLGSPGSMFS----ATPEIREKR : 573
M.oryzae         : HPLWKAPRPGKDGEPAGVMDGGVLSGALLAMLTKKPWLVLVAPGFMFS----ANEEIQRKD : 563
P.brasiliensis   : HPGVRE-------------GKDHAAIENAIFKATIEEGALVSAGSWFV----AD-S-SIPQ : 496
P.nodorum        : HPQYPE--------------KSIEEIEESIFYRNIEHGTMCMRGSWFY----AD-A-DEEH : 503
P.triticina      : ----------------------------------------------------NT------- : 351
R.oryzae         : -------------------LGGITDSYDLVMTKVLKENVLAIPGLAFM----PR-----GN : 384
S.cryophilus     : YKFADQ-------------GKSIAEIESDIHEEAIQDGANVACGNWFV----VD---SSIN : 436
S.japonicus      : YLHSDK-------------FKTVPEIETDIHEQAVDAGVNLACGHWFI----VD---SSKN : 442
S.pombe          : LICSNM-------------DKSISEIEMEIFVELVNNGVKPVCGQLFM----GE---PNSA : 436
S.punctatus      : --------------------LGITDSSDLIKQKAMEKKVLLVPGFEFF----PN-----PT : 393
T.equinum        : HPDYLE-----------KRTHLSLDLEDKIYERAASHGVAVAKGSWFN----VE----TCL : 455
T.rubrum         : HPAYQQ-------------GKDHKAIEEMIFLAAVDAGVLVSRGSWFL----AD-Q-SATE : 500
U.reesii         : HPLWSQ------------VPAAKLANALWKYLINAPYRVIVGPGWLFA----PT-EDVKKY : 548
V.dahliae        : HPDYPQ--------------RSLLDIEEDIFNKAIGNGVLCARGSWFR----TE-P-GTPA : 527
```

```
N.crassa          : NELFFRATFAAAT-AENMTEAIRRFGEAIRESFKL-------------------------- : 560
U.maydis          : KSCYVRTSFSIIA-EDDVEEAFNRLRNVVLDAWKDAGKPMPELA----------------- : 503
A.macrogynus      : PCAFVRATYSTAT-DEQMQEGMKRLRECIDLAMRDQQKQKQQAPKK--------------- : 512
A.nidulans        : GHQYFRLCFAAMP-ADDVVPITQRVVDGFRSFWQRKNLDGLEGDDGIAQTMEQLQMEPSAN : 584
B.cinerea         : VDMFFRATFAAAS-EEKMTEAIERFGVAVRNSFGIE------------------------- : 523
B.dendrobatidis   : ATPYVRASYSLAT-KEDIDLALQRLRELVLEARDKK------------------------- : 470
C.albicans        : RSNFFRLCYALANNDEEILESGKRLTDAVYEFFSNGLEF---------------------- : 523
C.guilliermondii  : TANFFRLTVAAANTDEELIAAGTQLAKAVTEFFENGLEF---------------------- : 511
C.immitis         : TDMFFRATFAAAP-ADKIQQAIKRFGDTLRAQFQLQ------------------------- : 535
C.neoformans      : KRIFVRLSFSFST-ADEMEEGVKRFARGLRKEWGIQNLDEVEH------------------ : 491
C.tropicalis      : RSNFFRLCVAIANNEDEILESGKRLTDGVLEFFNNGLEY---------------------- : 513
F.graminearum     : PGLFFRATYASAT-PENMNKAIERFGKAVRDSFGRK------------------------- : 559
F.oxysporum       : GWQYIRLCFAAES-DENIDGGASRFARSVGEFFKINDTAQIEKLLEELPS----------- : 622
M.oryzae          : GWAYFRLCFAAEA-EEAVDPCTQRFVDGIQRFWKIKTVREIEDLLKGFPISSGEASSMNVA : 623
P.brasiliensis    : TDMFFRATYAAAP-GDKIEEAIRRIGVALRSEFKLD------------------------- : 531
P.nodorum         : DTLFFRATYAASP-EDKMEEGIRRLGESVREEFGLGKK----------------------- : 540
P.triticina       : ------------------------------------------------------------- :   -
R.oryzae          : KNEYIRVSFSNVN-KPQMDEALRRLAMVIDKEAEIHNVKI--------------------- : 423
S.cryophilus      : DKIFFRVTFAYAE-LPEFETAIERFANVLKKFFKS-------------------------- : 470
S.japonicus       : DRVFFRVTFAAAN-FEDFNVAIERFAAVLNKNFK--------------------------- : 475
S.pombe           : DKIFFRFAYSLAD-LSTFEAGLERFTSTIQKYFQL-------------------------- : 470
S.punctatus       : TTPYVRASYSIAE-AEDMDEALRRLRELVEEARS--------------------------- : 426
T.equinum         : DKVFFRITFVSTTCLDDLENGVKRLGAAVRDEFRLAQA----------------------- : 493
T.rubrum          : EDMFFRSTFAAAP-ADKIREAIRRFGEVLKAQFNI-------------------------- : 534
U.reesii          : AWRYMRLCFAPVD-EGAVATSSHNFIEGCRSFWQVKDIHDIDDLEDDNEE----------- : 597
V.dahliae         : KDMFFRATFASAS-EEAMSTAIQRLGAAIQTSYRID------------------------- : 562
```

```
N.crassa          : ------------- :   -
U.maydis          : ------------- :   -
A.macrogynus      : ------------- :   -
A.nidulans        : FLGMGC------- : 590
B.cinerea         : ------------- :   -
B.dendrobatidis   : ------------- :   -
C.albicans        : ------------- :   -
C.guilliermondii  : ------------- :   -
C.immitis         : ------------- :   -
C.neoformans      : ------------- :   -
C.tropicalis      : ------------- :   -
F.graminearum     : ------------- :   -
F.oxysporum       : ------------- :   -
M.oryzae          : DQDVGNMGMYMGC : 636
P.brasiliensis    : ------------- :   -
P.nodorum         : ------------- :   -
P.triticina       : ------------- :   -
R.oryzae          : ------------- :   -
S.cryophilus      : ------------- :   -
S.japonicus       : ------------- :   -
S.pombe           : ------------- :   -
S.punctatus       : ------------- :   -
T.equinum         : ------------- :   -
T.rubrum          : ------------- :   -
U.reesii          : ------------- :   -
V.dahliae         : ------------- :   -
```
