# Supplementary material for: Characterization of indole-3-pyruvic acid pathway-mediated biosynthesis of auxin in Neurospora crassa
Source: PLoS One. 2018 Feb 8;13(2):e0192293. doi: 10.1371/journal.pone.0192293 (PMC5805262; doi:10.1371/journal.pone.0192293)
Supplement: S2 Fig — The black background with yellow font, red background with black font and green background with black fonts represent 100%, 75% and 50% identity of the residues, respectively. TPP binding residues are denoted by pink stars on top of the alignment. (PDF) [file pone.0192293.s002.pdf]

```
N.crassa      : -----------------------------------------------------------MS :   2
U.maydis      : ------------------------------------------------------------- :   -
A.gossypii    : ------------------------------------------------------------- :   -
A.nidulans    : -----------------------------------------------------------MA :   2
A.niger       : ----------------------------------------------------------MAT :   3
A.oryzae      : ----------------------------------------------------------MAT :   3
B.cinerea     : -----------------------------------------------------------MV :   2
C.albicans    : ------------------------------------------------------------- :   -
C.glabrata    : ------------------------------------------------------------- :   -
C.globosum    : -----------------------------------------------------------MT :   2
C.tropicalis  : ------------------------------------------------------------- :   -
F.oxysporum   : ------------------------------------------------------------- :   -
K.africana    : ------------------------------------------------------------- :   -
K.lactis      : ------------------------------------------------------------- :   -
M.acridum     : -----------------------------------------------------------MA :   2
M.gypseum     : ----------------------------------------------------------MAS :   3
M.thermophila : -----------------------------------------------------------MT :   2
N.dairenensis : ------------------------------------------------------------- :   -
N.tetrasperma : -----------------------------------------------------------MS :   2
P.anserina    : ----------------------------------------------------------MAI :   3
S.cerevisiae  : MVMAHFCINLAVLVEHRKKXIYKQGSFTLLAIRFGFLPFIFIFLVIFFSQLLFSTHNLTQN :  61
S.cryophilus  : ------------------------------------------------------------- :   -
S.japonicus   : ------------------------------------------------------------- :   -
S.macrospora  : -----------------------------------------------------------MS :   2
S.pombe       : ------------------------------------------------------------- :   -
T.blattae     : ------------------------------------------------------------- :   -
T.delbrueckii : ------------------------------------------------------------- :   -
T.terrestris  : ----------------------------------------------------------MGL :   3
U.hordei      : ------------------------------------------------------------- :   -
V.polyspora   : ------------------------------------------------------------- :   -
Z.rouxii      : ------------------------------------------------------------- :   -

                                                 *                         *
N.crassa      : DIRTQSLQKPVTVAEYLFRRLHQIGIRSVHGLPGDFNLVALDYVPKA---GLKWVGSVNEL :  60
U.maydis      : ---MSNESNQIKIGAYLLERLVQLGSQSVQGVPGDFNMGFLDLIEEH--PKLKWIGNSNEL :  56
A.gossypii    : -------MSEITLGRYLFERLKQVEVRTIFGVPGDFNLSLLDKVYEV--DGMRWAGNANEL :  52
A.nidulans    : DIATRELRQPIDIAEYLFRRLHEVGIRSVHGVPGDYNLAALDYLPKC---GLHWVGNCNEL :  60
A.niger       : DIATRDLRKPITVAEYLFRRLHEVGVRSVHGVPGDYNLAALDYLPKC---GLHWVGNCNEL :  61
A.oryzae      : DIATRDLRKPIDVAEYLFRRLREVGVRAVHGVPGDYNLVALDYLPKC---DLHWVGNCNEL :  61
B.cinerea     : DLRTESLKSPVDLAEYLFTRLKQIGIDSVHGLPGDYNLVALDYIPKL---GLKWVGNCNEL :  60
C.albicans    : -------MSEITLGRFFFERLHQLKVDTVFGLPGDFNLALLDKIYEV--EGMRWAGNANEL :  52
C.glabrata    : -------MSEITLGRYLFERLNQVDVKTIFGLPGDFNLSLLDKIYEV--EGMRWAGNANEL :  52
C.globosum    : DIREQGLKKPIAITEYLFKRLHEIGIRSVHGLPGDFNLVALDYIPKA---GLRWVGSVNEL :  60
C.tropicalis  : -------MSEITLGRFFFERLHQLQVDTVFGLPGDFNLALLDKIYEV--DGMRWAGNANEL :  52
F.oxysporum   : -MSNSNLQKPIDVAEYLFKRLYEVGVRSVHGVPGDYNLVALDYLPQC---NLKWVGSVNEL :  57
K.africana    : -------MAEITLGKYLFERLAQVDVKTIFGLPGDFNLSLLDKIYEV--PGMRWAGNANEL :  52
K.lactis      : -------MSEITLGRYLFERLKQVEVQTIFGLPGDFNLSLLDNIYEV--PGMRWAGNANEL :  52
M.acridum     : DIRVQSLRKPIDVSEYLFRRLYEVGVRSVHGVPGDYNLVALDYLPKC---GLKWVGSVNEL :  60
M.gypseum     : DIVTRELEEPVDVAEYLFQRLHQMGIKSVHGVPGDYNLAALDYLPKC---GLHWVGNCNEL :  61
M.thermophila : DIREQGLKKPVNVAEYLFRRLHEIGIRSVHGLPGDFNLVALDYIPKA---GLKWVGSVNEL :  60
N.dairenensis : -------MSEITLGRYLFERLSQVDVKTIFGLPGDFNLSLLDKIYET--PGMRWAGNANEL :  52
N.tetrasperma : DIRTQSLQKPVTVAEYLFRRLHQIGIRSVHGLPGDFNLVALDYVPKA---GLKWVGSVNEL :  60
P.anserina    : DIRTQGLKKPVPVAQYLFARLYEIGIRSVHGLPGDFNLVALDYIPKA---KLKWVGSVNEL :  61
S.cerevisiae  : NTVKSIKMSEITLGKYLFERLKQVNVNTVFGLPGDFNLSLLDKIYEV--EGMRWAGNANEL : 120
S.cryophilus  : ------MAGEILVGEYLFERIRQLGVKSIQGVPGDFNLALLDLIDNVGDESFRWVGNTNEL :  55
S.japonicus   : ----MSGKGEILVGEYLFQRLLELGVKSILGVPGDFNLALLDLIEKIGDDSFRWVGNTNEL :  57
S.macrospora  : DIRTQSLQKPVTVAEYLFRRLHQIGIRSVHGLPGDFNLVALDYVPKA---GLKWVGSVNEL :  60
S.pombe       : ------MSGDILVGEYLFKRLEQLGVKSILGVPGDFNLALLDLIEKVGDEKFRWVGNTNEL :  55
T.blattae     : -------MAEITLGKYLFERLNQVKVQTIFGLPGDFNLSLLDKIYEV--PGMRWAGNANEL :  52
T.delbrueckii : -------MSEITLGRYLFERLKQVDTNTIFGLPGDFNLSLLDKIYEV--PGMRWAGNANEL :  52
T.terrestris  : DIRERGLKKPVAVAEYLFRRLHEIGIRSVHGLPGDFNLVALDYIPKA---GLRWVGSVNEL :  61
U.hordei      : -----MSSNEIKIGAYLLERLVQLGTQSIQGVPGDFNMGFLDLIEDH--PSLEWIGNSNEL :  54
V.polyspora   : -------MSEITLGRYLFERLKQVQVQTIFGLPGDFNLSLLDKIYEV--PGMRWAGNANEL :  52
Z.rouxii      : -------MSEITLGRYLFERLKQVDTNTIFGVPGDFNLSLLDKVYEV--QGLRWAGNANEL :  52
                                      *  *
N.crassa      : NAAYAADGYARA--KGISALVTTFGVGELSAINGVAGAYSEHVPIVHIVGCPSTISQRNGM : 119
U.maydis      : NAAYAADGYARVK-RTIAAVVTTFGVGELSALNGIAGSFSERLPVIHVVGVPSTGAQGSHS : 116
A.gossypii    : NASYAADGYARV--KKISCLVTTFGVGELSALNGIAGSYAEHVGVLHVVGVPSTSAQAKQL : 111
A.nidulans    : NAGYAADGYARV--NGIAALVTTFGVGELSAINAIAGAYSEFVPIIHIVGQPHSRSQKDGL : 119
A.niger       : NAGYAADGYARV--NGIGALITTFGVGELSALNAIAGSYSEFVPVVHIVGQPNTKSQKDGM : 120
A.oryzae      : NAGYAADGYARI--NGMSALVTTFGVGELSALNAIAGAYSEFVPIVHIVGQPHTKSQKDGM : 120
B.cinerea     : NAGYAADGYARV--KGISAIMTTFGVGELSAINAIAGAYSERVPIVHIVGTPSTISQKDGM : 119
C.albicans    : NAGYAADGYARVNPNGLSALVSTFGVGELSLTNAIAGSYSEHVGVINLVGVPSSSAQAKQL : 113
C.glabrata    : NAAYAADGYARI--KGMSCIITTFGVGELSALNGIAGSYAEHVGVLHVVGVPSISSQAKQL : 111
```

```
C.globosum    : NAAYAADGYART--KGISAILTTFGVGELSAINGIAGAFSEHVPVVHIVGCPSTISQRNGM : 119
C.tropicalis  : NAGYAADGYARVNPNGLAALVSTFGVGELSLTNAIAGSYSEHVGIINLVGVPSSSAQAKQL : 113
F.oxysporum   : NAAYAADGYARV--TKMAALITTFGVGELSAVNGVAGSYSEHIPVVHIVGCPSTISQRDQM : 116
K.africana    : NAAYAADGYARI--KGMSCIITTFGVGELSALNGIAGSYAEHVGVLHVVGVPSISSQAKQL : 111
K.lactis      : NAAYAADGYARL--KGMSCIITTFGVGELSALNGIAGSYAEHVGVLHVVGVPSVSSQAKQL : 111
M.acridum     : NAAYAADGYARI--KQIGALITTFGVGELSAINGVAGAFSEHIPVVHIVGYPSTLSQRDGM : 119
M.gypseum     : NAGYAADGYARI--NGMSALITTFGVGELSALNAIAGAYSEFVPIVHIVGQPSTASQKDGM : 120
M.thermophila : NAAYAADGYART--KGISAIFTTFGVGELSAINGIAGAFSEHVPVVHIVGCPSTISQRNGM : 119
N.dairenensis : NAAYAADGYARI--KGMSCIITTFGVGELSALNGIAGSYAEHVGVLHVVGVPSVSSQAKQL : 111
N.tetrasperma : NAAYAADGYARA--KGISALVTTFGVGELSAINGVAGAYSEHVPIVHIVGCPSTISQRNGM : 119
P.anserina    : NAAYAADGYARA--LGISALVTTFGVGELSAMNGVAGAYSEHVPVVHIVGCPSTISQRNGM : 120
S.cerevisiae  : NAAYAADGYARI--KGMSCIITTFGVGELSALNGIAGSYAEHVGVLHVVGVPSISAQAKQL : 179
S.cryophilus  : NGAYAADGYARV--KGLSAIVTTFGVGELSAINGLAGAYAEHVPIVHIVGMPSTKAQSSGA : 114
S.japonicus   : NGAYAADGYARV--KGISAIVTTFGVGELSAINGLAGAYAEHVPVVHIVGMPSTKSQASGA : 116
S.macrospora  : NAAYAADGYARA--KGISAIVTTFGVGELSAINGVAGAYSEHVPIVHIVGCPSTISQRNGM : 119
S.pombe       : NGAYAADGYARV--NGLSAIVTTFGVGELSAINGVAGSYAEHVPVVHIVGMPSTKVQDTGA : 114
T.blattae     : NAAYAADGYARI--KGMACLITTFGVGELSALNGIAGSYAEHVGVLHVVGVPSLSSQAKQL : 111
T.delbrueckii : NAAYAADGYARV--KGMAALVTTFGVGELSALNGIAGSYAEHVGVLHIVGVPSISSQAKQL : 111
T.terrestris  : NAAYAADGYARI--KGISALVTTFGVGELSAINGVAGAFSEHVPVVHIVGCPSTISQRNGM : 120
U.hordei      : NAAYAADGYARIK-RTISAVVTTFGVGELSALNGIAGSFSERLPVIHIVGVPSTAAQGAHS : 114
V.polyspora   : NAAYAADGYARI--KGMACLITTFGVGELSALNGIAGSYAEHVGVLHVVGVPSLSSQAKQL : 111
Z.rouxii      : NAAYAADGYARV--KGLAALITTFGVGELSALNGIAGSYAEHVGVLHIVGVPSVSSQAKQL : 111
```

```
N.crassa      : LLHHTLGNGDFHVFANMSENISCDVAKLVKPSEIAYQIDHAIRECWIRSRPVYIWLPTDMV : 180
U.maydis      : LLHHTLGDGRFSAFENMSKEISADSAILKSKQGAGESIDRILITAMKSARPVYLALPTDLV : 177
A.gossypii    : LLHHTLGNGDFTVFHRMSANISDTTAVITDISSAPAEIDRCIRACYVHQRPVYLGLPANMV : 172
A.nidulans    : LLHHTLGNGDYNVFSSMNKGISVTTANLNDTYDAATLIDNAIRECWIHSRPVYLALPTDMI : 180
A.niger       : LLHHTLGNGDFNVFAKMSAGISCTLGRLNETLEAATLIDNAIRECWIRSRPVYISLPTDMI : 181
A.oryzae      : LLHHTLGNGDFNVFTRMSADISCTLGCLNSTHEVATLIDNAIRECWIRSRPVYISLPTDMV : 181
B.cinerea     : LLHHTLGNGNFNVFADMSKEISCAMAKINDPHEAAALIDHTLQQCWVHSQPVYITLPTDMV : 180
C.albicans    : LLHHTLGNGDFTVFHRMFKNISQTSAFIADINSAPAEIDRCIRDAYVYQRPVYIGLPSNLV : 174
C.glabrata    : LLHHTLGNGDFTVFHRMSANISETTAMVTDIATAPAEIDRCIRTTYITQRPVYLGLPANLV : 172
C.globosum    : LLHHTLGNGDFNVFANMSSQISCDMARLNKPAEIPDQIDHALRECWIRSRPVYIMLPTDMA : 180
C.tropicalis  : LLHHTLGNGDFTVFHRMFKNISQTSAFISDPNTAASEIDRCIRDAYVYQRPVYIGLPSNLV : 174
F.oxysporum   : LLHHTLGNGDFDVFANMSAQISCNVAKLNKPSEIAEQIDTALRTCWLRSRPVYIMVPTDMV : 177
K.africana    : LLHHTLGNGDFTVFHRMSANISETTAMVTDIATAPAEIDRCIRTTYVTQRPVYLGLPANLV : 172
K.lactis      : LLHHTLGNGDFTVFHRMSSNISETTAMITDINTAPAEIDRCIRTTYVSQRPVYLGLPANLV : 172
M.acridum     : LLHHTLGNGDFNVFANMSSQISCQVAKLNRPSEIADQIDHALRECWVHSRPVYITLPTDMA : 180
M.gypseum     : LLHHTLGNGDFNAFANMSAGISCSVAKLNDPRDAAAYIDSTLRECWVRSRPVYITLPIDIV : 181
M.thermophila : LLHHTLGNGDFNVFANMSSQISCDVARLNKRAEIADQIDHALRECWIRSRPVYIMLPTDMV : 180
N.dairenensis : LLHHTLGNGDFTVFHRMSANISETTAMVTDIATAPAEIDRCIRTTYVTQRPVYLGLPANLV : 172
N.tetrasperma : LLHHTLGNGDFHVFANMSENISCDVAKLVKPSEIAYQIDHAIRECWIRSRPVYIWLPTDMV : 180
P.anserina    : LLHHTLGNGDFNVFANMGSQIACNTARLNNPAEIAEQIDFALRECWIHSRPVYIMLPTDMV : 181
S.cerevisiae  : LLHHTLGNGDFTVFHRMSANISETTAMITDIATAPAEIDRCIRTTYVTQRPVYLGLPANLV : 240
S.cryophilus  : LLHHTLGNGDFGIFVEMNRPVSAYTTVITNGEDAAAKIDEALTICYRKARPVYIGIPADTA : 175
S.japonicus   : LLHHTLGNGDFTVFMEMSEKVSAYTVMLTDGETAADKIDKALSISYRKARPVYIGIPSDVG : 177
S.macrospora  : LLHHTLGNGDFNVFANMSEHISCDVAKLVKPGEIAQQIDHAIRECWIRSRPVYIWLPTDMV : 180
S.pombe       : LLHHTLGDGDFRTFMDMFKKVSAYSIMIDNGNDAAEKIDEALSICYKKARPVYIGIPSDAG : 175
T.blattae     : LLHHTLGNGDFTVFHRMSAEISETTAMITDIATAPAEIDRCIRTTYVKQRTVYLGLPANLV : 172
T.delbrueckii : LLHHTLGNGDFTVFHRMSANISETTAMITDINSAPAEIDRCIRTTYVSQRPVYLGLPANLV : 172
T.terrestris  : LLHHTLGNGDFNVFANMGSQIACDTARLNKPAEIADQIDHALRECWIRSRPVYIMLPTDMV : 181
U.hordei      : LLHHTLGDGRFSAFVNMSKEISADSAVLKQKEGVGEAIDRILVSAMKKARPVYLALPTDLV : 175
V.polyspora   : LLHHTLGNGDFTVFHRMSACISETTAMITDIANAPAEIDRCIRTTYITQRTVYLGLPANMV : 172
Z.rouxii      : LLHHTLGNGDFTVFHRMSANISETTAMLTDITAAPAEIDRCIRVAYVNQRPVYLGLPANLV : 172
N.crassa      : EKKIEGARLDTEIDLSEPENDPDREDYVVDVVLRYLHGAKNPVVLVDACAIRHRVTEEVKR : 241
U.maydis      : HATIPAEALKTPLDYSIDDNDAKAEEYVLQVAQKHIADAKSAVILVDACAARHGCIQETHE : 238
A.gossypii    : DLTVPASLLNTEIDLSLKPNDPEAEEEVVSTVLELVANAKHPVILSDACASRHDVKQETKQ : 233
A.nidulans    : TKKIEGERLKTPIDLSLPANDPEKEDYVVDVVLKYLHAAKNPVILVDACAIRHRVLEEVHD : 241
A.niger       : VKQIEGDRLDKPLDLSLPANDPEKEDYVVDVVLKYLHAAKKPVILVDACAIRHRVLDEVHD : 242
A.oryzae      : TKKIEGERLDTPLDLSLPPNDPEKEDYVVDVVLKYLHAAKKPVILVDACAIRHRVLDEVHE : 242
B.cinerea     : QKKVEGERLKTPIDLSLPSNDPDKEDYVVDVILKYFEAAKNPIILVDACAIRHRVIPEVHG : 241
C.albicans    : DMKVPKSLLDKKIDLSLHPNDPESQTEVIETVEKLISEASNPVILVDACAIRHNCKPEVAK : 235
C.glabrata    : DLKVPAKLLETPIDLSLKPNDPEAETEVVDTVLELIKAAKNPVILADACASRHDVKAETKK : 233
C.globosum    : EKKVEGARLDTPIDLAEPENDPEREDYVVDVVLKYLHAAKSPVILVDACAIRHRVLEEVHD : 241
C.tropicalis  : DVKVPKSLLDKKIDLSLHPNEPESQAEVVETVEKFISEASNPVILVDACAIRHNCLKEVAE : 235
F.oxysporum   : QEKVEGARLDTPIDLSEPQNDPGNEDFVVDEILKAMYAAQRPVILVDSCAIRHRVVKEVHQ : 238
K.africana    : DLKVPASLLETPIDLSLKPNDAEAEKEVVDTILSLIKDAKNPVILSDACASRHDVKAETKQ : 233
K.lactis      : DLTVPASLLDTPIDLSLKPNDPEAEEEVIENVLQLIKEAKNPVILADACCSRHDAKAETKK : 233
M.acridum     : DAKVEGARLDRPIDLTEPQNDPEKEDYVVDVIMKYLNAAKNPVLLVDACAVRHRVLPEVHD : 241
M.gypseum     : KQKIEGKRLKTPIDLQLPANDEEKEDYVVDVVLKYLQAAKKPAIIVDACAIRHNVLDEVHD : 242
M.thermophila : ERKVEGARLDTPIDLTEPANQSEREDYVVDVVLRYLHAAKQPVILVDACAIRHRVLKEVHD : 241
N.dairenensis : DLKVPASLLETPIDLSLKANDVEAETEVVNTILELIKDAKNPVILADACASRHDVKAETKK : 233
N.tetrasperma : EKKIEGARLDTEIDLSEPENDPDREDYVVDVVLRYLHGAKNPVVLVDACAIRHRVTEEVKR : 241
```

```
P.anserina      : EKQIEGARLDTPIDLSDPPNEPEREDYVVDVVLRYLHAAKNPIILVDACAIRHRCLEEVRN : 242
S.cerevisiae    : DLNVPAKLLQTPIDMSLKPNDAESEKEVIDTILALVKDAKNPVILADACCSRHDVKAETKK : 301
S.cryophilus    : YFKTSSANLRKPLETEEPENEPVSEQEVITTVAEMIEKSKKPVILVDACAIRHRVVPEVKQ : 236
S.japonicus     : YFKTSSAGLKKPLQLEEPANDPKIEEEVVHTIVQMINASKKPVILADACVTRHRVIKELHE : 238
S.macrospora    : EKKIEGARLDTEIDLSEPENDPDREDYVVDVVLKYLHGAKNPVVLVDACAIRHRVTEEVNK : 241
S.pombe         : YFKASSSNLGKRLKLEEDTNDPAVEQEVINHISEMVVNAKKPVILIDACAVRHRVVPEVHE : 236
T.blattae       : DLTVPASLLETPIDLSLKPNDAEAENEVIETVLELVREAKNPIIIADACCSRHDVKSETEE : 233
T.delbrueckii   : DLKVPASLLDTPIDLSLKANDAEAEQEVVDDVLALIKAAKNPVILADACCSRHDVKDETRK : 233
T.terrestris    : EKKIEGARLDTPIDLTEPENEAEREDYVVDVVLKYLHAAKCPIILVDACAVRHRVLKEVHD : 242
U.hordei        : HATIPADALKTKLKYDAEPNDEAAEKYVLNVAKKHIEQAKSAVILVDACADRHGCIAETRE : 236
V.polyspora     : DLMVPADLLKTPIDLSLKPNDPEAETEVLDTILAMVKEAKNPIILADACASRHDVKAETKQ : 233
Z.rouxii        : DQKVPASLLNTPIDLSLKENDPEAETEVVDTVLELIKEAKNPVILADACCSRHDVKAETKK : 233


N.crassa        : FIEKTKLPVFVTPMGKGAFDETSEHYGGVYAGTGSLP-EVAKRVEGSDLVLSIGAIKSDFN : 301
U.maydis        : LIEKSGLPVFATPMGKTIVDEDHAQYGGIYVGSLTSE-KVKNVVEQADVLITVGSLKSDFN : 298
A.gossypii      : LIDVTQFPAFVTPMGKGSIDEQHPRFGGVYVGTLSAP-DVKEAVESADLILSVGALLSDFN : 293
A.nidulans      : LIEVSGLPTFVAPMGKGAVNETHRCYGGVYAGTGSNP-GVREQVESSDLILSIGAIKSDFN : 301
A.niger         : LMEASGLPTFVAPMGKGAVDETRPNYGGVYAGTGSNA-GVREQVESSDLILSIGAIKSDFN : 302
A.oryzae        : FVEKSGLPTFVAPMGKGAVDETHKNYGGVYAGTGSNP-GVREQVESSDLILSIGAIKSDFN : 302
B.cinerea       : LIEKTGLPVFVTPMGKGAVDETSENYGGVYAGSGSHP-DVKHRVESSDLILTIGAIKSDFN : 301
C.albicans      : LIEETQFPVFTTPMGKSSVDESNPRFGGVYVGSLSKP-EVKESVESADLILSIGALLSDFN : 295
C.glabrata      : LIDATQFPSFVTPMGKGSIDEQHPRFGGVYVGTLSRP-EVKEAVESADLILSVGALLSDFN : 293
C.globosum      : LVEKTQLPVFVTPMGKGAINENHPSYGGVYAGTGSQP-AVAERVESADLVLSIGALKSDFN : 301
C.tropicalis    : LIAETQFPVFTTPMGKSSVDESNPRFGGVYVGSLSSP-DVKEAVESADLVLSVGAMLSDFN : 295
F.oxysporum     : LIDKLDLPVFVTPMGKGAVNEDHPNYGGVFAGDGSHPARAQSIVEGSDLLLTIGALKSDFN : 299
K.africana      : LIDATQFPAFTTPMGKGSIDEQHPRFGGVYVGTLSRP-EVKEAVESADLILSVGALLSDFN : 293
K.lactis        : LIDLTQFPAFVTPMGKGSIDEKHPRFGGVYVGTLSSP-AVKEAVESADLVLSVGALLSDFN : 293
M.acridum       : LLAKTNLPVFVTPMGKSAVNEQHDNFGGVYAGSGSHPPEVKNIVESSDLVVSIGALKSDFN : 302
M.gypseum       : LVSKSGLPTFVAPMGKGAVDETLPNYGGVYAGDGSTV-EVQKHIEASDLILSIGAVKSDFN : 302
M.thermophila   : LVEKTQLPVFVTPMGKGAINEDHPNYGGVYAGTGSQP-AVAERVETADLVLSIGALKSDFN : 301
N.dairenensis   : LIDITQFPSFVTPMGKGSIDEQHPRFGGVYVGTLSSP-AVKEAVESADLILSVGALLSDFN : 293
N.tetrasperma   : FIEKTKLPVFVTPMGKGAFDETSEHYGGVYAGTGSLP-EVAKRVEGSDLVLSIGAIKSDFN : 301
P.anserina      : LVDKAKLPVFVTPMGKGAVNESSPTYGGVYAGTGSQP-AVQELVESADLVLSIGALKSDFN : 302
S.cerevisiae    : LIDLTQFPAFVTPMGKGSIDEQHPRYGGVYVGTLSKP-EVKEAVESADLILSVGALLSDFN : 361
S.cryophilus    : LIELTHFPSYVTPMGKSALNETSKYFDGVYIGSISDP-QVKERIESTDLLLSVGALKSDFN : 296
S.japonicus     : LINLTHFPTYVTPMGKSSVDEVSEWFDGVYVGSISDP-AVKDRIESSDLILSVGGLKSDFN : 298
S.macrospora    : FIEKTKIPVFVTPMGKGAFDETCEHYGGVYAGTGSLP-EVAKRVEGSDLVLSIGAIKSDFN : 301
S.pombe         : LIKLTHFPTYVTPMGKSAIDETSQFFDGVYVGSISDP-EVKDRIESTDLLLSIGALKSDFN : 296
T.blattae       : LINATQFPSFTTPMGKGSINERHPRFGGVYVGTLSRP-EVKAAVESADLVLSVGALLSDFN : 293
T.delbrueckii   : LIDITQFPSFVTPMGKGSIDEQNPRFGGVYVGTLSSP-EVKEAVESADLILSVGALLSDFN : 293
T.terrestris    : LVEKTQLPVFVTPMGKGAINESHPSYGGVYAGTGSHP-DVAERVESADLVLSIGALKSDFN : 302
U.hordei        : LIEKSGLPVFATPMGKAIVDEDHPQYGGLYVGNLTSE-KVKEVVEGADVLITVGSLKSDFN : 296
V.polyspora     : LIDTTQFPCFVTPMGKGSIDEQHPRFGGVYVGTLSRP-EVKAAVESADLILSVGALLSDFN : 293
Z.rouxii        : LIDLTQFPSFVTPMGKGSIDEQNPRFGGVYVGTLSSP-EVKEAVESADLVLSVGALLSDFN : 293

N.crassa        : TAGFSYHTSQLNTIDLHSDHCTVRYSEYPGVAMRGVLRKVTERIDMSKL-SITPSPPVEN- : 360
U.maydis        : SGNFSYRTPKSSTIELHSDYTTIGYSHYPGIGMKKLLPKLSALLETNGDARREETKKIVPK : 359
A.gossypii      : TGSFSYSYKTKNIVEFHSDHTKIRSATFPGIKMKTVLQNLVKRIGEAAK-GYQPSPVPTYA : 353
A.nidulans      : TAGFSYRIGQLNTIDFHSTYVRVRYSEYPDTNMKGVLRKVIQRLG--FI-KADPVPHISNA : 359
A.niger         : TSGFSYHIGQLNTIDFHSTYVRVRYSEYPEINMKGVLRKVIQRMG--AV-NAAPVPHLSNT : 360
A.oryzae        : TTGFSYRIGQLNTIDFHSTYVRVRYSEYPDINMKGVLQKIVQRMG--NL-NVGPVSPPSNL : 360
B.cinerea       : TAGFSYKTSQLNTIDFHSTKIAVRYSEYPGVHMRGVLQKIIKKVDLRKL-SAVPGPKMEN- : 360
C.albicans      : TGSFSYGYKTRNIVEFHSDYTKIRQATFPGVQMKEALQKLLTTVKKSINPNYTPVPVPETK : 356
C.glabrata      : TGSFSYSYKTKNIVEFHSDYIKIRNATFPGVQMKFALQKLLNAVPEAIK-GYKPVPVPARV : 353
C.globosum      : TAGFSYRTSQLNTIDFHSTHCTVRYSEYPGVAMRGVLRKVNERVDLTKL-CRPSSPDVAN- : 360
C.tropicalis    : TGAFSYNYKTRNVVEFHSDYTKIRQATFPGVQMKEALQVLLKTVKKSVNPKYVPAPVPAT- : 355
F.oxysporum     : TTGFSYRTSQINSVDFHSTHCKVRYSTYPGVAMRGVLRKIIDRVDPKSM-PAPSIPEVRN- : 358
K.africana      : TGSFSYSYKTKNIIEFHSDYTKIRNATFPGVQMKFVLRKLLASVADAAK-GYKPVAVPARV : 353
K.lactis        : TGSFSYSYKTKNIVEFHSDYTKIRSATFPGVQMKFALQKLLTKVADAAK-GYKPVPVPSEP : 353
M.acridum       : TAGFSYRTSQLNSIDLHSDHCVVRYSTYPGVRMKGVLQKIVKRVDPKQL-SIRASPTVRN- : 361
M.gypseum       : TTGFTYRVSRLNTIDFHSNHMVVRYSEYPGVSMKGVLRKVVNRMG--KL-NVS-APPKHEN : 359
M.thermophila   : TAGFSYRTSQLNTIDFHSDHCTVRYSEYPGVAMRGVLRKVVERVDLSKL-SRPPSPEVVN- : 360
N.dairenensis   : TGSFSYSYKTKNIVEFHSDYIKIKNATFPGVQMKFALQKLLGSVAEAAK-GYKPVAVPAKP : 353
N.tetrasperma   : TAGFSYHTSQLNTIDLHSDHCTVRYSEYPGVAMRGVLRKVTERIDMSKL-SITPSPPVEN- : 360
P.anserina      : TTGFSYRTSQLNTIDFHSDHCKVRYSEYPGVAMKGVLRKLIERVDTTKLSSEKLAPKVVN- : 362
S.cerevisiae    : TGSFSYSYKTKNIVEFHSDHMKIRNATFPGVQMKFVLQKLLTTIADAAK-GYKPVAVPART : 421
S.cryophilus    : TGSFSYHLSQKNSVEFHSDHMKIRYALYPEVSMKYVLRKLMNVLNPEKC-RAKAAPTLGYD : 356
S.japonicus     : TGSFSYHISQKNTIEFHSDHTKIRYALYPGISMKYLLRRVISSLDAKAM-RAKAAKAIGFH : 358
S.macrospora    : TAGFSYHTSQLNTIDLHSDHCTVRYSEYPGVAMRGVLRKVTERIDTSKL-SITPSPQVEN- : 360
S.pombe         : TGSFSYHLSQKNAVEFHSDHMRIRYALYPNVAMKYILRKLLKVLDASMC-HSKAAPTIGYN : 356
T.blattae       : TGSFSYAYKTKNIVEFHSDHIKVKNATFPGVQMKFVLQKLVSKIGAAIK-GYKPVAVPAGI : 353
T.delbrueckii   : TGSFSYSYKTKNIVEFHSDYIKIRNATFPGVQMKFALQKLLAKVGDVAK-DYKPVQVPART : 353
T.terrestris    : TTGFSYRTSQLNTIDFHSTYCQVRYSEYPGVAMRGVLRKVVERVDLSKL-SQPSSPQVVN- : 361
```

```
U.hordei      : SGNFSYRTPKESTIELHSDYTTVGYSHYPGIGMKGLLPKLSAILQPDQARRLEETKTVVPK : 357
V.polyspora   : TGSFSYSYQTKNVVEFHSDHTKIKKASFPGVQMKFVLQKLVAQIGAAVK-DYKPVAAPALP : 353
Z.rouxii      : TGSFSYSYKTKNVVEFHSDHIKIRNATFPGVQMKFVLKKLLQAVPEAVK-NYKPGPVPAPP : 353

                                                    *                       *
N.crassa      : --AVAENRDDSQAITQAWFWPRVGEYFQEGDVVVTETGTANFGIWESRFPKDVMGVTQVLW : 419
U.maydis      : F-ENALPHDSSSTITQEWLWPRMGQFFQEQDQVIVETGTSSFGMLEAKLPAKTRWVSQVLW : 419
A.gossypii    : --APNEDADPKTPLKQEWLWNQVSSFLKEGDIVITETGTAAFGINQTRFPSSTIGISQVLW : 412
A.nidulans    : L-PEHEKNSSEQRITHAWMWPMVGQWLKENDIVITETGTANFGIWDTRFPSGVTAISQVLW : 419
A.niger       : L-PESEKSSSSQEITHDWLWPNVGQWLKENDIVITETGTANFGIWETRFPANVTAISQVLW : 420
A.oryzae      : L-PDNEKASTEQAITHAWLWPTVGQWLKEKDVVITETGTANFGIWDTRFPAGVTAISQVLW : 420
B.cinerea     : --KVTANEDSSETITQAWFWPRAGEFLKENDIVITETGTANFGIWETKFPKGVTALSQVLW : 419
C.albicans    : --LINTPAAPSTPLTQEYLWTKVSSWFREGDIIITETGTSAFGIVQSRFPKNSIGISQVLW : 415
C.glabrata    : --PENKSCDPATPLKQEWMWNQVSKFLQEGDVVITETGTSAFGINQTPFPNNAYGISQVLW : 412
C.globosum    : --EVTKNRDSSETITQAFFWPRVGEYLKEDDIVVTETGTSNFGIWETKYPRGVTGVTQILW : 419
C.tropicalis  : --KAITTPGNNDPVSQEYLWRKVSDWFQEGDVIISETGTSAFGIVQSKFPKNAIGISQVLW : 414
F.oxysporum   : --EVEKNTDDSEIITQAWLWPRVGEYLIPNDIVVTETGTANFGIWDTRFPRNVTALSQVLW : 417
K.africana    : --PENKACDPSTPLAQEWMWNQCAKFFQEGDVIITETGTSAFGINQSLFPNNSVGISQVLW : 412
K.lactis      : --EHNEAVADSTPLKQEWVWTQVGEFLREGDVVITETGTSAFGINQTHFPNNTYGISQVLW : 412
M.acridum     : --EAKEHHDESATITHAWFWPRLGEFLTDNDIVVTETGTSNFGIWDTKFPSGVTAMSQVLW : 420
M.gypseum     : L-PEENPQFPAPTISHSWLWPAVGNWLQENDIVITETGTSSFGIWGTRFPKGVTAISQVLW : 419
M.thermophila : --EVTKNRDSSQTITQAFFWPRIGEYLKENDIVVTETGTSNFGIWETKYPRGVTGITQILW : 419
N.dairenensis : --AANAAVDAATPLKQEWMWNQVGKFLQEGDVVITETGTSAFGINQTHFPNNTYGISQVLW : 412
N.tetrasperma : --AVAENRDDSQAITQAWFWPRVGEYFQEGDVVVTETGTANFGIWESRFPKDVMGVTQVLW : 419
P.anserina    : --EVSENRDSTETITQAWFWPRVGEYFKEKDLVVTETGTSNFGIWESKFPPDVVGITQILW : 421
S.cerevisiae  : --PANAAVPASTPLKQEWMWNQLGNFLQEGDVVIAETGTSAFGINQTTFPNNTYGISQVLW : 480
S.cryophilus  : IKPKHAEGYSDQQITQSWFWPNFGHFLKPKDVVVTETGTANFGILDCRFPPDVTAISQVLW : 417
S.japonicus   : VKPVHAEGFPKDSITHQWFWPKFSEFLIPRDVVVTETGTANFGVLDVRFPHNVTAISQVLW : 419
S.macrospora  : --AVAENKDDSQTITQAWFWPRVGEFLQEGDVVVTETGTANFGIWESKFPKGVMGVTQVLW : 419
S.pombe       : IKPKHAEGYSSNEITHCWFWPKFSEFLKPRDVLITETGTANFGVLDCRFPKDVTAISQVLW : 417
T.blattae     : --PANKEVPANTPLKQEWLWNQLGNFLQEGDIVLTETGTSAFGINQTTFPKDTYGISQVLW : 412
T.delbrueckii : --PENAATADSTPLKQQWIWNQVGKFLQEGDVVITETGTSAFGINQSHFPNKTYGISQVLW : 412
T.terrestris  : --KVAENWDDSETITQAFFWPRIGEYLKENDIVVTETGTANFGIWETKFPPGVTGVTQILW : 420
U.hordei      : F-QNALPSDSSDVISQEYLWPRMGRFFKEQDQVIVETGTSSFGMLEAKLPKNTRWVSQVLW : 417
V.polyspora   : --ASNAECPASTPLKQEWLWNQVGKFLQEGDIVLTETGTSAFGINQTHFPNNTYGISQVLW : 412
Z.rouxii      : --SPNAEVADSTTLKQEWLWRQVGSFLREGDVVITETGTSAFGINQTHFPNQTYGISQVLW : 412

                *  *                          ****
N.crassa      : GSIGWSVGAAQGAALAVKD-LEQDRRTILFVGDGSFQLTCQEVSTMLKHN-LRVTMLH--- : 475
U.maydis      : GSIGWSVGATLGVALAARE--NALGRTHLFVGDGSLQLTVQEIGTMIKHG-LCPYLFILNN : 477
A.gossypii    : GSIGFTTGACLGAAFAAEE-IDPNKRVILFIGDGSLQLTVQEISTMVRWG-LKPYLFVLNN : 471
A.nidulans    : GSIGYSVGACQGAALAAKE-QG-NRRTVLWVGDGSLQLTLQEISTMIRNN-LNPIIFVICN : 477
A.niger       : GSIGYSVGACQGAALAAKE-LG-NRRTVLFVGDGSLQLTVQELSTMIRNN-LNPIIFVICN : 478
A.oryzae      : GSIGYSVGACQGAALAAKE-QG--RRTVLFVGDGSFQLTLQEVSTMIRNN-LNPIIFVICN : 477
B.cinerea     : GSIGYSVGACQGAALAARD-AGSDSRTILFVGDGSFQLTAQEVSTMIRLG-LKPIIFVICN : 478
C.albicans    : GSIGYTVGATCGAAMAAQE-LDPKRRVILFVGDGSLQLTVQEISTMCKWECNNTYLFVLNN : 475
C.glabrata    : GSIGFTTGACLGAAFAAEE-IDPKKRVILFIGDGSLQLTVQEISTMIRWG-LKPYLFVLNN : 471
C.globosum    : GSIGWSVGAAQGAALAAKD-MGTDRRTILFVGDGSFQLTAQEVSTMMRHN-LKVTIFLIYN : 478
C.tropicalis  : GSIGYATGATCGAAMAAQE-IDPKKRVILFTGDGSLQLTVQEISTMCKWDCYNTYLYVLNN : 474
F.oxysporum   : GSIGWSVGACQGAALAAKD-AGKEGRTILFVGDGSFQLTAQELSTMIRHH-LKPTIFVICN : 476
K.africana    : GSIGFTGGAVLGAAFAAEE-IDPKKRVILFIGDGSLQLTVQEISTMIRWN-LKPYLFVLNN : 471
K.lactis      : GSIGFTTGATLGAAFAAEE-IDPKKRVILFIGDGSLQLTVQEISTMIRWG-LKPYLFVLNN : 471
M.acridum     : GSIGWSVGACQGAALAARE-MGEKRRTILFVGDGSIQLTAQELSTMIRHE-LSPIIFVICN : 479
M.gypseum     : GSIGYSLGACQGAALATKE--GASRRTILFIGDGSFQLTVQEISTMIRNG-LTPIIFIICN : 477
M.thermophila : GSIGWSVGAAQGAALAAKD-MGVDRRTILFVGDGSFQLTAQEVSTMIRHD-LRITIFLIFN : 478
N.dairenensis : GSIGFTTGACLGAAFAAEE-VDPKKRVILFIGDGSLQLTVQEISTMVRWN-LKPYLFVLNN : 471
N.tetrasperma : GSIGWSVGAAQGAALAVKD-LEQDRRTILFVGDGSFQLTCQEVSTMLKHN-LRVTMLH--- : 475
P.anserina    : GSIGWSVGAAQGVAQAVKD-MGEDRRTILFVGDGSFQLTVQEVSTMIKHK-LRVTIFLIYN : 480
S.cerevisiae  : GSIGFTTGATLGAAFAAEE-IDPKKRVILFIGDGSLQLTVQEISTMIRWG-LKPYLFVLNN : 539
S.cryophilus  : GSIGYSVGAFFGAALAVKDSSEPDRRTILVVGDGSLHLTATEISTCVRQN-LKPIIFVINN : 477
S.japonicus   : GSIGYSVGAAFGAALGVYDSAEPDRRTILVVGDGSLQLTLTEISTFIRQG-LKPIIFILNN : 479
S.macrospora  : GSIGWSVGATQGAALAVKD-LEQDRRTILFVGDGSFQLTCQEVSTMMKHN-LRVTIFLIYN : 478
S.pombe       : GSIGYSVGAMFGAVLAVHDSKEPDRRTILVVGDGSLQLTITEISTCIRHN-LKPIIFIINN : 477
T.blattae     : GSIGFTGGCVLGAAFAAEE-INPNKRTILFIGDGSLQLTVQEISTLIRWN-LKPYLFVLNN : 471
T.delbrueckii : GSIGFTTGATLGAAFAAEE-IDPKKRVILFIGDGSLQLTVQEISTMVRWG-LKPYLFVLNN : 471
T.terrestris  : GSIGWSVGAAQGAALAARD-MGVDRRTILFVGDGSFQLTAQEVSTMIRHN-LKVTIFLIYN : 479
U.hordei      : GSIGWSVGATLGVALAARE--NKLGRTCLFVGDGSLQLTVQEIGTMIRHG-LTPYLFVLNN : 475
V.polyspora   : GSIGFTGGATLGAAFAAEE-IDPKKRTIVFIGDGSLQLTVQEISTMIRWN-LKPYIFVLNN : 471
Z.rouxii      : GSIGYTTGSTLGAAFAAEE-IDPKKRVILFIGDGSLQLTVQEISTMIRWG-LKPYLFVLNN : 471

N.crassa      : ---------------YRTLH-PWHGRGL--------QRC---------------------- : 490
U.maydis      : DGYEIERQIHGPQRSYNDIP-PYDHSLLLDFFGN--EKF-HSDAKSHENDPHQSKKQYYRV : 534
A.gossypii    : DGYTIERLIHGETAQYNCIQ-PWKHLDLLPTFGA--KDY-----------------ETHRV : 512
```

```
A.nidulans    : EGYTIERFIHGWDESYNDIQ-TWDIKGLPVAFGGK-GRY-----------------KGYKV : 519
A.niger       : NGYTIERYIHGWDESYNDIQ-PWDIEGLPRVFGAK-DKY-----------------KGYKV : 520
A.oryzae      : EGYTIERYIHGWEAVYNDIQ-PWDFLNIPVAFGAK-DKY-----------------KGYKV : 519
B.cinerea     : DGYTIERFIHGMEEEYNDIA-TWNNKDLVPAFGAKEGKY-----------------KLHQI : 521
C.albicans    : DGYTIERLIHGEKAQYNDIQ-PWNNLQLLPLFNA--KDY-----------------ETKRI : 516
C.glabrata    : DGYTIERLIHGEKAGYNDIQ-NWDHLALLPTFGA--KDY-----------------ENHRV : 512
C.globosum    : EGFTIERYIHGMEAEYNDVV-RWQYTDVPTVFGGSDKQV-----------------RKFVI : 521
C.tropicalis  : DGYTIERLIHGEKAQYNDIQ-PWNNLQLLPLFNA--KKY-----------------ETKRI : 515
F.oxysporum   : DGFTIERFIHGMDAVYNDIN-NWKYKDLVSVFGGE-KTC-----------------KTFQI : 518
K.africana    : DGYTIERLIHGEKAGYNDIQ-NWKHLMLLETFGA--KDY-----------------ENHRV : 512
K.lactis      : DGYTIERLIHGETAQYNCIQ-NWQHLELLPTFGA--KDY-----------------EAVRV : 512
M.acridum     : EGFTIERFIHGMDATYNDIA-KWDNRALVDVFGGQ-GKA-----------------QKFAV : 521
M.gypseum     : NGYTIERYIHGWNAAYNDIQ-EWKFKEIPSAFGAQPDQF-----------------TTYQI : 520
M.thermophila : GGFTIERFIHGMEAEYNDIT-RWNYIDVPTAFGGSEKQV-----------------RKFVV : 521
N.dairenensis : NGYTIEKLIHGPTAQYNEIQ-SWDHLSLLPTFGA--KDY-----------------ENHRV : 512
N.tetrasperma : ---------------YRTLH-PW--------YGRGLQRY---------------------- : 490
P.anserina    : EGFTIERCIHGMEAEYNDIR-RWNYTEIPTVFGATDKEV-----------------RKHII : 523
S.cerevisiae  : DGYTIEKLIHGPKAQYNEIQ-GWDHLSLLPTFGA--KDY-----------------ETHRV : 580
S.cryophilus  : DGYTIERLIHGLHAGYNDINTNWQYQDMLKFFGA--KQY-----------------RSYRV : 519
S.japonicus   : NGYTIERLIHGLHAVYNEINTKWGYQELLKFFGA--DQS-----------------RTYKV : 521
S.macrospora  : EGFTIERYIHGMDADYNDII-RWNYTDIPAVFGAKEGHG-----------------QKFVI : 521
S.pombe       : DGYTIERLIHGLHASYNEINTKWGYQQIPKFFGAAENHF-----------------RTYCV : 521
T.blattae     : NGYTIEKLIHGPTAQYNEIQ-GWNHLQILPTFGA--KDY-----------------EALRV : 512
T.delbrueckii : DGYTIERLIHGEKAQYNDIQ-PWKNLDLLPTFGA--KDY-----------------ETHRV : 512
T.terrestris  : EGFTIERFIHGMEAEYNNIA-RWQYTEVPAVFGASDKQC-----------------RKFVV : 522
U.hordei      : DGYEIERQIHGPERKYNDIP-PYDHSLMLDFFGD--QKAGKREGKSHEKDMEPSKKQFYRV : 533
V.polyspora   : DGYTIEKLIHGPTAQYNEIQ-NWKHLDILPTFGA--KDY-----------------EAIRV : 512
Z.rouxii      : DGYTIERLIHGETAEYNCIQ-PWKHLELLNTFGA--KDY-----------------ENHRV : 512

N.crassa      : ------------HPLELYRY------------------------------PRGLWRQGRPV : 509
U.maydis      : QTKQQLDELLKDQSFANPDRIR-----------------LIEVVMQRGDAPEALKRQAEAT : 578
A.gossypii    : ATTGEWRALTEKQEFNKNSSIR-----------------MIEIMLDVMDAPSSLIKQAKLT : 556
A.nidulans    : TTRDELTKLFASEEFSSAPCLQVSLCDLTHYLSDANFEQLVEVHMPREDAPASLKITAEAA : 580
A.niger       : KTRDELRQLFANQEFASAPYLQ-----------------LVELHMPRDDAPAALKITAEAA : 564
A.oryzae      : TTRDELRELFANEEFASAPCLQ-----------------LVELHMPRDDCPASLKLTAESA : 563
B.cinerea     : KTKDQVNELFTNKEFNSADCLQ-----------------FVELYIPKEDAPRALVLTAEAS : 565
C.albicans    : STVGELNDLFADKAFAVPDKIR-----------------MVEVMLPTMDAPANLVAQAKLS : 560
C.glabrata    : ATTGEWDKLTQDKEFNKNSKIR-----------------MIEVMLPVMDAPTSLIEQAKLT : 556
C.globosum    : KTKDELEKLLTDKEFNEAGGLQ-----------------FVELWMPKDDAPRALKITAEIA : 565
C.tropicalis  : STVGELNDLFTNKEFAVPDRIR-----------------MVEIMLPVMDAPANLVAQAKQS : 559
F.oxysporum   : KTKTELNELLTNKEFNAAECLQ-----------------FVELYMPREDAPRALIMTAEAS : 562
K.africana    : ATTGEWDALMGDKEFNKNSRIR-----------------MIEVMLPVMDAPSNLVQQAKIT : 556
K.lactis      : STTGEWNKLTTDEKFQDNTRIR-----------------LIEVMLPTMDAPSNLVKQAQLT : 556
M.acridum     : KTKDELNELLASPSFKAANQLQ-----------------LVEIYMPKKDAPRLLLMTAEAS : 565
M.gypseum     : RERQELLDLFSNKEFCSAKRLQ-----------------IVEVYTPQEDAPSTLRLTAEAA : 564
M.thermophila : KTKDELEELLTDTDFNEARGLQ-----------------FVELWMRKDDAPRALKITAEIA : 565
N.dairenensis : ATTGEWIKLTENKEFNQNSRIR-----------------LIEIMLPVMDAPSNLVAQAQLT : 556
N.tetrasperma : ------------HPLELYRY------------------------------PRGLWRQGRPV : 509
P.anserina    : KTKSELEKLLADKDFNDAKGLQ-----------------LVELWMPKHDAPRALKLTAEQS : 567
S.cerevisiae  : ATTGEWDKLTQDKSFNDNSKIR-----------------MIEIMLPVFDAPQNLVEQAKLT : 624
S.cryophilus  : KTPQELEKLFNDKQFASADVIQ-----------------LVEVIMPMLDAPRILVEQAKLT : 563
S.japonicus   : QTPSDVEKLFKDKQFASADVIQ-----------------LVEVVMPTFDAPRILVEQAKLT : 565
S.macrospora  : KTKDELEELLKDKDFNEYKGLQ-----------------FVELWMPKDDAPRALKLTAEIS : 565
S.pombe       : KTPTDVEKLFSDKEFANADVIQ-----------------VVELVMPMLDAPRVLVEQAKLT : 565
T.blattae     : ATTGEWIKLTTDKAFQKNSKIR-----------------MIEIMLPVMDAPSNLVAQAKLT : 556
T.delbrueckii : STTGEWNKLTTDSEFNKNSKIR-----------------MIEVMLPVMDAPSSLVAQAQLT : 556
T.terrestris  : KTKDQLEKLLTDKEFNEARGLQ-----------------FVELWLPKEDAPRALRITTEVA : 566
U.hordei      : QGRKQLDELLESDEFAKPDRIR-----------------VVELMMERGDAPKALKRQAEAT : 577
V.polyspora   : ATTGEFNKLCEDKAFNENSKIR-----------------MIEIMLPVMDAPSNLVAQAKLT : 556
Z.rouxii      : STVGEWNKLTQDPKFNENSRIR-----------------MIEVMLEVMDAPSSLVAQAQLT : 556

N.crassa      : SEVRYQDKG-- : 518
U.maydis      : GQANKYE---- : 585
A.gossypii    : AATNAQP---- : 563
A.nidulans    : ASRNK------ : 585
A.niger       : ATRNK------ : 569
A.oryzae      : AERNKSL---- : 570
B.cinerea     : AKNNAKD---- : 572
C.albicans    : EKTNAEQ---- : 567
C.glabrata    : ASTNAKQ---- : 563
C.globosum    : AKNNAKMSD-- : 574
C.tropicalis  : AATNAAQE--- : 567
F.oxysporum   : ARNNAKKH--- : 570
```

```
K.africana     : AATNAKQD--- : 564
K.lactis       : AATNAKN---- : 563
M.acridum      : AKTNARKE--- : 573
M.gypseum      : AKRNG------ : 569
M.thermophila  : ARNNASMSE-- : 574
N.dairenensis  : AKINAKQD--- : 564
N.tetrasperma  : SEVRHQDKG-- : 518
P.anserina     : AKNNARME--- : 575
S.cerevisiae   : AATNAKQ---- : 631
S.cryophilus   : AKINQE----- : 569
S.japonicus    : AKINKQ----- : 571
S.macrospora   : AKNNSRNEDEQ : 576
S.pombe        : SKINKQ----- : 571
T.blattae      : AATNAKQ---- : 563
T.delbrueckii  : ASINAKN---- : 563
T.terrestris   : ARTNARVE--- : 574
U.hordei       : GTANKYE---- : 584
V.polyspora    : ASINAKQ---- : 563
Z.rouxii       : AATNAKQ---- : 563
```
