# Supplementary material for: Characterization of indole-3-pyruvic acid pathway-mediated biosynthesis of auxin in Neurospora crassa
Source: PLoS One. 2018 Feb 8;13(2):e0192293. doi: 10.1371/journal.pone.0192293 (PMC5805262; doi:10.1371/journal.pone.0192293)
Supplement: S3 Fig — Amino acid residues with black background yellow font represent 100% sequence identity; 75% identity is shown in black font on a red background, and 50% sequence identity is shown in black font on a green background. NAD serves as cofactor for the enzyme aldehyde dehydrogenase. NAD binding residues are denoted by a pink star the top of the residues. (PDF) [file pone.0192293.s003.pdf]

```
N.crassa            : -------------------------MSSNVFVELKTPVT-GTYKQPTGLFINNEFVEGVDK :  35
U.maydis            : --------------------------MPTLNLDLPNGIK---STIQADLFINNKFVPALDG :  32
A.delicata          : -------------------------MAPTFSYTFDHPSYSGKVTFPAGVYIDGKYSDGAEG :  36
A.flavus            : --------------------------MADLFTTIETPSA--KYEQPLGLFINNEFVKAKSG :  33
A.nidulans          : --------------------------MSDLFTTIETPVI--KYEQPLGLFINNEFVKGVEG :  33
A.oryzae            : MTPDGSRITEPDRDTPFLVYFVSSLTMADLFTTIETPSA--KYEQPLGLFINNEFVKAKSG :  59
B.cinerea           : ----------------------------MTSVQLKTPLT-GEYTQPTGLFINNEWVKGVDG :  32
C.albicans          : ----------------------MFKKALPLVSKLTTPKG-ITYNQPLGLFINNEYVHPKQQ :  38
C.cinerea okayama   : -------------------------MPSTFSYQWDTPAYKGKTEFNTGLFINGQFVDGSNK :  36
C.immitis           : ---------------------------MALFQKIETPSC--TYEQPLGLFINNEFVKGVEG :  32
C.tropicalis        : ----------------------MFNKALPLVAKLTTPKG-LTYNQPLGLFINNEFVQPKSN :  38
F.mediterranea      : -------------------------MPQTFTYEFNTPVFKGKTSFSTGLYIDGKSVDGVDG :  36
F.oxysporum         : --------------------------MAPLTVELSTPVT-GTYQQPIGLFIDGKWTEGVDK :  34
G.graminis          : --------------------------MANLFTELKTPAT-GAFKQPLGLFINNEWVEGSDK :  34
M.larici-populina   : -------------------------MIPEIKIQFPIETNLEPITLKTGLFINNEFVSSSDN :  36
P.brasiliensis      : ---------------------------MALFQQITTPTI--TYEQPLGLFINNEFVKGVEG :  32
P.chrysogenum       : ---------------------------MSLSTTIQTPNV--TYEQPLGLFINNEFVKGVDG :  32
P.nodorum           : --------------------------MSGLSVKLKTPQT-GEYDQPTGLFINNEWVKAVDG :  34
P.teres             : --------------------------MSETSVKLKTPQT-GEYEQPTGLFINNEWVKSVDG :  34
S.commune           : --------------------------MTSFSYEFKTPAYTGKVNFPTGVFIDGKFSEGSGK :  35
S.japonicus         : ------------------MPAATLREIPAVKIELPNGLT---VDQPVGLFINNKFVDSAAG :  40
S.pombe             : ---------------------MSTKLVDHVEITVPTGKT---YIQPVGLFINNQHVDSVHG :  37
S.reilianum         : --------------------------MPTLNLDLPTGIK---STIQADLFINNKFVPALDG :  32
U.hordei            : --------------------------MPTLNLDLPNGIK---STVQAELFINNKFVPALDG :  32
V.alboatrum         : ---------------------------MGLSAELKTPVT-GTYTQPTGLFINNEWVEGVDK :  33


N.crassa            : KTFEVINPATEEVICSVHEATEKDVDIAVAAARKAF--EGVWRDVTPQQ-RGIYLLKLADL :  93
U.maydis            : KTFATINPSTGKEIGQVAEASAKDVDLAVKAAREAF--ETTWGENTPGDARGRLLIKLAEL :  91
A.delicata          : KLIDVLNPTNGKLVGQIAEGSRADVDRAVEAAHRAF--NNSWGLKVPGFERGKLLIRLAEL :  95
A.flavus            : RTFETINPTNEKPIVAVQEADENDVEDAVKAARAAF--EGEWSKVTPSE-RGRLLVKLADL :  91
A.nidulans          : KTFQVINPSNEKVITSVHEATEKDVDVAVAAARAAF--EGPWRQVTPSE-RGILINKLADL :  91
A.oryzae            : RTFETINPTNEKPIVAVQEADENDVEDAVKAARAAF--EGEWSKVTPSE-RGRLLVKLADL : 117
B.cinerea           : KTFEVINPTTEEVITSVHEATEKDVDIAVAAARKAY--EGEWRHVTPEG-RGKLLVKLAEL :  90
C.albicans          : KTFEVISPSTEEKITDVYEALEEDIDTAVEAAQAAY--HNGWAQGPPEQ-RSKVLFKLADL :  96
C.cinerea okayama   : TTIDVVNPTNGKVITSISEGVEKDVDLAVEAAQKAF--ETTWGLNVSGSQRSELLWKLATL :  95
C.immitis           : KTFETINPHNEKPIVAVHEATEKDVDIAVAAARKAF--EGEWKKVTPTD-RGRMLNKLADL :  90
C.tropicalis        : KTFEVYSPSTGEKITDVYESLAEDVDIAVDAAKTAY--DNGWASGAPEQ-RAKILFKLADL :  96
F.mediterranea      : LTYDVINPTTGKVTTKMCIGNKKDIDLAVKAAHKAY--NTVWGLNCPGYERGKLLMKLATL :  95
F.oxysporum         : QKFEVINPSTEEVITSVCEGTEKDIDLAVTAARKAF--EGEWKSTSPQA-RGNYLLKLADL :  92
G.graminis          : KTFEVINPTTEEVICSVHEATEKDVDLAVKAARKAF--EGEWKQTTPQQ-RGILLNKLADL :  92
M.larici-populina   : KSIETINPSTGKPIGKVSIASKEDVDLAVKSAQEAF--ENHWGTKTPAQKRGRMLMDLADA :  95
P.brasiliensis      : RTFESINPHNEKPIVAVYEATEKDVDIAVAAARAAF--KGPWKHVTPTN-RGRMLLKLADL :  90
P.chrysogenum       : KTFETLNPTTEKPITSVYEASEKDVDIAVKAAREAF--EGPWKKVTPSD-RGRMLTKLADL :  90
P.nodorum           : KTFDVINPSTEEVICQVQEASEKDVDIAVAAARKAF--NGPWRKETPEN-RGRLLVKLADL :  92
P.teres             : KTIEVINPSTEEVICHVAEATEKDVDIAVAAARKAF--NGPWRKETPEN-RGRLLVKLADL :  92
S.commune           : TTIDVINPTTGKLITSVAEATAADVDKAVIAAQKAF--DTTWGLNAAGSRRSELLYKLALL :  94
S.japonicus         : GRIEVIDPATEKHLCDVVEAREEDIDVAVAAARAAFHPDAPWRKFSSAQ-RGRCLSLLADK : 100
S.pombe             : GRVKVYSPSTEKLICEVADADEEDVDIAVKVARAAFQTDAPWRKFSSAQ-RGRCLSRLADC :  97
S.reilianum         : KTFATINPATGKEIGQVAEASAKDVDIAVKAAREAF--ETTWGENTPADARGRLLIKLAEL :  91
U.hordei            : KTFATVNPSTGKEIGQVAEASAKDVDVAVKAAREAF--ETVWGENTPGDARGRLLIRLAEL :  91
V.alboatrum         : KTFEVVNPSTEEVIVSVSEATEKDVDIAVAAARKAF--NGVWRQTTPGQ-RSIYLLKLAEL :  91


N.crassa            : LEKNLDLLAAVESLDNGKSITMARG-DVGAVVGTIRYYGGWADKIEGKTIDISPDSFHYTR : 153
```

```
U.maydis            : VEANIDELAAIESLDNGKAFSIAKSFDVAAVAANLRYYGGWADKNHGKVMEVDTKRLNYTR : 152
A.delicata          : IEEHADHLAAIEALDNGKAFAIARGFDIVESAACFRYYGGWADKLHGKVIETNPTKFSYTQ : 156
A.flavus            : FERDSDILAAIEALDNGKAFTMAKG-DVAAAAGCLRYYGGWADKIHGQTIDTNPESLTYTR : 151
A.nidulans          : MERDIDTLAAIESLDNGKAFTMAKV-DLANSIGCLRYYAGWADKIHGQTIDTNPETLTYTR : 151
A.oryzae            : FERDSDILAAIEALDNGKAFTMAKG-DVAAAAGCLRYYGGWADKIHGQTIDTNPESLTYTR : 177
B.cinerea           : VEKNADLLAAVESLDNGKAISMAKG-DVAAVAGCLRYYGGWADKIEGKVIDTDPEYFTYTK : 150
C.albicans          : IEENAELLAQIETWDNGKSLQNARG-DVALTAAYFRSCGGWADKILGSQINTGNTHFNYTQ : 156
C.cinerea okayama   : MEQHHEELAAIEATDNGKTFAWAKGTDVAFSINVIKYFAGWADKITGQTIETDERKLIYTR : 156
C.immitis           : LEQHCDTLAAIEALDNGKAVSMAKV-DVANSAGCIRYYGGWADKLHGKVVDTDHETFNYTR : 150
C.tropicalis        : VEENLETLAQIETWDNGKSLQNARG-DIGFTAAYFRSCGGWADKINGSQINTGSNYLTYTQ : 156
F.mediterranea      : MEEHHDELSALEALDNGKTFSWARNVDLPGSIQCIRYYGGWADKNHGKVIETDEGKLAYTR : 156
F.oxysporum         : AEKNLDLLAAVESLDNGKSITNARG-DVGAVVGCLRYYGGWADKIEGKTIDIAPDMFHYTR : 152
G.graminis          : VEKNLDLLAAVESLDNGKAFAMAKG-DVGAVVGCLRYYGGWADKIEGKTIDIAPDMFHYTR : 152
M.larici-populina   : IEENLDVLAAIESIDNGKAFSIAKSFDVPEAAHCLRYYGGWADKNHGKVIEVNDSKMAFTR : 156
P.brasiliensis      : MEQHVDTLAAIEALDNGKAYSIARI-DVANAAGCIRYYGGWADKIHGKVIDTDTDSFNYTR : 150
P.chrysogenum       : FERDMETLASIEALDNGKAVTMAKV-DIANAAGCLRYYGGWADKITGQTIDTNSETLSYTR : 150
P.nodorum           : FEKNADLLASVEALDNGKAFNMAKNVDVPSCVGCLRYYGGWADKIEGKVVDTTPDTFNYIR : 153
P.teres             : FEKNADLIAAVEALDNGKAFSMARNVDVPAAAGCLRYYGGWADKIEGKVVDTSPDTFNYIR : 153
S.commune           : MEQNLDELCALEALDNGKTFGWAKNVDVAFAIQTIKYFAGWADKIHGKVIETDESKLTYTR : 155
S.japonicus         : VYEHLELLASVETVDNGKSITLARG-DVRSAAECIRYYGGWADKEYGQTIETDTTRFAYTR : 160
S.pombe             : IEQNLEYLASIETLDNGKSITLARG-DVQAAADCFRYYGGWADKDYGQTIETDIKRFAYTR : 157
S.reilianum         : VEANVDELAAIESLDNGKAFSIAKGFDVAAVAANLRYYGGWADKNHGKVMEVDTSRLNYTR : 152
U.hordei            : VEEHVDELAAIESLDNGKAFSIAKGFDVSAAAANLRYYGGWADKNHGKVIEVDNKRLNYTR : 152
V.alboatrum         : VEKNIDLLAAVESLDNGKSITMAKG-DVGAVVGCIRYYGGWADKIEGKTIDVAPDMFHYTR : 151
```

```
                                         *
N.crassa            : QEPLGVCCQIIPWNFPLLMLAWKVGPALATGNTIVMKTAEQTPLSALVFAQFVKEAGFPPG : 214
U.maydis            : HEPIGVCGQIIPWNFPLLMFAWKLGPALATGNTIVLKTAEQTPLSAIKMCELIVEAGFPPG : 213
A.delicata          : HEPIGVVGQIIPWNFPFMMAAWKLAPALATGNTIVLKPSEFTPLSVLYLCQLFSEAGFPPG : 217
A.flavus            : HEPIGVCGQIIPWNFPLLMWSWKIGPAIAAGNVVVLKTAEQTPLSGLYAAKLIKEAGFPAG : 212
A.nidulans          : HEPVGVCGQIIPWNFPLLMWSWKIGPAVAAGNTVVLKTAEQTPLSALYAAKLIKEAGFPAG : 212
A.oryzae            : HEPIGVCGQIIPWNFPLLMWSWKIGPAIAAGNVVVLKTAEQTPLSGLYAAKLIKEAGFPAG : 238
B.cinerea           : QEPIGVCGQIIPWNFPLLMWSWKIGPAVATGNTVVLKTAEQTPLSALVAANLIKEAGFPPG : 211
C.albicans          : RVPL-VCGQIIPWNFPLLMASWKLGPVLATGSTTVLKTAESTPLSALYLSQLLVEAGMPKG : 216
C.cinerea okayama   : HEPIGVVGQIIPWNFPLLMLAWKIGPALATGNTIVLKPSEFTPLTAIRMCSLIQEAGFPPG : 217
C.immitis           : HEAVGVCGQIIPWNFPLLMWAWKIAPALATGNTIVLKSAEQTPLSALYACQLVKEAGFPPG : 211
C.tropicalis        : RVPL-ISGQIIPWNASTLMASWKLGPVIATGGTTVLKPAEHTPLAVLYLAQLLVEAGMPKG : 216
F.mediterranea      : HEPIGVVGQIIPWNFPLLMLAWKIGPALATGNAIVLKPSEFTPLTALRMVGLIEEAGFPPG : 217
F.oxysporum         : SEPIGVCGQIIPWNFPLLMLAWKIGPALATGNTVVMKTAEQTPLSALVFTQFIEQAGFPAG : 213
G.graminis          : QEPIGVCGQIIPWNFPLLMFAWKIGPALATGNTVVLKTAEQTPLSGLVMAQFVKEAGFPAG : 213
M.larici-populina   : HEPIGVVGQIIPWNFPLLMFAWKIGPALSTGNTIVIKPAEQTPLTALFMAQLISKI-FPPG : 216
P.brasiliensis      : HEPIGVCGQIIPWNFPLLMWSWKIGPVVATGNTVVLKSAEQTPLSALYAAKLVVEAGFPPG : 211
P.chrysogenum       : HEPVGVCGQIIPWNFPILMWAWKIGPAIAAGNTVLIKTAEQTPLSGLYASKLIVEAGFPPG : 211
P.nodorum           : KEPVGVCAQIIPWNFPLLMWAWKIGPAIATGNCVVIKTAEQTPLSAYIAANLIKEAGFPPG : 214
P.teres             : KEPIGVCGQIIPWNFPILMWAWKIGPAIATGNTVVMKTAEQTPLSGYIAAKLIVEAGFPPG : 214
S.commune           : HEPIGVVGQIIPWNFPLLMFAWKIGPALATGNTVVLKPSEFTPLTALRTCTLINEAGFPPG : 216
S.japonicus         : HEPIGVCAQVIPWNFPILMFAWKIAPALACGNTVVLKTAEQTPLSALMLTSFIEECGFPAG : 221
S.pombe             : HEPIGVCGQIIPWNFPFLMCAWKIAPAVACGNTIILKTAELTPLSALCLTKFVPECGFPPG : 218
S.reilianum         : HEPIGVCGQIIPWNFPLLMFAWKIGPALATGNTVVLKTAEQTPLSAIKFCELIVEAGFPPG : 213
U.hordei            : HEPIGVCGQIIPWNFPLLMFAWKIGPALATGNTIVLKTAEQTPLSAIKLCELIVKAGFPPG : 213
V.alboatrum         : QEPIGVCGQIIPWNFPILMLAWKIAPALATGNTVVMKTAEQTPLSGLVFANLIKEAGFPPG : 212
```

```
                                                    **                      ***
```

```
                                             *
N.crassa           : VLNIISGFGRIAGAAMASHMDIDKVAFTGSTMVGRQIMKAAAESNLKKVTLELGGKSPNII : 275
U.maydis           : VVNVISGFGPVAGAAISQHMDIDKIAFTGSTLVGRNIMKAAASTNLKKVTLELGGKSPNII : 274
A.delicata         : VVNVVNGYGNVVGAAISEHMHIEKVAFTGSTIVGRAVMKAAASSNLKKVTLELGGKSPNII : 278
A.flavus           : VVNILSGFGRVAGAAISSHMDIDKIAFTGSTLVGRMILQAAAKSNLKKVTLELGGKSPNIV : 273
A.nidulans         : VINVISGFGRTAGAAISSHMDIDKVAFTGSTLVGRTILQAAAKSNLKKVTLELGGKSPNIV : 273
A.oryzae           : VVNILSGFGRVAGAAISSHMDIDKIAFTGSTLVGRMILQAAAKSNLKKVTLELGGKSPNIV : 299
B.cinerea          : VVNIISGFGKIAGAAIASHMDVDKVAFTGSTVVGRQIMKAAAGSNLKKVTLELGGKSPNIV : 272
C.albicans         : VINIVSGFGATAGAAIAKHPKIEKVAFTGSTATGKIIMKLAAESNLKKVTLELGGKSPNIV : 277
C.cinerea okayama  : VVNIVTGYGNTVGAAISSHPKIEKVAFTGSTLVGRKIMEAAAKSNLKNVTLELGGKSPNVI : 278
C.immitis          : VLNVISGFGRVAGAAISSHMDIDKVAFTGSTLVGRQILQAAAKSNLKKVTLELGGKSPNIV : 272
C.tropicalis       : VVNIVPGFGATTGAAIASHPNIDKVAFTGSTATGKVIMKLAADSNLKKVTLELGGKSPNIV : 277
F.mediterranea     : VVNIINGLGPDAGAAIAEHPDIEKVAFTGSTLVGRRIMEAAAKTNLKKVTLELGGKSPNII : 278
F.oxysporum        : VFNLVSGFGKTAGAALSAHMDVDKIAFTGSTVIGRQIMKSAASSNLKKVTLELGGKSPNIV : 274
G.graminis         : VLNVVSGFGKIAGAAISAHMDVDKVAFTGSTVVGRTIMKAAASSNLKKVTLELGGKSPNIV : 274
M.larici-populina  : VINIIVGLGPVTGVAMSHHMGIEKIAFTGSTVVGKMIMKAAAESNLKKVTLELGGKSPTII : 277
P.brasiliensis     : VINIISGFGRVAGAAISSHMDIDKVAFTGSTLVGRQILQAAAKSNLKKVTLELGGKSPNIV : 272
P.chrysogenum      : VINVISGFGRVAGAAISSHMDIDKVAFTGSTLVGRMILQAAAKSNLKKVTLELGGKSPNIV : 272
P.nodorum          : VINVITGFGKIAGAALSAHMDVDKIAFTGSTVVGRQIMKSAAGSNLKKVTLELGGKSPNIV : 275
P.teres            : VVNIITGLGRVAGAAMSAHMDIDKIAFTGSTVVGRQIMKAAAGSNLKKITLELGGKSPNIV : 275
S.commune          : VVNLIVGYGHTVGAAMSSHMDIHKLAFTGSTLVGRKIMESAAKSNLKTVTLELGGKSPNII : 277
S.japonicus        : VINVVSGYGKHAGAALSAHMDVDKIAFTGSTGVGRMIMRAAAASNLKMCTLELGGKSPNIV : 282
S.pombe            : VINVLSGDGRRCGNAISSHMDIDKVAFTGSTGVGRMVMRAAASSNLKKVTLELGGKSPNIV : 279
S.reilianum        : VVNVISGFGPVAGAAISQHMDIDKIAFTGSTLVGRNIMKAAASTNLKKVTLELGGKSPNII : 274
U.hordei           : VVNVISGFGPVAGAAISQHMDIDKIAFTGSTLVGRNIMKAAASTNLKKVTLELGGKSPNIV : 274
V.alboatrum        : VFNLINGFGKVAGAAISSHMDIDKVAFTGSTLVGRQIMKAAAASNLKKVTLELGGKSPNIV : 273
```

```
                                             *
N.crassa           : FNDADIDQAIDWVNFGIYFNHGQTCCAGSRVYVQEGIYDKFVAAFKQRAQQNKVGDPFHDE : 336
U.maydis           : FKDADLDQAVRWSAFGIMFNHGQCCCAGSRVYVEESIYDAFMEKMTAHCKALQVGDPFSAN : 335
A.delicata         : FEDADVDQAIRWAAFGLFFNHGQCCCAGSRVYVQESIYDSFVEKFKAHVKTLKVGDPFDKE : 339
A.flavus           : FDDADIDNAISWSNFGIFFNHGQCCCAGSRILVQEGIHDKFVARFKERAAANKLGNPFTAD : 334
A.nidulans         : FDDADIDNAISWANFGIFFNHGQCCCAGSRILVQEGIYDKFVARFKERAQKNKVGNPFEQD : 334
A.oryzae           : FDDADIDNAISWSNFGIFFNHGQCCCAGSRILVQEGIHDKFVARFKERAAANKLGNPFTAD : 360
B.cinerea          : FNDADIENAISWVNFGIFFNHGQTCCAGSRVYVQDGIYDKFVESFKARAIANKVGDPFHHE : 333
C.albicans         : FNDADLDKTIQNLIVSIFYNSGEVCCAGSRLLIQSGVYDQVVEKFKEAAESVKVGNPFDED : 338
C.cinerea okayama  : FNDADIDQAVNWAVHGLFWNHGQACCAGSRIFVQSGIYDEFLKRFTAKAAAIRVGDPFGVE : 339
C.immitis          : FNDADIDNAISWVNFGIYFNHGQCCCAGSRILVQEGIYEDFLQRFKERAMKNKVGDPFNPD : 333
C.tropicalis       : FNDADLDKTVHNLVSGVFSNSGETCAAGARVLVQSGVYDEVVARFKKAAEAIKVGDPFEEE : 338
F.mediterranea     : FDDADIQQAVDWAAHGVFWNHGQACCAGTRIFVQEKIYDEFLQKFTAKAQGLKIGDPFGVD : 339
F.oxysporum        : FDDADIEEAINWVNFGIYYNHGQCCCAGTRIFVQEGIYDKFLAAFKKRAEENKVGDPFNEE : 335
G.graminis         : FNDADIEQAVSWVNFGIYFNHGQCCCAGSRVYVQEDIYDKFVAAFKARAEKNAVGDPFKDD : 335
M.larici-populina  : FDDADLDQAVKWSGFGIFFNHGQCCCAGSRVFVHEAVYDQFMVKFEEYAKSFKVGDPFSKN : 338
P.brasiliensis     : FNDADIDNAISWVNFGIYFNHGQCCCAGSRILVEEGIYDTFLERFKARALQNKVGDPFHQD : 333
P.chrysogenum      : FEDADIDNAISWANFGIFFNHGQCCCAGSRLLVQESIHDKFVARFKERAAQNKLGNPFEGD : 333
P.nodorum          : FADADIDEAINWVNFGIYFNHGQCCCAGSRIYVQEEIYDKFIARFRERAAQNAVGDPFSKD : 336
P.teres            : FADADLDEAINWVNFGIYFNHGQTCCAGSRIYVEESIYDKFIERFRERAAQNKVGDPFAAE : 336
S.commune          : FDDADLEQAVNWAAHGIFWNHGQACCAGSRIFVQAGIYDKFLEAFTARTRGIKVGDPFGDN : 338
S.japonicus        : FDDADLDKAVAWTNYGIFYNQGQVCCAGSRLYVQEGIYDEFVKRMVAKAKTLKVGNPFDED : 343
S.pombe            : FNDADLDSAAVWTNYGIFYNSGQVCCAGSRVYVQEDVYDEFIKRMVAKAKTLKVGDPFAED : 340
S.reilianum        : FKDADLDQAVRWSAFGIMFNHGQCCCAGSRVYVEDSIYDAFMEKMTAYCKALQVGDPFAAT : 335
U.hordei           : FKDADLDQAVKWSAFGIMFNHGQCCCAGSRIYVEEEIYDAFMEKMTAHCKALPVGDPFAAN : 335
V.alboatrum        : FNDADIEQAISWVNFGIYYNHGQCCCAGTRIYVQEGIYDKFLEAFKKRAQENKVGDPFHDE : 334
```

```
                                                                                 *
```

```
N.crassa            : TFQGPQVSQLQYDRIMGYIKAGKEEGATVETGGERHGDKGYFIQPTIFTNVRHDMKIMKEE : 397
U.maydis            : TFQGPQVSQLQYDRIMEYIESGKKD-ANLALGGVRKGNEGYFIEPTIFTDVPHDAKIAKEE : 395
A.delicata          : TFQGPQVSQLQFDRIMGYIESGKKAGATVELGGKRHGNEGYFIEPTVFTNVKPDMQIVREE : 400
A.flavus            : TFQGPQVSQLQFDRIMEYINHGKQEGATVATGGERHGTEGYFIQPTVFTDVHSDMKIAKEE : 395
A.nidulans          : TFQGPQVSQLQFDRIMEYINHGKKAGATVATGGDRHGNEGYFIQPTVFTDVTSDMKIAQEE : 395
A.oryzae            : TFQGPQVSQLQFDRIMEYINHGKQEGATVATGGERHGTEGYFIQPTVFTDVHSDMKIAKEE : 421
B.cinerea           : TFQGPQVSQLQYDRIMGYIDEGKKSGATVVTGGERHGDKGYFIQPTIFSDVTEDMKIMQEE : 394
C.albicans          : TFMGAQVSDVQLSKILKYVESGKSQGATVVTGGARADGKGYFVKPTIFADVKKDMDIVREE : 399
C.cinerea okayama   : VDQGPQVSQIQYDRIMGYIDSGKADGATVHLGGKRHGQEGYFIEPTIFTDVKPDMKIVREE : 400
C.immitis           : TFQGPQISQLQFDRVMGYIDQGKKAGAKVEIGGERLGTEGYYIQPTIFSNVNEDMSIVKEE : 394
C.tropicalis        : TFMGSQVNEIQFSKILEFVESGKEQGATLVTGGERIGDKGYFIKPTIFADVKKDMTIFKEE : 399
F.mediterranea      : TYQGPQVSQPQFDRIMGYIESGKQEGATIHTGGSRFGKEGYFIEPTIFTNTRPDMKIVREE : 400
F.oxysporum         : TFQGPQVSQLQYDRIMGYIKAGKDEGATIETGGERLGNKGYFIKPTIFSNVRPDMKIMQEE : 396
G.graminis          : TFQGPQVSQLQYDRIMEYIKAGKDEGATVETGGGRHGDKGYFIQPTIFTNVRNDMKIMQEE : 396
M.larici-populina   : TFQGPLVSQLQFDRVMGYIQSGKEDGAKCIIGGNRYGNEGYFIEPTIFTDVKPSMKIMKEE : 399
P.brasiliensis      : TFQGPQVSQLQFDRIMGYIREGKAAGAKVEIGGERLGNEGYYIQPTIFSNVTEDMKIVKEE : 394
P.chrysogenum       : TFQGPQVSQLQFDRIMEYINHGKTEGATVALGGERHGTEGYFIQPTVFTDVTPDMKIAQEE : 394
P.nodorum           : TFQGPQVSQLQFDRIMSYIEEGKKSGATIETGGKRKGDKGYFIEPTIFSNVTEDMTIQQEE : 397
P.teres             : TFQGPQVSQLQFDRIMGYIDEGKKEGATIETGGKRKGDKGYFIEPTIFSNVTEDMKIQKEE : 397
S.commune           : VDQGPQVSQQQYDRIMSYIDTGKQEGATCHIGGNRVGDEGYFIEPTIFTDVKPDMTIVREE : 399
S.japonicus         : TFQGAQVSKAQYERILSYIDLGLEHGAKLEIGGKRHGDRGYFIEPTILSNVTEEMAVGKEE : 404
S.pombe             : TFQGAQVSKQQYERIVSYIESGIAHGAKLEIGGKRHGNLGYFVEPTILSNVTEDMAVGKEE : 401
S.reilianum         : TFQGPQVSQLQYDRIMEYIETGKKE-ANLHLGGVRKGEQGYFIEPTIFTDAPHESKIAKEE : 395
U.hordei            : TFQGPQVSQLQYDRIMEYIESGKKD-ANLHLGGVRKGNEGYFIEPTIFTDVPHDAKIAKEE : 395
V.alboatrum         : TFQGPQVSQLQFDRIMEYIKIGKEEGATVETGGERHGDKGYFIQPTIFSNVKADMKIMQEE : 395
```

```
                        *
N.crassa            : IFGPVCAVAKFSTEEEVIKLGNDSNYGLAAAVHTKDLNTAIRVSNHLRAGTVWVNTYNALH : 458
U.maydis            : IFGPVVVVSKFKDEKDLIRIANDSIYGLAAAVFSRDISRAIETAHKLKAGTVWVNCYNQLI : 456
A.delicata          : IFGPVVVLAKFKDEDDIIAQANDSMYGLAAAVFSQNVSRALSVAQRLHAGTVWVNHYNKLH : 461
A.flavus            : IFGPVVTIQKFKDEEEAIKIGNSSSYGLAAAVHTKNVNTAIRVSNSLRAGTVWINCYNMIN : 456
A.nidulans          : IFGPVVTIQKFKDEAEAIKIGNSTDYGLAAAVHTKNVNTAIRVSNALKAGTVWINNYNMIS : 456
A.oryzae            : IFGPVVTIQKFKDEEEAIKIGNSSSYGLAAAVHTKNVNTAIRVSNSLRAGTVWINCYNMIN : 482
B.cinerea           : IFGPVCSIAKFSTEEEIIKIGNGSNYGLASAVHTQNLNTALRVSNALKAGTVWVNCYNMLH : 455
C.albicans          : IFGPVVTLIKFDTVDEAVELANDSDYGLAAGIHSADVNKCIDVANRVKAGTVWVNTYNDFH : 460
C.cinerea okayama   : IFGPVGVLIKFEDEADVIRQANDTVYGLAAAVFSQNINRAIETAHKMQAGTVWVNCANQLH : 461
C.immitis           : IFGPVCSIQTFKTEEDAIKIANGTSYGLAAAIHTKDLNTAIRVSNEIRAGTVWVNCYNLLS : 455
C.tropicalis        : IFGPVASVIKFDTIDEAIALANDSEYGLAAGVHTTNINTGLHVANNIKSGSVWVNTYSELH : 460
F.mediterranea      : IFGPVGVVIKFHDEEDVIRQANDTHYGLAAAVFSKNIDRALRVAHRLHAGTAWINCANTLN : 461
F.oxysporum         : IFGPVCAISKFKDEKEVIDLAHDTAYGLAAAVHTKNLNTALRVSNALKAGTVWVNCYNMLH : 457
G.graminis          : IFGPVCAIAKFKTEEDVIHMGNDSSYGLAAAVHTKDLNTAIRVSNSLKAGTVWVNCYNMLH : 457
M.larici-populina   : IFGPVVAVTKFSSEEDLLKVANGSIYGLAAAVFSKDIQRSIKVANELKAGTVWVNCYNKLH : 460
P.brasiliensis      : IFGPVCCVQKFKNEDEAICIANNTSYGLAAAVHTTDLNTAIRVSNELKAGTVWVNNYNMLS : 455
P.chrysogenum       : IFGPVIAVTKFKDEADAIRIGNSTSYGLAAAVHTKNINTAIRVSNALKAGTVWINNYNMIS : 455
P.nodorum           : IFGPVCTISKFKTKADVIKIGNSTTYGLAAAVHTTNLNTAIEVANALRAGTVWVNTYNALH : 458
P.teres             : IFGPVCTIAKFKTKEEVIRIGNASIYGLAAAVHTTNLNTAIEVANALRAGTVWVNTYNSLH : 458
S.commune           : IFGPVGVVIKFTDEEDVIRQANDTVYGLAAAVFTQDINRAIETAHKLQAGTAWINCYNQLH : 460
S.japonicus         : IFGPVVAIIKFKTIEEAIRRANNTSFGLASGVHTRSIDTALQVSNALQAGTVWVNCYNVLH : 465
S.pombe             : IFGPVLAVIKFKTIEEAIRRGNNSTYGLAAGVHTNNITNAIKVSNALEAGTVWVNCYNLLH : 462
S.reilianum         : IFGPVVVVSKFKDEKDLIRIANDSIYGLAAAVFSRDISRAIETAHKLKAGTVWVNCYNQLV : 456
U.hordei            : IFGPVVVVSKFKDEKDLIRIANDSIYGLAAAVFSRDISRALETAHKLKAGTVWVNNYNQLN : 456
V.alboatrum         : IFGPVCAIAKFKDEDEVIALGNDSTYGLAAAVHTKDLNTAIRVSNQLKAGTVWVNCYNMLH : 456
```

```
                             *
```

```
N.crassa          : HQLPFGGYKESGIGRELGEAALANYTQCKSVAIKLN----- : 494
U.maydis          : PQVPFGGYKASGIGRELGEYALSNYTNIKAVHVNLSQPAPI : 497
A.delicata        : PQVPFGGFKQSGIGRELGKYALANYTNVKAVQVNIHDKL-- : 500
A.flavus          : YQAPFGGFKESGLGRELGSYALENYTQVKTVHYRLGDALFA : 497
A.nidulans        : YQAPFGGFKQSGLGRELGSYALENYTQIKTVHYRLGDALFA : 497
A.oryzae          : YQAPFGGFKESGLGRELGSYALENYTQVKTVHYRLGDALFA : 523
B.cinerea         : HQVPFGGYKESGIGRELGEAALSNYTQTKSVRIRLGDALFG : 496
C.albicans        : PMVPFGGFSASGIGREMGEEVLHEYTQVRAVRMKINPPN-- : 499
C.cinerea okayama : ANVPFGGFKQSGIGRELGEYALHNYTNVKAVHVNIGHVM-- : 500
C.immitis         : YQTPFGGFKESGLGRELGEYALDNYTQVKAVRIRLGDAMFG : 496
C.tropicalis      : PMVPFGGFKSSGIGREMGEESFKEYTEVRSVRVKLYPDA-- : 499
F.mediterranea    : TQIPFGGYKQSGIGRELGEYALANYSAIKAVHVNIGMSI-- : 500
F.oxysporum       : HQLPFGGYKESGIGRELGEAALANYTQNKSVAIKLY----- : 493
G.graminis        : HQLPFGGFKESGIGRELGEAALANYTQTKSVAIRLGGALF- : 497
M.larici-populina : TQVPFGGFKQSGIGRELGEYALANYTAVKAVHINLGEKL-- : 499
P.brasiliensis    : YQTPFGGFKESGLGRELGEYALDNYTQVKAVRIRLGDALFG : 496
P.chrysogenum     : YQAPFGGFKESGIGRELGSYALENYTQVKTVHYRLGDALF- : 495
P.nodorum         : WALPFGGYKQSGIGRELGEAALDNYLQTKTVSIRLGDVLFG : 499
P.teres           : WQLPFGGYKESGMGRELGEAALDNYLQTKTVSIRLGDVIFG : 499
S.commune         : AQVPFGGFKQSGIGRELGEYALENYTNVKAVHVNLRHRM-- : 499
S.japonicus       : HQIPFGGYKESGIGRELGSYGLSNYTQTKAVHINTGMNLPL : 506
S.pombe           : HQIPFGGYKESGIGRELGSYGLTNYTQTKAVHINLGMDSPI : 503
S.reilianum       : PQVPFGGYKASGIGRELGEYALTNYTNIKAVHVNLSQPAPI : 497
U.hordei          : PQVPFGGYKASGIGRELGEYALANYTNVKAVHVNLSVPAPI : 497
V.alboatrum       : HQVPFGGFKESGIGRELGEAALSNYTQNKSVAIRLGGAIY- : 496
```
