# Supplementary material for: Characterization of indole-3-pyruvic acid pathway-mediated biosynthesis of auxin in Neurospora crassa
Source: PLoS One. 2018 Feb 8;13(2):e0192293. doi: 10.1371/journal.pone.0192293 (PMC5805262; doi:10.1371/journal.pone.0192293)
Supplement: S4 Fig — MOX-2 or flavin monooxygenase is known to bind FAD through its nucleotide binding motif (GXGXXG). The GXGXXG motif is indicated by a bar, and conserved residues are highlighted with pink stars. “X” represents any amino acid. Residues in yellow font on a black background are 100% identical, and residues in black font on a red background are 75% identical. (the MOX-2 homolog in U. mydis was not found using the same BLAST search with the same template used for the other homologs.) (PDF) [file pone.0192293.s004.pdf]

```
                                                                                   *  *   *
                                                                                   ======
N.crassa                  : ---------------------------------MGSQERRFNVKKIAIVGAGPAGL : 23
A.nidulans                : ------------------------------------MALSKHIHRIAVIGAGPAGL : 20
A.terreus                 : ------------------------------------MAMSREIRRVAIIGAGPAGL : 20
B.dermatitidis            : ----------------------------------MRALSSHPIRRVAIIGAGPSGL : 22
C.albicans                : --------------------------------MTKEQIDEPRYKRIAVIGGGPTGL : 24
C.cinerea okayama         : MKGLFVYLAVLVSSGVCTQQEPLQWGSSSSPRPQESYEFKWPIKKVAVIGTGVGGL : 56
C.globosum                : --------------------------------MGSQQPERFDVKKVAIIGAGPCGL : 24
C.guilliermondii          : ----------------------------------------MIPKSIAVIGAGPSGA : 16
C.immitis                 : -----------------------------------MAQRFPHVRTVAIVGAGAGGL : 21
C.neoformans grubii       : -----------------------------MCSEEQFHHFNRSVRNVAIIGSGPSGT : 27
C.tropicalis              : -----------------------------------MTKQQPLYDRVAIIGGGPTGL : 21
F.graminearum             : ----------------------------------MALADKLDVRRIAIIGAGPSGL : 22
F.oxysporum               : ----------------------------------MALADKLDVRRIAIIGAGPSGL : 22
F.verticillioides         : -------------------------------MGSLTQPTPFDVHKIAIIGAGPTGL : 25
H.capsulatum              : ----------------------------------MRGISSPRIRRVAIIGAGPSGL : 22
L.elongisporus            : --------------------------------MTVSVLKQPLYNRVAIIGGGPAGL : 24
M.canis                   : -----------------------------------MTRITPPVRRVAIIGAGACGL : 21
M.gypseum                 : -----------------------------------MTRATPPVRKVAIIGAGACGL : 21
M.oryzae                  : ----------------------------------MVLRACFDVKRIAVIGAGPCGL : 22
P.graminis tritici        : -----------------------------------MHENSLDETDVVIIGAGASGL : 21
P.nodorum                 : -------------------------------MTSEMTPLNFKALSIAIVGAGPSGV : 25
P.triticina               : ---------------------------MMGNQRTRKRTRMEEETDVVVIGAGASGL : 29
P.tritici-repentis        : --------------------------------MTENERISIRARTVAVVGAGPSGV : 24
Schizosaccharomyces pombe : -----------------------------------MSLCLPTIRKIAIIGAGPSGL : 21
S.japonicus               : -------------------------------------MVSPIVRSVAIIGAGPSGL : 19
S.octosporus              : -------------------------------------MSSSLIRSIAIIGAGPSGL : 19
S.pombe                   : -------------------------------------MCLPTIRKIAIIGAGPSGL : 19
S.punctatus               : -----------------------------------------MKRDILIVGAGPSGL : 15
S.sclerotiorum            : --------------------------------MNFKLRASFNVKKIAIVGAGPSGL : 24
T.rubrum                  : -----------------------------------MTRATPPVRKVAIIGAGACGL : 21
U.reesii                  : ----------------------------------MSPQTFSNIRKVAIIGAGAGGL : 22
V.alfalfae                : --------------------------------MGSYAPLPFDIKKIAIIGAGPCGL : 24
V.dahliae                 : -------------------------------MSPHGLESRLHVSRVAVIGAGASGL : 25
```
