# Supplementary figures and images for: Characterization of indole-3-pyruvic acid pathway-mediated biosynthesis of auxin in Neurospora crassa
Source: PLoS One. 2018 Feb 8;13(2):e0192293. doi: 10.1371/journal.pone.0192293 (PMC5805262; doi:10.1371/journal.pone.0192293)

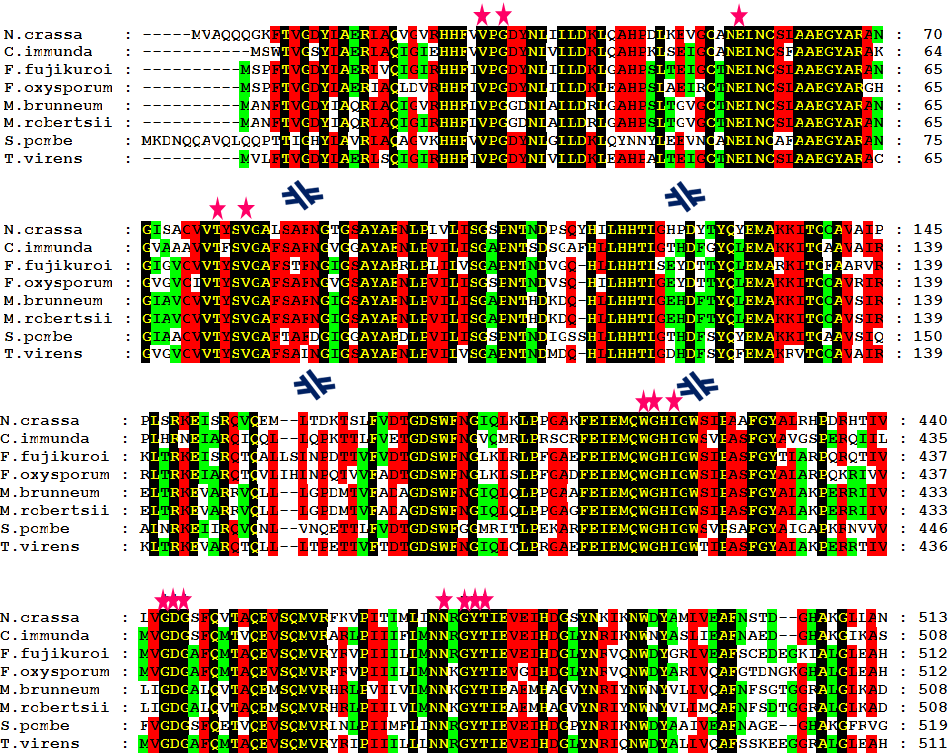

Supplement: S5 Fig — A black background with yellow font, red background with black font and green background with black font represents 100%, 75% and 50% identity of the residues, respectively. TPP binding residues are denoted by pink stars. A blue discontinuous symbol shows a discontinuity in sequence. (TIF) [file pone.0192293.s005.tif]

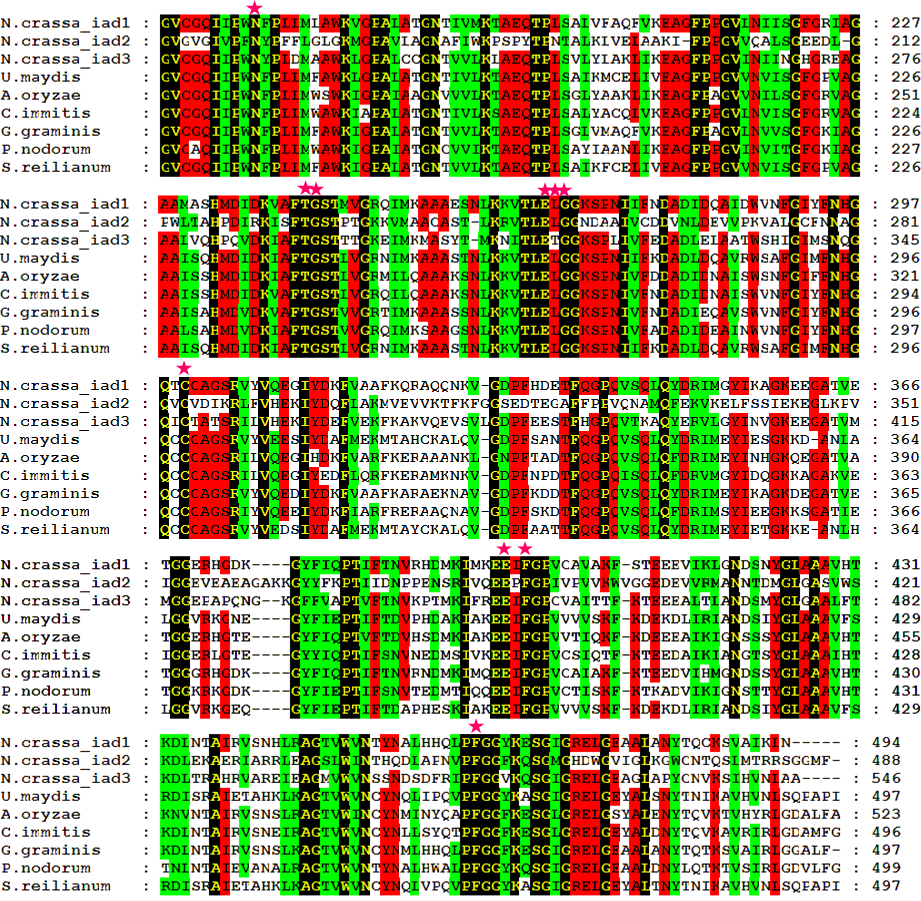

Supplement: S6 Fig — Amino acid residues in yellow font on a black background represent 100% sequence identity, in black font on a red background represent 75% identity and in black font on a green background represent 50% sequence identity. NAD serves as cofactor for the enzyme aldehyde dehydrogenase. NAD binding residues are indicated by a pink star on top of the residues. (TIF) [file pone.0192293.s006.tif]

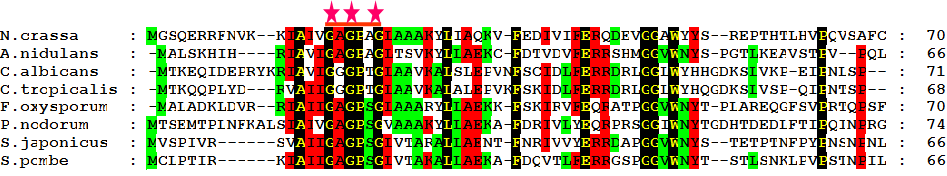

Supplement: S7 Fig — MOX-2 or flavin monooxygenase is known to bind to FAD through its nucleotide binding motif (GXGXXG). GXGXXG motif is shown as a bar, and conserved residues are highlighted by pink stars. Residues in yellow font on a black background are 100% identical and residues, and residues in black font on a red background are 75% identical. (TIF) [file pone.0192293.s007.tif]

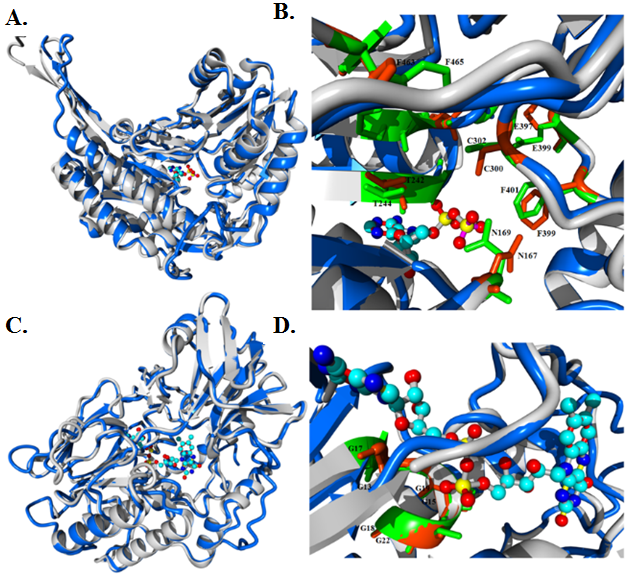

Supplement: S8 Fig — Predicted structures and known templates are shown in blue and gray, respectively. Residues present on predicted enzymes and templates are marked with orange and green, respectively. All structures are rendered as a ribbon. Key amino acid residues involved in ligand binding are rendered as a stick model. Residues those are at close vicinity are only highlighted. Ligands are shown as a ball and stick model. (a) Overall structural alignment of the predicted CBS-3 structure with the known enzyme structure (1NZX). NAD is bound inside enzymatic catalytic site. (b) Structural insight of the ligand binding site of both the predicted and 1NZX known structure. (c) Structural alignment of the predicted structure of flavin monooxygenase homolog from N. crassa with the known flavin monooxygenase structure (1VQW). (d) Characterization of the ligand binding site of the predicted enzyme using the 1VQW structure as a template. (TIF) [file pone.0192293.s008.tif]

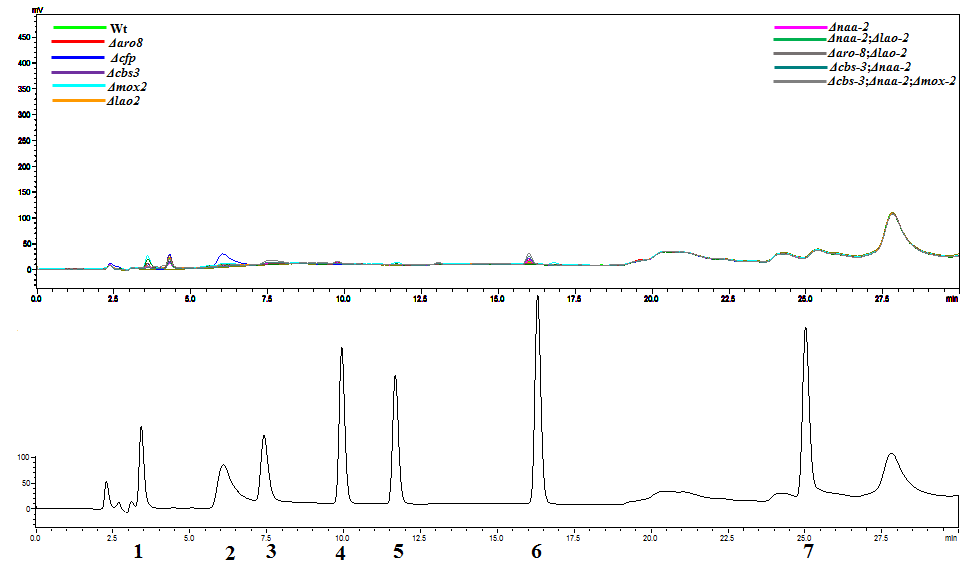

Supplement: S9 Fig — Lower panel of the figure with numbers denotes the standard peaks of the various indolic compounds. Except indole-3-lactic acid (ILA) produced by Δcfp strain, no other indolic compounds comparable to the standard peaks were found within the time window set for the analysis. Genes that were analyzed from different pathways are as follows: aro-8aro-8aro-8, cfp, cbs-3, mox-2, lao-2 (tryptophan-2-monooxygenase) and naa-2 (indole-3-acetamide hydrolase). lao-2 and naa-2 are involved in IAM pathway of IAA biosynthesis. Standard peaks are: lane 1—tryptophan, lane 2—indole-3-lactic acid, lane 3—tryptamine, lane 4—indole-3-acetamide, lane 5—indole-3-acetic acid, lane 6—tryptophol, lane 7 -indole-3-acetonitrile. (TIF) [file pone.0192293.s009.tif]

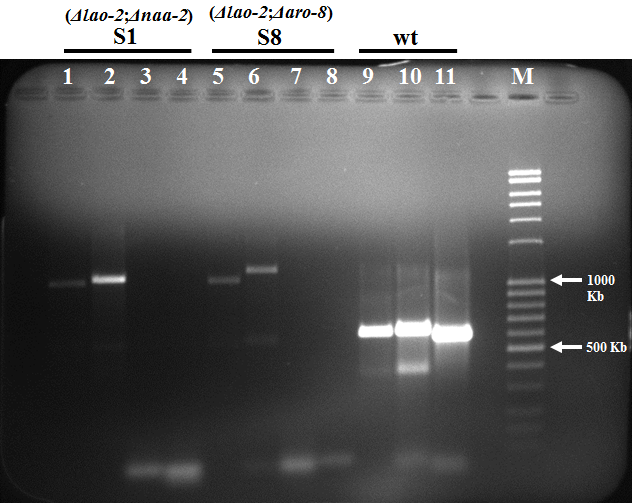

Supplement: S11 Fig — Two samples, namely, S1 and S8, were confirmed. S1 is a double knock-out strain for genes lao-2 and naa-2, and S8 is a double knock-out strain for genes lao-2 and aro-8. Lane 1 and 2 were loaded with the PCR products of 5’ UTR amplifications of lao-2 and naa-2 ORFs, respectively, along with the hph cassette. Lane 3 and 4 were loaded with the PCR products of mid-part of lao-2 and naa-2 gene ORFs respectively. Lane 5 and 6 were loaded with the PCR products of 5’ UTR amplifications of lao-2 and aro-8 ORFs, respectively, along with the hph cassette. Lane 7 and 8 were loaded with the PCR products of mid-part of lao-2 and aro-8 ORFs respectively. Lane 9, 10 and 11 were loaded with the PCR products of mid-part of lao-2, naa-2 and aro-8 ORFs, respectively, from wild type strain (wt). M—DNA marker. (TIF) [file pone.0192293.s011.tif]

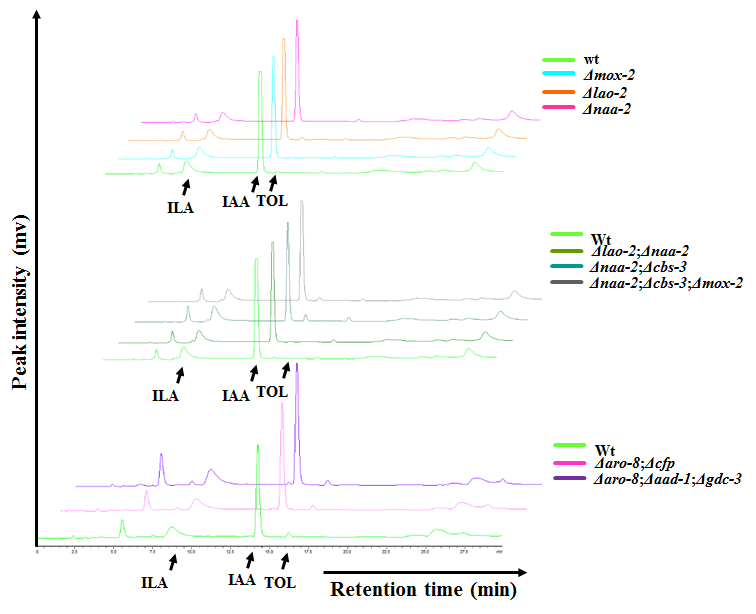

Supplement: S12 Fig — Graphs with different colors represent different knock-out strains. A specific graph for a particular knock-out strain is mentioned in the figure with color bars. Standard peaks are ILA: indole-3-lactic acid, IAA: indole-3-acetic acid and TOL: tryptophol. (TIF) [file pone.0192293.s012.tif]

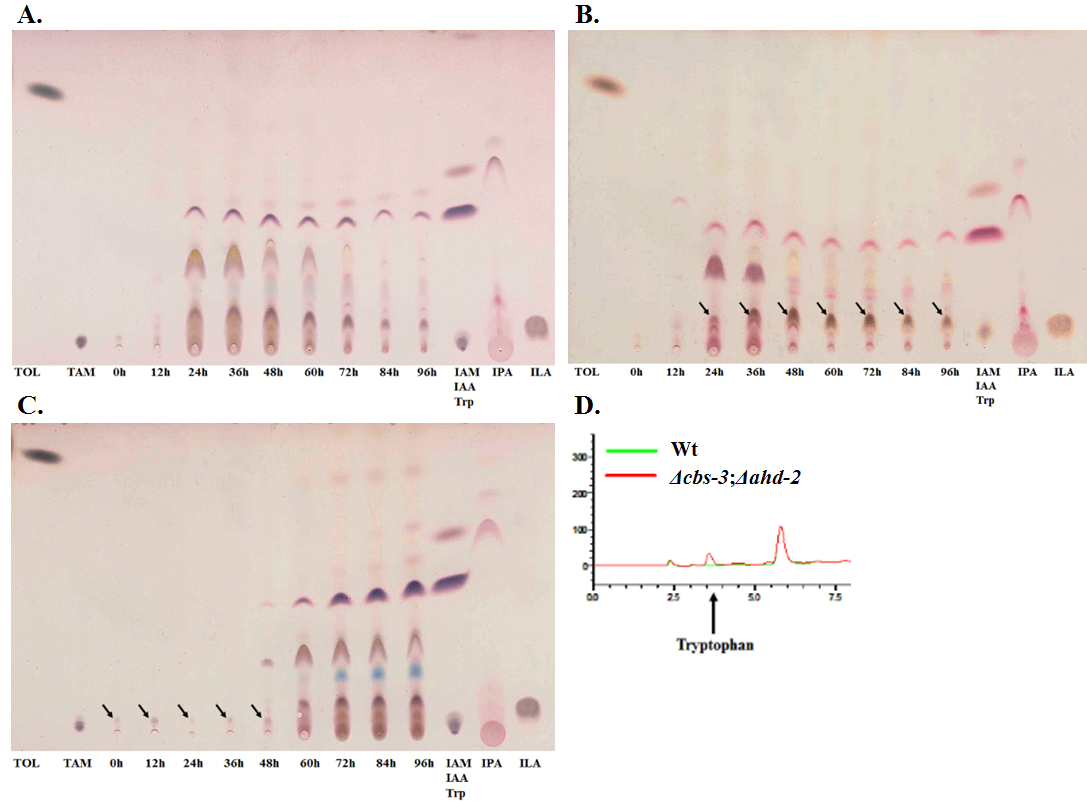

Supplement: S13 Fig — Changes in IAA and indolic compounds metabolism over time in wild type and mutant N. crassa supplemented with 1.96 mM tryptophan. (A) Wild type N. crassa strain was grown with tryptophan supplementation, and samples were collected in every 12 hours for a total of 96 hours. The following standards were used: tryptophol (TOL), tryptamine (TAM), indole-3-acetamide (IAM), indole-3-acetic acid (IAA), tryptophan (Trp), indole-3-pyruvic acid (IPA) and indole-3-lactic acid (ILA). (B) Δcfp knock-out strain was grown with tryptophan supplementation. At every 12-hour interval, samples were collected. Black arrow marks indicate ILA spots. The following standards were used: tryptophol (TOL), indole-3-acetamide (IAM), indole-3-acetic acid (IAA), tryptophan (Trp), indole-3-pyruvic acid (IPA) and indole-3-lactic acid (ILA). (C) The Δcbs-3Δahd-2 double knock-out strain was grown with tryptophan supplementation. At every 12-hour interval, samples were collected and analyzed. Black arrow marks indicate tryptophan spots. The following standards were used: tryptophol (TOL), indole-3-acetamide (IAM), indole-3-acetic acid (IAA), tryptophan (Trp), indole-3-pyruvic acid (IPA), and indole-3-lactic acid (ILA). (D) HPLC analysis of 48 hours old wild type and Δcbs-3;Δahd-2 double knock-out N. crassa culture supplemented with tryptophan. After 48 hours of inoculation Δcbs-3;Δahd-2 double knock-out strain shows a peak for tryptophan while corresponding peak from wild type strain has not been observed. (TIF) [file pone.0192293.s013.tif]

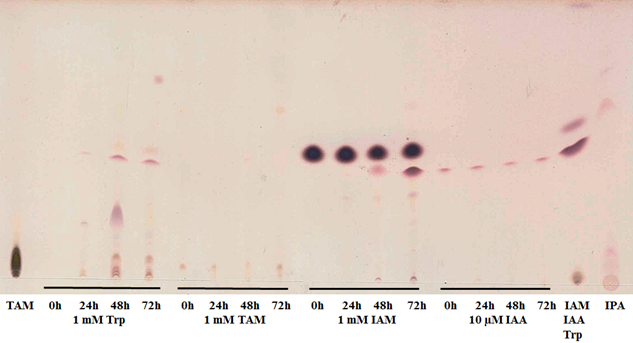

Supplement: S14 Fig — Different indolic compounds were supplemented to wild type N. crassa culture media for checking their role in IAA production over time. tryptophan (Trp), tryptamine (TAM), indole-3-acetamide (IAM) and indole-3-acetic acid (IAA) were used for the assay. Samples were collected in 24-hour intervals. The numbers below the lanes represent the time in hours. Following standards were used: tryptamine (TAM), indole-3-acetamide (IAM), indole-3-acetic acid (IAA), tryptophan (Trp), and indole-3-pyruvic acid (IPA). (TIF) [file pone.0192293.s014.tif]

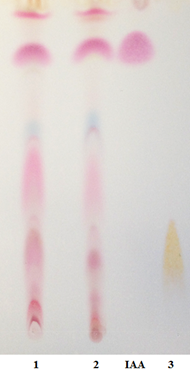

Supplement: S15 Fig — Fungal culture was supplemented with 2 mM histidine. Lane 1: Fungal culture was supplemented with 1.96 mM of tryptophan. Lane 2: Fungal culture was supplemented with 1 mM of tryptophan. Lane 3: Fungal culture was supplemented with 2 mM of histidine. IAA: indole-3-acetic acid standard. (TIF) [file pone.0192293.s015.tif]
