# Supplementary material for: Characterization of indole-3-pyruvic acid pathway-mediated biosynthesis of auxin in Neurospora crassa
Source: PLoS One. 2018 Feb 8;13(2):e0192293. doi: 10.1371/journal.pone.0192293 (PMC5805262; doi:10.1371/journal.pone.0192293)
Supplement: S10 Fig — Different tryptophan concentrations ranging from 2 mM to 12.5 μM were used. (PDF) [file pone.0192293.s010.pdf]

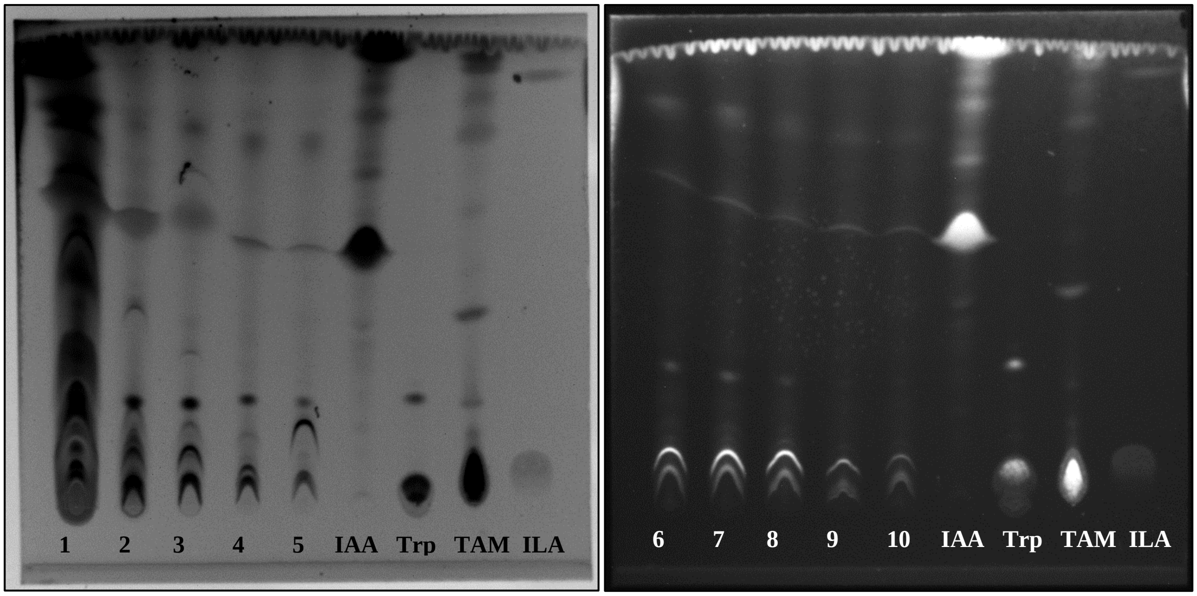

Spot No.	Concentration	Total volume	Strain
**1**	2 mM	50 ml	Wt
**2**	1 mM	50 ml	Wt
**3**	0.5 mM	50 ml	Wt
**4**	0.25 mM	50 ml	Wt
**5**	125 µM	50 ml	Wt
**6**	125 µM	50 ml	Wt
**7**	100 µM	50 ml	Wt
**8**	50 µM	50 ml	Wt
**9**	25 µM	50 ml	Wt
**10**	12.5 µM	50 ml	Wt
