# Supplementary material for: Characterization of indole-3-pyruvic acid pathway-mediated biosynthesis of auxin in Neurospora crassa
Source: PLoS One. 2018 Feb 8;13(2):e0192293. doi: 10.1371/journal.pone.0192293 (PMC5805262; doi:10.1371/journal.pone.0192293)
Supplement: S1 Table — (PDF) [file pone.0192293.s016.pdf]

Detailed specification of *Neurospora crassa* strains:

Serial	FGSC Number	Genotype/ Locus	Classical Gene name	*N. crassa* Gene name	Mating Type	Linkage Group
1.	2489	*N. crassa* 74-OR23-1VA	-		A	-
2.	4200	*N. crassa*	-		a	-
3.	12555	NCU06112	*tdc2*	*gdc3*	a	VII
4.	12556	NCU06112	*tdc2*	*gdc3*	A	VII
5.	12919	NCU00378	*iad3*	*ahd2*	a	III
6.	12921	NCU03415	*iad1*	*cbs3*	a	II
7.	12922	NCU03415	*iad1*	*cbs3*	A	II
8.	14354	NCU09648	*iad2*	*ahd3*	A	IV
9.	16277	NCU04092	*ami1*	*naa2*	A	VI
10.	18065	NCU02193	*ipd*	*cfp*	a	VII
11.	18066	NCU02193	*ipd*	*cfp*	A	VII
12.	18224	NCU08275	*tdc1*	*aad1*	a	IV
13.	18225	NCU08275	*tdc1*	*aad1*	A	IV
14.	20239	NCU01106	*iaam*	*lao2*	a	V
15.	21462	NCU03755	*yuc*	*mox2*	a	V
16.	21463	NCU03755	*yuc*	*mox2*	A	V
17.	22136	NCU09116	*tam1*	*aro8*	A	I
