# Supplementary material for: Characterization of indole-3-pyruvic acid pathway-mediated biosynthesis of auxin in Neurospora crassa
Source: PLoS One. 2018 Feb 8;13(2):e0192293. doi: 10.1371/journal.pone.0192293 (PMC5805262; doi:10.1371/journal.pone.0192293)
Supplement: S2 Table — (PDF) [file pone.0192293.s017.pdf]

Description of primers used in PCR to check for the knockout strains

Sr. No.	Primer name	Sequence (5' ----> 3')	Description
1.	PS2660	TCGAATCCATCACCCTCAAG	Fwd. NCU09116 ORF Posn. 392 (*tam1*)
2.	PS2661	AATACGGCTCGTCCTCAATC	Rev. NCU09116 ORF Posn. 1000 (*tam1*)
3.	PS2662	AAACCTCTACCGGTCTCTTC	Fwd. NCU01106 ORF Posn. 773 (*iaam*)
4.	PS2663	CGTCTTTAGAGGGCAAAGTC	Rev. NCU01106 ORF Posn. 1390 (*iaam*)
5.	PS2670	AACCCGGTTCACGGTACTCC	Fwd. NCU04092 ORF Posn. 796 (*ami1*)
6.	PS2671	AGGTCAGGTTGCCGGTTTCC	Rev. NCU04092 ORF Posn. 1375 (*ami1*)
7.	PS2738	TAGGAGGGCGTGGATATGTC	Fwd. HygB ORF posn. 122
8.	PS2739	CGTCTGCTGCTCCATACAAG	Rev. HygB ORF posn. 738
9.	PS2740	GCAGAACCAATGACCTTAGC	Fwd. to amplify NCU01106 (*iaam*) 5’ UTR along with HygB gene
10.	PS2741	ATCCACTTAACGTTACTGAAATCTCCAAC	Rev. specifically on HygB gene
11.	PS2742	CCTGAACTGGTATCGAAAGG	Fwd, to amplify NCU09116 (*tam1*) 5’ UTR along with HygB gene
12.	PS2743	ATGCTATCTGACCTGAGACG	Fwd. to amplify NCU04092 (*ami1*) 5’ UTR along with HygB gene
13.	PS2755	CAAGTGCGCTGCCGAGTATC	Fwd. (NCU02193) ORF posn 615 (*ipd*)
14.	PS2756	CGACGAACAGGCTGGTCTTG	Rev. (NCU02193) ORF posn 1171 (*ipd*)
15.	PS2757	AAGTGTTAGGTCGCAGTAGG	Fwd. to amplify NCU02193 (*ipd*) 5’ UTR along with HygB gene
16.	PS2760	CCTATGATGCCGTTGTCGTG	Fwd. (NCU03755) ORF posn 521 (*yuc*)

17.	PS2761	AAATCGCTGCCTGACTCTCC	Rev. (NCU03755) ORF posn 1078 (*yuc*)
18.	PS2809	GCTCATTTACGGTCTGTTCC	Fwd. to amplify NCU03755 (*yuc*) 5‘ UTR along with HygB gene
19.	PS2811	CTATTGATCGTCAGCCAAGG	Fwd. to amplify NCU03415 (*iad1*) 5’ UTR along with HygB gene
20.	PS2919	CTGGCGTTCTCAACATCATC	Fwd. Primer for *iad1* (NCU03415) ORF Posn. 638
21.	PS2920	GATGAAGTAGCCCTTGTCAC	Rev. Primer for *iad1* (NCU03415) ORF Posn. 1137
22.	PS2928	AACTACCCGCTAGATATGGC	Fwd primer for mid part of *iad3* ORF
23.	PS2929	CCTCTTCCTTTCCGACATTG	Rev primer for mid part of *iad3* ORF
24.	PS2930	GTTGGACGACGAGGTGATTG	Fwd primer for mid part of *iad2* ORF (posn. 454)
25.	PS2931	CGCCGAACTTGAACGTCTTG	Rev primer for mid part of *iad2* ORF (posn. 1028)
26.	PS2936	AGGTACCAACAGTACCATGC	Fwd. To amplify NCU00378 (*iad3*) 5’ UTR along with HygB gene
27.	PS2937	GTCAGCAGAATACTTGGACC	Fwd. To amplify NCU09648 (*iad2*) 5’ UTR along with HygB gene
